# Generation and Characterisation of Cisplatin-Resistant Non-Small Cell Lung Cancer Cell Lines Displaying a Stem-Like Signature

**DOI:** 10.1371/journal.pone.0054193

**Published:** 2013-01-17

**Authors:** Martin P. Barr, Steven G. Gray, Andreas C. Hoffmann, Ralf A. Hilger, Juergen Thomale, John D. O’Flaherty, Dean A. Fennell, Derek Richard, John J. O’Leary, Kenneth J. O’Byrne

**Affiliations:** 1 Thoracic Oncology, Institute of Molecular Medicine, Trinity Centre for Health Sciences St. James’s Hospital & Trinity College Dublin, Dublin, Ireland; 2 Molecular Oncology Risk-Profile Evaluation (M.O.R.E.), Department of Medical Oncology, West German Cancer Centre, University Hospital Essen, Essen, Germany; 3 Department of Cell Biology (Cancer Research), University Duisburg-Essen, Essen, Germany; 4 Thoracic Medical Oncology, University of Leicester & Leicester University Hospitals, Leicester, United Kingdom; 5 Institute of Health & Biomedical Innovation, Queensland University of Technology, Brisbane, Australia; 6 Department of Histopathology, St. James’s Hospital & Trinity College Dublin, Dublin, Ireland; National Taiwan University Hospital, Taiwan

## Abstract

**Introduction:**

Inherent and acquired cisplatin resistance reduces the effectiveness of this agent in the management of non-small cell lung cancer (NSCLC). Understanding the molecular mechanisms underlying this process may result in the development of novel agents to enhance the sensitivity of cisplatin.

**Methods:**

An isogenic model of cisplatin resistance was generated in a panel of NSCLC cell lines (A549, SKMES-1, MOR, H460). Over a period of twelve months, cisplatin resistant (CisR) cell lines were derived from original, age-matched parent cells (PT) and subsequently characterized. Proliferation (MTT) and clonogenic survival assays (crystal violet) were carried out between PT and CisR cells. Cellular response to cisplatin-induced apoptosis and cell cycle distribution were examined by FACS analysis. A panel of cancer stem cell and pluripotent markers was examined in addition to the EMT proteins, c-Met and β-catenin. Cisplatin-DNA adduct formation, DNA damage (γH2AX) and cellular platinum uptake (ICP-MS) was also assessed.

**Results:**

Characterisation studies demonstrated a decreased proliferative capacity of lung tumour cells in response to cisplatin, increased resistance to cisplatin-induced cell death, accumulation of resistant cells in the G0/G1 phase of the cell cycle and enhanced clonogenic survival ability. Moreover, resistant cells displayed a putative stem-like signature with increased expression of CD133+/CD44+cells and increased ALDH activity relative to their corresponding parental cells. The stem cell markers, Nanog, Oct-4 and SOX-2, were significantly upregulated as were the EMT markers, c-Met and β-catenin. While resistant sublines demonstrated decreased uptake of cisplatin in response to treatment, reduced cisplatin-GpG DNA adduct formation and significantly decreased γH2AX foci were observed compared to parental cell lines.

**Conclusion:**

Our results identified cisplatin resistant subpopulations of NSCLC cells with a putative stem-like signature, providing a further understanding of the cellular events associated with the cisplatin resistance phenotype in lung cancer.

## Introduction

More than one million cases of lung cancer are diagnosed each year. The disease is the leading cause of cancer-related death in men and women [1]. Despite intensive efforts to control morbidity and mortality from lung cancer, the overall five-year survival rate remains poor.

Cisplatin, *cis*-Diamminedichloro-platinum(II), is one of the most commonly used chemotherapeutic agents in the treatment of cancer, in particular non-small cell lung cancer (NSCLC) [2]. The cytotoxic effects of cisplatin are mediated by its interaction with DNA, resulting in the formation of DNA adducts which activate several signal transduction pathways and culminate in the activation of apoptosis [3]. While 20–40% of patients with metastatic NSCLC experience a partial response to newly developed combination therapies [4], most responders relapse within six months [5]. Within the population of patients that relapse, the selection of pre-existing resistant cells and/or acquisition of resistant cells during treatment with chemotherapy has been proposed. Therefore, a better understanding of the molecular basis of cisplatin resistance is warranted in order to elucidate the mechanisms and markers underlying this drug-resistant phenotype, which at present radically limits the clinical utility of this drug in lung cancer patients.

Recently, the cancer stem cell (CSC) theory was proposed to explain tumour heterogeneity and carcinogenesis [6]. According to this model, tumours may be viewed as a result of abnormal organogenesis driven by CSC’s. These are self-renewing tumour cells that are able to initiate and maintain tumour growth through subpopulations of tumour cells with stem or progenitor cell characteristics. Using *in vitro* systems and *in vivo* models of human primary lung cancer xenografts in mice, recent research has demonstrated that lung tumour cells expressing specific CSC markers were highly tumourigenic, endowed with stem-like features and spared by treatment with cisplatin [7].

In this study, we have generated and characterised a panel of cisplatin resistant NSCLC cell lines, providing a valuable tool with which to investigate the molecular pathways and putative stem cells markers that may be associated with this resistance phenotype in lung cancer.

## Materials and Methods

### Cell Lines

The human large cell lung cancer cell line, NCI-H460 (hereafter referred to as H460) and its resistant variant was kindly donated by Dr Dean Fennell, Centre for Cancer Research and Cell Biology, Queen’s University Belfast [8]. The human adenocarcinoma cell line, MOR [9], and its corresponding cisplatin resistant variant was obtained from the American Type Culture Collection (ATCC) (LGC Promochem, Teddington, UK). A549 (adenocarcinoma) and SKMES-1 (squamous carcinoma) cell lines were also purchased from the ATCC [10], [11]. MOR and H460 cells were grown in Roswell Park Memorial Institute (RPMI-1640) medium. A549 cells were cultured in Ham’s F12 media supplemented with 4 mM L-glutamine while SKMES-1 cells were cultured in EMEM media supplemented with 2 mM L-glutamine and 1% non-essential amino acids (NEAA). For all cell lines, media was supplemented with 10% heat-inactivated fetal bovine serum (FBS), penicillin (100 U/ml) and streptomycin (100 µg/ml) (Lonza, United Kingdom). All cells were grown as monolayer cultures and maintained in a humidified atmosphere of 5% CO_2_ in air at 37°C.

### Drugs

Cisplatin [*cis*-diammineplatinum(II) dichloride] was obtained from Sigma-Aldrich and dissolved in 0.15 M NaCl. Aliquots were stored at −20°C for up to a maximum of three months and thawed immediately before use.

### Induction of Cisplatin-Resistance in NSCLC Cells

Cisplatin-resistant (CisR) variants of each cell line were derived from each original parental (PT) cell line by continuous exposure to cisplatin (Sigma-Aldrich, UK) following initial dose-response studies of cisplatin (0.1 µM–100 µM) over 72 h from which IC_50_ values were obtained. Initially, each CisR subline was treated with cisplatin (IC_50_) for 72 h. The media was removed and cells were allowed to recover for a further 72 h. This development period was carried out for approximately 6 months, after which time IC_50_ concentrations were re-assessed in each resistant cell line. Cells were then maintained continuously in the presence of cisplatin at these new IC_50_ concentrations for a further 6 months. While A549 cells were initially treated with IC_50_ concentrations of cisplatin, cells were sensitive to treatment at this concentration resulting in cell senescence and delayed growth. For this reason, the cisplatin concentration was reduced (IC_25_) until such time as cells demonstrated sensitivity to cisplatin at the appropriate IC_50_ concentration.

### Drug Sensitivity Assay (MTT)

Cells (2.5×10^3^) were seeded in 96-well plates and allowed to adhere overnight at 37°C. Briefly, following treatment of cells with cisplatin for 72 h, MTT reagent [3-(4,5-Dimethylthiazol-2-yl)-2,5-diphenyltetrazolium bromide] was added to each well and incubated for 4 hrs at 37°C. Dimethylsulphoxide (DMSO) was added to each well and mixed for 5 min on an orbital shaker. Absorbance was recorded at 595 nm and sensitivity to cisplatin was calculated based on cell proliferation measurements at 72 h.

### Cell Cycle & Apoptosis Analysis

Cells were collected by trypsinisation, pelleted by centrifugation at 1300 rpm for 3 min and suspended in 1 ml phosphate-buffered saline (PBS). Cells were subsequently fixed in 90% cold ethanol and incubated at room temperature for 30 min. Cells were pelleted and resuspended in 1 ml PBS containing propidium iodide (25 µg/ml) and DNase-free RNase A (100 µg/ml). Following incubation at 37°C for 30 min, cell cycle distribution of PT and CisR cells were analysed using FACS (Becton Dickinson, UK). Apoptotic cells (SubG0) were measured in response to increasing concentrations of cisplatin between PT and CisR cells following treatment for 24 h.

### Clonogenic Survival Assay

The sensitivity of NSCLC cells to cisplatin was measured using the clonogenic assay, the method of choice used to determine the effectiveness of cytotoxic agents such as chemotherapy [12]. Cells were allowed to adhere overnight at 37°C and treated with increasing concentrations of cisplatin for 9–14 days. Colonies were fixed and stained with methanol (25% v/v) containing crystal violet (0.05% w/v) for 30 min after which time residual staining solution was removed and plates were washed with water. Colonies consisting of 100 cells or more were counted using the ColCount™ colony counter (Oxford Optronix Ltd, Oxford, UK). Plating efficiencies (PE) were calculated using the formula: PE = Number of colonies/Number of cells seeded. The surviving fraction (SF) was calculated using the formula: SF = Number of colonies/Number of cells seeded × PE). Survival curves were constructed for determination of survival ability of cisplatin-resistant cells relative to parent cells in response to various concentrations of cisplatin.

### Flow Cytometry Analysis of Putative Cancer Stem Cell Markers

Parent and cisplatin-resistant cells were collected by trypsination and washed in FACS buffer (2% FBS 0.1% sodium azide in PBS) and pelleted by centrifugation at 1300 rpm for 3 min. Dual staining for CD133 and CD44 (epithelial cell marker) was carried out. Cells (1×10^6^) were incubated with either CD133/1 (AC133) phycoerythrin (PE)-labelled antibody or isotype control antibody (IgG1) (Miltenyi Biotec GmbH), or anti-human CD44 FITC-conjugated antibody and corresponding isotype control (IgG2b) (ImmunoTools GmbH, Germany) for 30 min in the dark at 4°C. Cells were washed briefly and resuspended in FACS buffer for subsequent analysis. Samples were acquired and analysed by FACS. Side scatter and forward scatter profiles were used to eliminate debris and cell doublets. The percentage CD133+ and CD44+ cells was determined in PT and CisR cell lines by flow cytometry.

### Aldefluor Assay

The Aldefluor Kit (Stem Cell Technologies, Vancouver, Canada) was used to identify cell populations with aldehyde dehydrogenase (ALDH1) activity. The assay was carried according to manufacturer’s instructions. Briefly, cells (1×10^6^ cell/ml) were harvested from PT and CisR cell lines and resuspended in Aldefluor Assay Buffer and incubated for 60 mins at 37°C. The amount of fluorescent ALDH reaction product that accumulates in the cells directly correlates to the ALDH activity in these cells. Active efflux from the cells is inhibited by the special formulation of the Aldefluor Assay Buffer. For each cell line (PT and CisR), control cells were stained using identical conditions but included a specific ALDH inhibitor, diethylaminobenzaldehyde (DEAB), to serve as a negative control for each experiment. Such cells are recognised by comparing the fluorescence in a test sample to that in a control sample containing DEAB. As only cells with an intact cellular membrane can retain the Aldefluor reaction product, only viable ALDH1-positive cells were identified. The brightly fluourescent ALDH1-expressing cells (ALDH1-positive) were detected in the green fluorescent channel (520–540 nm) of a CyAn_ADP_ flow cytometer (Dako, Glostrup, Denmark) and calculated as the percentage ALDH1-positive cells in each cell line.

### Western Blot Analysis

Total protein was extracted from parent and cisplatin resistant cells using ice-cold RIPA buffer (50 mM Tris HCl, pH 7.4, 150 mM NaCl, 1 mM EDTA, 1% (v/v) Triton-X 100, 0.1% (w/v) SDS) supplemented with phenylmethylsulfonyl fluoride (PMSF) and protease inhibitor cocktail (2 mM AEBSF, 1 mM EDTA, 130 µM Bestatin, 14 µM E-64, 1 µM Leupepin, 0.3 µM Aprotinin). Protein concentrations were determined using the bicinchoninic acid assay as per manufacturer’s instructions (BCA). Protein (40 µg) from whole cell lysates was fractionated on 12% SDS-PAGE gels and transferred to a PVDF membrane (PALL Corporation, FL, USA). Transfer efficiency and loading were confirmed by reversible staining of the membrane with Ponseau S solution (Sigma-Aldrich, UK) following protein transfer. Membranes were blocked at room temperature with 5% non-fat dry milk in Tris-buffered saline (TBS) containing 0.1% Tween-20 (TBS-T) and screened using a human embryonic stem cell marker panel (Abcam plc, United Kingdom). These included primary rabbit polyclonal mouse antibodies to Nanog, Oct-4 and SOX-2 (1∶1000). Protein expression of c-Met (Millipore) and β-Catenin (BD Transduction Laboratories) was also examined using mouse monoclonal antibodies at 1∶100 and 1∶2000, respectively. Membranes were washed in TBST and incubated with a secondary horseradish peroxidase (HRP)-labelled antibody for 1 h at room temperature (1∶2000). Membranes were washed in TBST following incubation with secondary antibodies. Bound antibody complexes were detected and visualised using SuperSignal^®^ West Pico enhanced chemiluminescence substrate (Pierce, IL, USA). Blots were stripped and re-probed with α/β Tubulin antibody (Cell Signalling) to control for loading. Densitometric analysis was carried out using TINA™ software and percentage expression represented relative to controls (100%).

### Immunofluorescence Microscopy and Measurement of Cisplatin-DNA Adducts

Cells (PT and CisR) were treated with cisplatin (IC_50_) for 0, 4, 12 and 24 h after which time they were collected by trypsinization and washed twice in PBS. Cells (1×10^6^ cell/ml) were resuspended in PBS and spotted (10 µl), in triplicate, onto Superfrost Gold Slides (ThermoFisher). Slides were allowed to air dry briefly at room temperature. Immunofluorescence staining and measurement of specific DNA platination products was performed as previously described [13], with minor modifications. Briefly, cells were fixed overnight in ice-cold methanol and subjected to proteolytic digestion with 60 µg/mL pepsin and 40 µg/mL proteinase K (100 µl per spot for 10 min at 37°C in a humidified chamber). Upon blockade of non-specific binding sites with 5% (w/v) non-fat powdered milk in PBS, slides were incubated with a rat primary antibody that specifically recognises CDDP-GpG DNA adducts (RC-18) at 37°C for 2 h or 4°C overnight. Primary antibody binding was detected using an anti-rat Cy3®-labelled antibody (Dianova, Hamburg). Slides were then incubated in 1 µg/ml (w/v) DAPI in PBS for 30 min at RT for nuclear counterstaining. Images were acquired on an Axioplan fluorescence microscope (Carl Zeiss GmbH, Göttingen, Germany) coupled to a C4880 CCD camera (Hamamatsu Photonics, Herrsching, Germany). For the quantification of CDDP-GpG DNA adducts by immunofluorescence microscopy, fluorescence signals were measured by quantitative digital image analysis using the ACAS 6.0 CytometryAnalysis System (ACAS II, Ahrens Electronics, Bargterheide, Germany). Levels of adducts in each sample were calculated as arbitrary fluorescence units (AFU’s), upon normalization of integrated antibody-derived fluorescence from 200 individual nuclei/sample to the corresponding DNA content. Data are presented as the mean AFU ±95% confidence interval (CI) from three independent experiments.

### γH2AX Foci Formation Assay

Cells (5×10^3^) were seeded, in triplicate, in 96-well plates and allowed to adhere overnight. Parent and resistant cells were treated with cisplatin for 0, 4, 8, 12 and 24 h. At each time-point, cell culture media was removed from each well and fixed for 10 min in 100 µl formaldehyde (4% v/v in PBS). Cells were then washed twice in PBS. Blocking buffer (5% goat serum, 3% Triton X-100 in PBS) was added to each well and incubated for 1 h at room temperature. Cells were then incubated overnight at 4°C with a primary rabbit anti-human anti-phospho-histone 2AX (Ser139) antibody (1∶100) (Cell Signalling Technology) in antibody dilution buffer (1% BSA.0.3% Triton X-100 in PBS). Following removal of the primary antibody, cells were washed three times in PBS and incubated with Alexafluor 488-labelled goat anti-rabbit secondary antibody (Invitrogen) (1∶2000) for 1 h at room temperature in the dark. Secondary antibody was removed and cells were washed three times in PBS. Cells were then incubated with Hoechst 33342 nuclear stain (3 µg/ml) for 30 min at 37°C, followed by three washed in PBS. Cells staining for phosphorylated histone 2AX (detected as green fluorescent foci) were imaged by immunofluroescence using high content analysis (GE Healthcare). Ten fields of view per well were acquired using a 20X objective. Nuclear staining was detected using an excitation filter of 360 nm and emission filter of 460 nm, while Alexafluor 488 was detected at 480 nm and 535 nm, respectively. Mean nuclear fluorescence intensity was used as a measure of γH2AX using InCell analyser 1000 image analysis software.

### Quantification of Cellular Cisplatin uptake by ICP-MS

For cisplatin uptake studies, cells (1×10^7^ cells/ml) were seeded in culture flasks and allowed to adhere overnight. Cells were then treated with cisplatin for 24 h. Following treatment, cells were washed in PBS, harvested and counted. For drug uptake analysis, cells (1×10^6^) were suspended in 1% HNO_3_ for 24 h at 70°C. Lysed cells were analysed by inductively coupled plasma mass spectrometry (ICP-MS). ICP-MS provides a quantitative analysis of the concentration of an element in aqueous solution and has a sensitivity of 5 PPT or better for Platinum products. The analyte concentration is proportional to the number of ions of a specific element that reach the mass spectrometer from the vaporised solution at 6000°C. A single ICP-MS measurement represents the average of 20 scans per replicate within five replicates from the same liquid sample, with a very small error (<5%). Cisplatin concentrations reported were averaged across four series of cultures, ensuring that the values are correctly scaled to account for cell population differences and dilutions.

Standard curves were generated by using aqueous serial dilutions of stock solutions traceable back to the standard reference material (SRM) from NIST (National Institute of Standards and Technology). The coefficients of variation ranged from 1 to 4% (intra-assay) and from 5 to 10% (inter-assay).

### Statistical Analysis

Statistical comparison between groups was carried out using analysis of variance (ANOVA). Where the means of two data sets were compared, significance was determined by a two-tailed Students *t-*test. Differences were considered to be statistically significant where p≤0.05. Data is graphically represented as mean ± standard error of the mean (SEM). All data was analysed using GraphPad InStat™ (version 3) statistical software.

## Results

### Generation of IC_50_ Concentrations and Development of NSCLC Cells with a Cisplatin-resistant Phenotype

**Figure 1:**
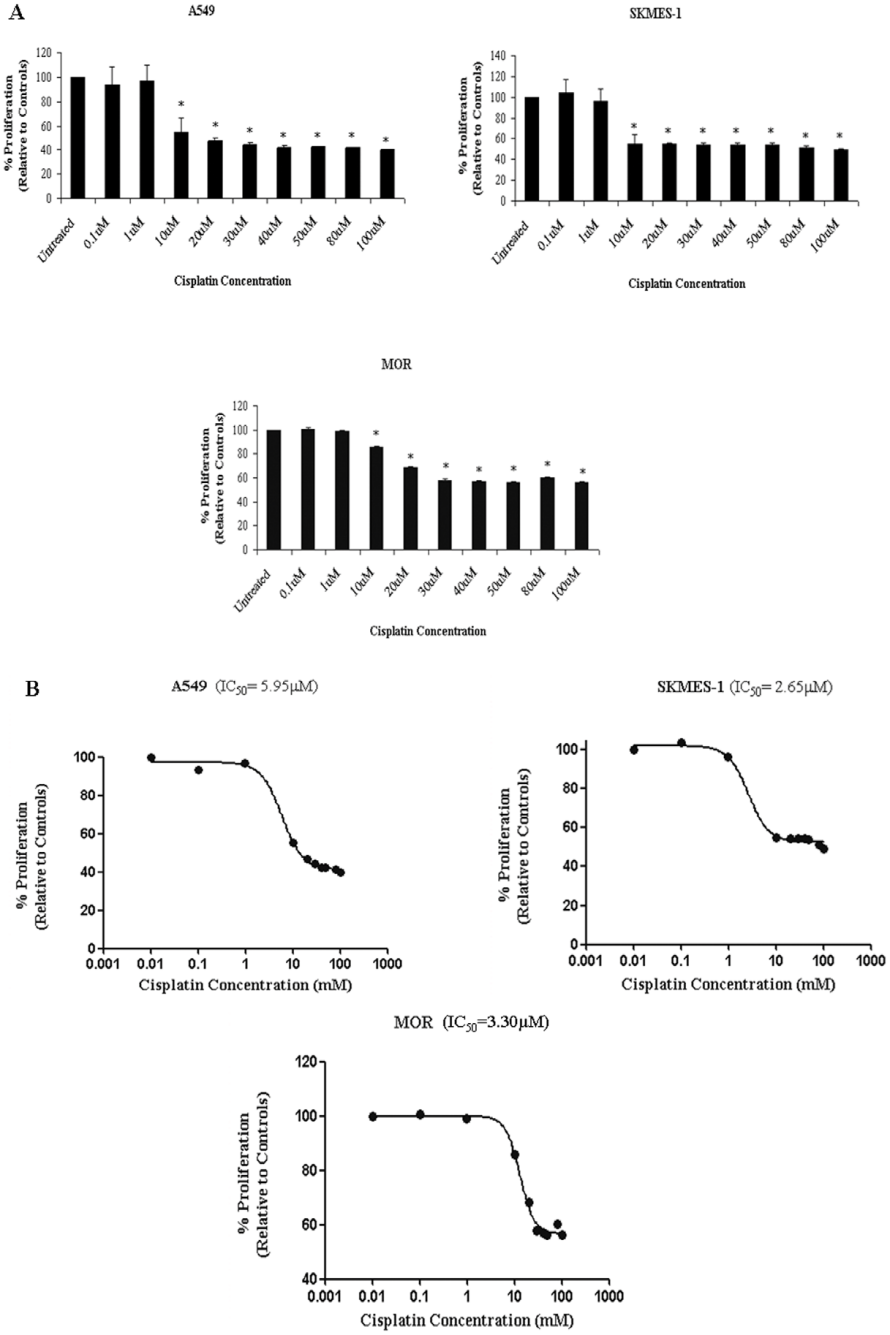
Cisplatin inhibits proliferation of lung cancer cells in a dose-dependent manner. (A) NSCLC cells were treated with increasing concentrations of cisplatin (0.1 µM–100 µM) for 72 h. Cell survival was measured using the MTT assay. Cisplatin significantly reduced proliferation of A549, SKMES-1 and MOR NSCLC cells. (B) Dose-response curves were generated from which IC_50_ values were deduced. Data are expressed as Mean ± SEM from three independent experiments (n = 3) (*p<0.001 vs untreated).

In order to determine IC_50_ values with which to treat parental cell lines in the generation of cisplatin resistant cell lines, cells were treated with increasing concentrations of cisplatin ranging from 0.1 µM to 100 µM. The H460 CisR cell line was previously generated and maintained with 5 µM cisplatin. The sensitivity of each original (PT) cell line to increasing doses of cisplatin was demonstrated, where cisplatin significantly (p<0.001) inhibited proliferation of A549, SKMES-1 and MOR cells at 10 µM–100 µM over 72 h (Fig. 1A). Dose-response curves were generated and IC_50_ concentrations were calculated for all cell lines (Fig. 1B). Cisplatin concentrations (IC_50_) varied between all four cell lines (*A549* 5.95 µM, *SKMES-1* 2.65 µM, *MOR* 3.3 µM, *H460* 5.0 µM) and were subsequently used to treat each parent cell line in order to generate corresponding age and passage-matched cisplatin resistant cell lines. In the case of H460 cells, maintenance of the resistant subline was continued at 5 µM. Treatment of A549 cells with cisplatin (IC_50_) resulted in significant growth delay, with slow recovery periods. Cells were therefore treated with IC_25_ concentrations for several weeks prior to selection of a cisplatin resistant subline at the IC_50_ concentration.

**Figure 2:**
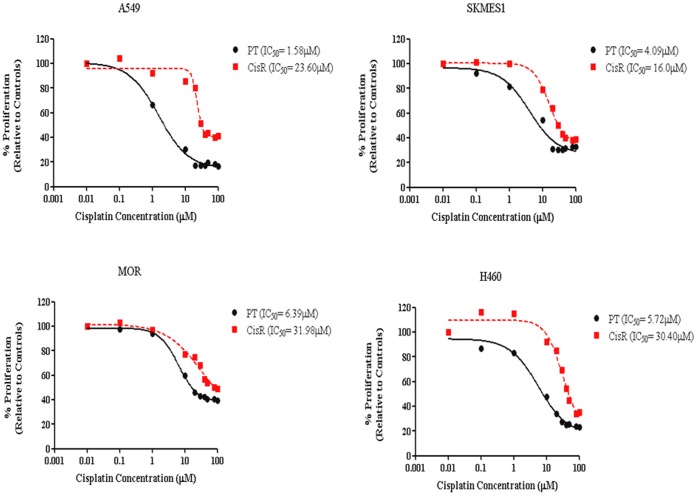
Cells exhibit increased fold changes in IC_50_ concentrations following long-term exposure to cisplatin. Following maintenance of cisplatin treated sublines in culture for 6 months with cisplatin, IC_50_ concentrations were re-assessed for each cell line using dose-response curves generated by GraphPad Prism software. A significant increase in IC_50_ concentration was determined for each cisplatin resistant cell line relative to that for the corresponding age-matched parental cell line.

Cisplatin resistant sublines were treated with cisplatin for 72 h after which time media was removed and cells were allowed to recover and re-populate. During this time, cell survival/proliferation was assessed between PT and CisR cells every 4 weeks to determine changes in sensitivity to cisplatin. At 6 months, IC_50_ values were re-evaluated and deduced from dose-response curves between PT and CisR cells. A significant fold increase was observed in the concentration of cisplatin required to inhibit cells by 50% in cisplatin resistant cells relative to their corresponding parent cells (Fig. 2). Cells were subsequently maintained in cisplatin at these concentrations for a further 6 months. In A549 cells, the IC_50_ concentration of cisplatin resistant cells was determined as 23.60 µM compared to 1.58 µM in the original parent cell line, a 15-fold increase in the concentration of cisplatin required to obtain a 50% inhibition in cell growth. A significant increase in IC_50_ concentrations was also observed in SKMES-1 cells (16.0 µM vs 4.09 µM), MOR cells (31.98 µM vs 6.39 µM) and H460 cells (30.40 µM vs 5.72 µM), demonstrating a 4-fold (SKMES-1) and 5-fold (MOR, H460) increase between CisR and PT cell lines. Taken together, these initial data demonstrated a cisplatin-resistant phenotype in four NSCLC cell lines following continuous *in vitro* exposure to cisplatin.

**Figure 3:**
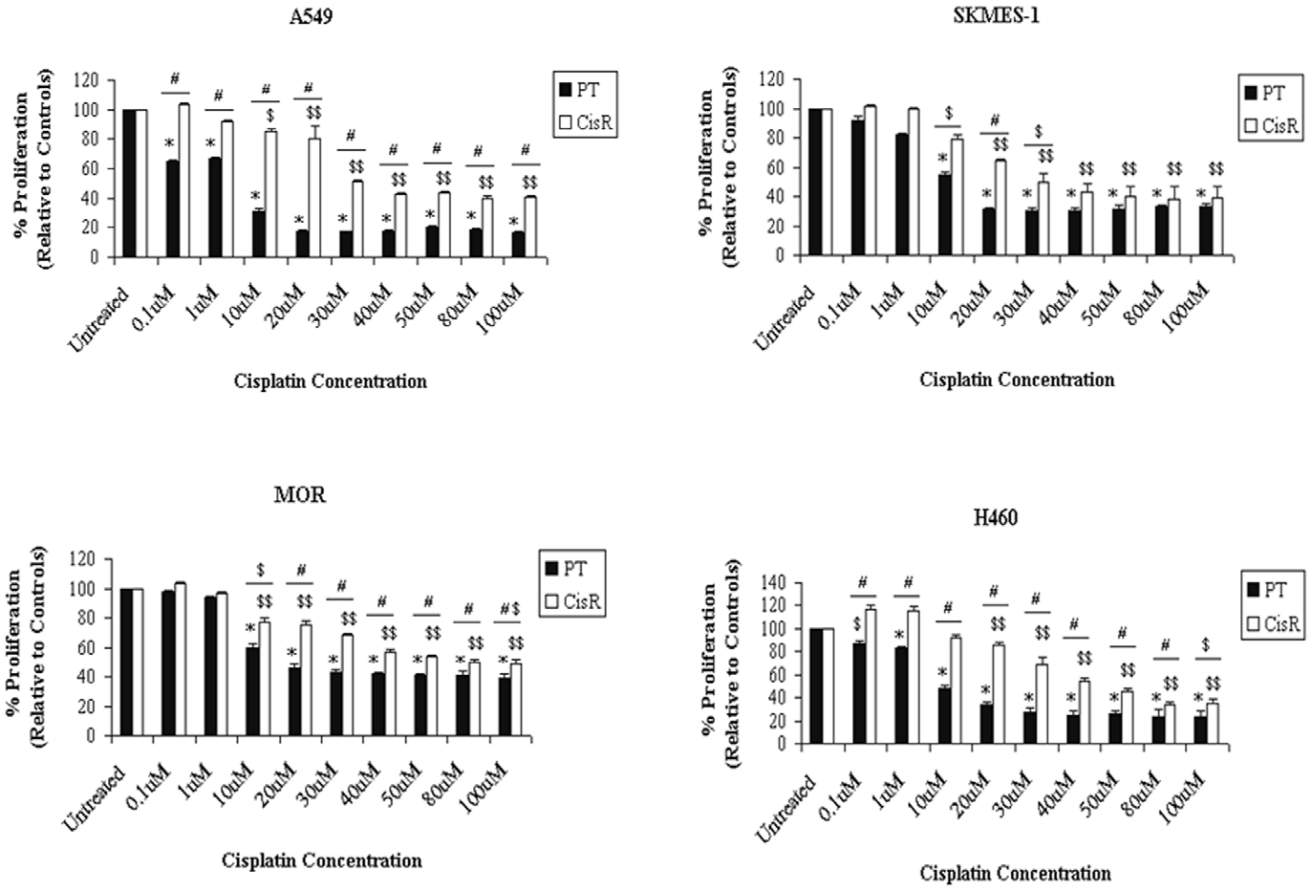
The inhibitory effects of cisplatin on the proliferative capacity of cisplatin resistant NSCLC cells. Parent (PT) and cisplatin resistant (CisR) cell lines were treated with increasing concentrations of cisplatin for 72 h. Proliferation was measured using the MTT assay. While cisplatin inhibited the growth of both PT and CisR cell lines, the inhibitory effect of cisplatin was greatly reduced in CisR cells relative to parent cells. Data are expressed as Mean ± SEM from three independent experiments (n = 3) (*p<0.001 vs PT untreated, ^$$^p<0.001 vs CisR untreated, ^#^p<0.001 PT vs CisR,^ $^p<0.01 vs CisR untreated [A549], ^$^p<0.01 PT vs CisR, ^#$^p<0.05 PT vs CisR [MOR]).

Upon characterisation of cells at 52 weeks following exposure of cells to cisplatin, a significant difference in the proliferation capacity between PT cell lines and their corresponding cisplatin resistant sublines was observed (Fig. 3) indicating the emergence of a resistant phenotype in the resistant sublines relative to the parent cell lines. While A549 and H460 cells showed significant differences in their proliferative ability between parental and corresponding CisR cells at concentrations ranging from as low as 0.1 µM (*A549*, 65.56±0.73 vs 102.50±0.87; *H460*, 87.66±0.67 vs 114.06±1.57, p<0.001) to 100 µM (*A549*, 16.56±0.29 vs 41.44±0.94, p<0.001; *H460*, 20.89±1.22 vs 34.32±1.17, p<0.01), a significant difference was also observed between parent and CisR SKMES-1 and MOR cells at concentrations of cisplatin ranging from 10 µM (*SKMES-1*, 54.38±1.56 vs 79.00±2.25; *MOR*, 64.33±2.33 vs 76.87±2.77, p<0.01) to 100 µM in SKMES-1 and MOR cells, respectively (*SKMES-1*, 32.79±1.55 vs 59.33±5.20, p<0.01; *MOR*, 34.33±2.50 vs 45.33±2.33, p<0.05).

### Cisplatin-induced Apoptosis is Significantly Abrogated in Resistant Cells Relative to their Parent Counterparts

**Figure 4:**
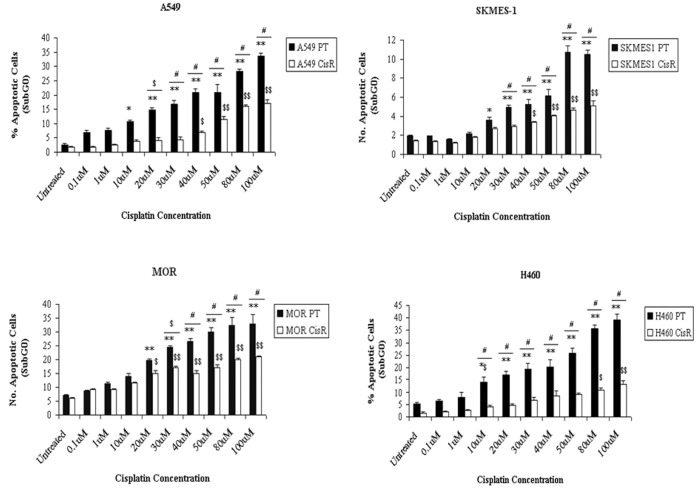
Cisplatin-induced apoptosis is reduced in cisplatin resistant cell lines. Parent and resistant cell lines were treated with cisplatin for 72 h. Apoptotic cells, as measured by the percentage cells in the SubG0 phase of the cell cycle, were measured. Levels of apoptosis induced by cisplatin were significantly increased in parent cells while cisplatin-induced apoptosis was significantly reduced in the corresponding resistant cell line. Data are expressed as Mean ± SEM from three independent experiments (n = 3) (*p<0.01 vs PT untreated, **p<0.001 vs PT untreated, ^$$^p<0.001 vs CisR untreated, ^$^p<0.01 vs CisR untreated [A549, H460], ^$^p<0.05 vs CisR untreated [MOR], ^$^p<0.05 PT vs CisR, ^#^p<0.001 PT vs CisR, *^$^p<0.05 vs PT untreated).

Levels of cisplatin-induced apoptosis, as determined using the SubG0 (apoptotic) fraction of cells, were assessed in PT and corresponding CisR cell lines following treatment of cells with increasing doses of cisplatin. While there was a significant increase in lung tumour cell apoptosis of PT cells in response to cisplatin at concentrations between 10 µM and 100 µM, cisplatin-induced apoptosis of CisR cells was significantly decreased across all cell lines (Fig. 4), in particular A549, SKMES-1 and H460 cells. In A549 and SKMES-1 cells, significant cell death was observed only at higher concentrations between 40 µM (*A549*, p<0.01; *SKMES-1*, p<0.01) and 100 µM (p<0.001). More significantly however, H460 CisR cells displayed greater resistance to cisplatin-induced death at higher concentrations of cisplatin compared to other cell lines, where significant induction of apoptosis was seen in response to cisplatin at concentrations as high as 80 µM (p<0.01) and 100 µM (p<0.001). In all lung tumour cell lines, a significant difference in the cellular response to cisplatin-induced apoptotic cell death was observed between CisR and PT cells. Significant differences in the levels of apoptosis between H460 PT and CisR cells were seen in response to cisplatin at all concentrations ranging from 10 µM to 100 µM, thereby highlighting a greater cisplatin resistant phenotype in this CisR cell line.

### Cisplatin Resistant NSCLC Cells Accumulate in G0/G1 of Cell Cycle

**Figure 5:**
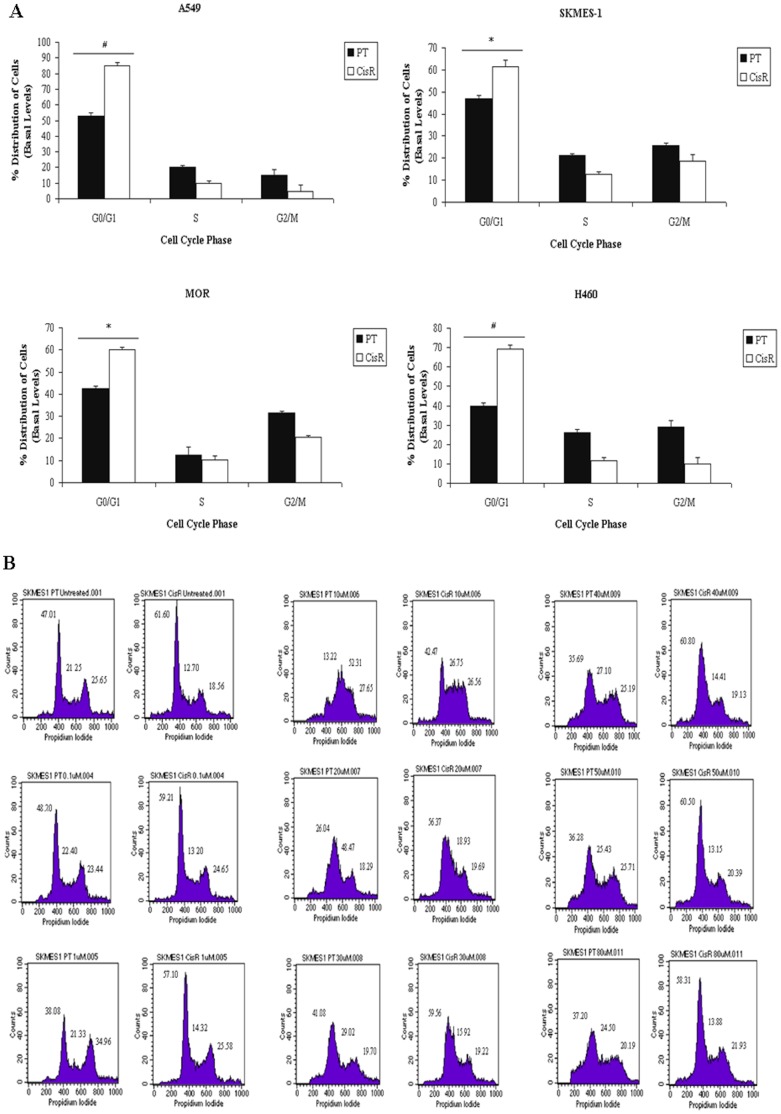
Cisplatin resistant cells accumulate in the G0/G1 phase of the cell cycle. Parent and Cisplatin resistant NSCLC cells were treated with cisplatin for 24 h. Cell cycle distribution of PT and CisR cells was examined by propidium iodide staining and measured by FACS (A). A significant accumulation of CisR cells was observed in the G0/G1 phase of the cell cycle across the panel of cell lines. Representative histograms showing cell cycle distribution of SKMES-1 CisR cells and PT counterparts in response to increasing concentrations of cisplatin are shown (B). Data are expressed as Mean ± SEM from three independent experiments (n = 3) (^#^p<0.001, ^*^p<0.05).

At basal levels, and in response to increasing concentrations of cisplatin, an increased accumulation of cells in the G0/G1 phase was observed in all CisR cell lines relative to their respective parental cell lines (Fig. 5A). Representative histograms are shown for SKMES-1 PT and CisR cells in response to increasing concentrations of cisplatin (Fig. 5B). In cisplatin resistant cell lines, treatment with cisplatin induced a significant accumulation of cells in the G0/G1 phase, relative to PT cells treated at the same concentrations. Such observations were concomitant with a decrease in the S phase of the cell cycle. The basal fractions of cells between G0/G1, S and G2/M phases of the cell cycle were also studied between PT and CisR cell lines. A significant increase in the G0/G1 fraction was found in SKMES-1 CisR (61.60±2.734, p<0.05) and MOR CisR (60.20±0.872, p<0.05) cells relative to their parent counterparts (47.01±1.549, 42.44±1.351, p<0.05). In A549 and H460 cisplatin-resistant cells, greater significance was seen in the G0/G1 fraction relative to their parent counterparts (*A549*, 85.19±1.763 vs 52.83±2.234, *H460*, 69.12±1.987 vs 39.61±2.00, p<0.001). At basal levels, the fraction of cells in the S and G2/M phases did not change significantly between PT and CisR cell lines. These alterations in cell cycle distribution may play an important role in the cisplatin resistant phenotype of the NSCLC cell lines generated.

### Chemoresistant NSCLC Cells Demonstrate Enhanced Clonogenic Survival Ability

**Figure 6:**
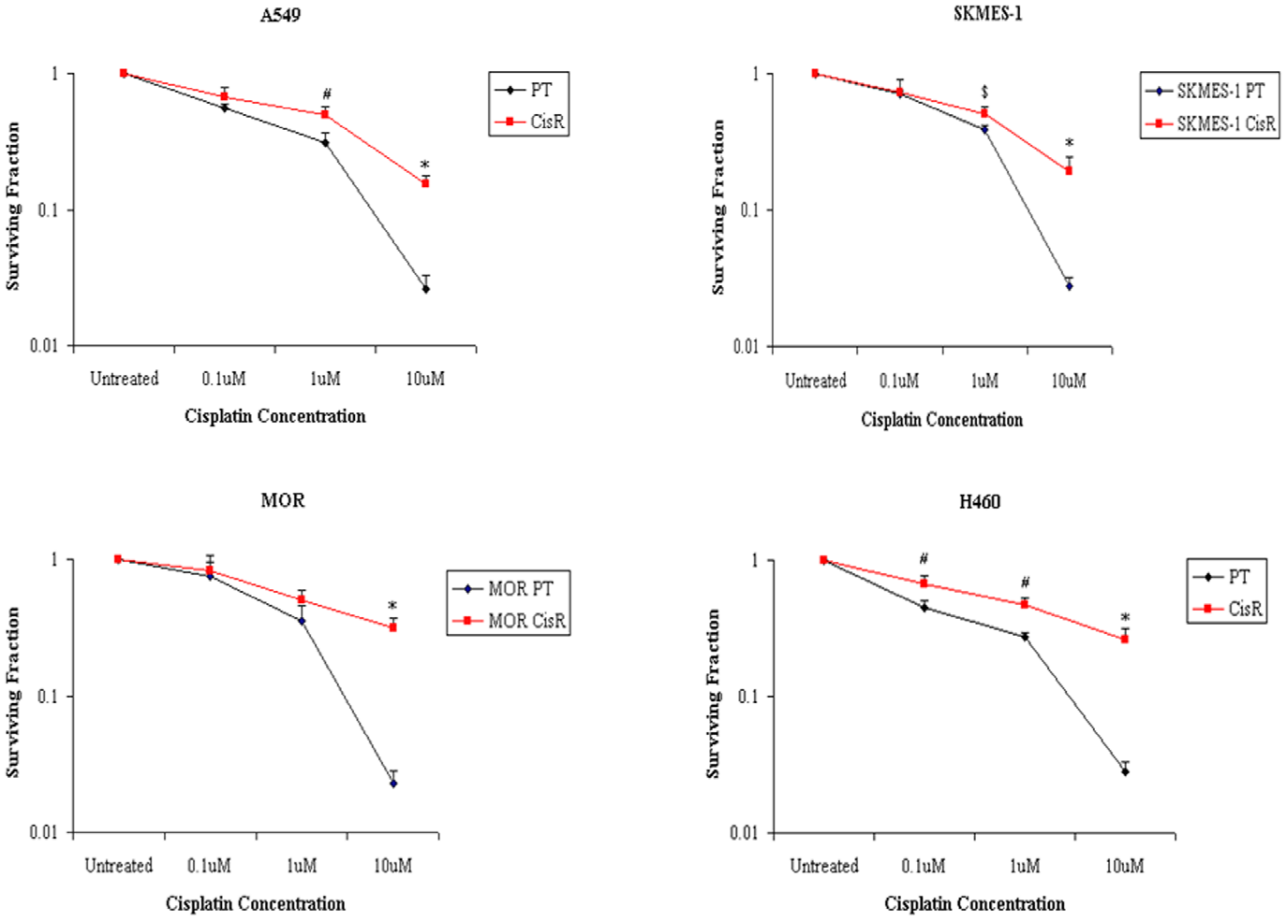
Clonogenic survival ability of cisplatin resistant cells is increased with increasing doses of cisplatin. A549, SKMES-1, MOR and H460 PT and CisR cells were seeded in 6-well plates using optimised seeding densities. Following treatment with cisplatin for 72 h, media was removed and cells were allowed to recover for between 9–14 days after which time surviving colonies were stained using crystal violet stain and counted. The survival ability of CisR cells was significantly increased at various concentrations of cisplatin between cell lines relative to their parental counterparts, based on the number of colonies on plate following incubation with cisplatin. Data are expressed as Mean ± SEM from three independent experiments (n = 3) (^$^p<0.05, ^#^p<0.01, ^*^p<0.001).

The survival ability of PT and CisR NSCLC cells following treatment with cisplatin was assessed using the clonogenic survival assay. All cell lines showed variable resistance between PT and CisR cells. In the majority of cell lines examined, there was a significantly higher fraction of surviving colonies of A549, SKMES-1 and H460 CisR cells relative to parent cells at 1 µM and 10 µM of cisplatin (Fig. 6). The H460 cell line demonstrated greater resistance however, with a surviving fraction of 0.67±0.04, 0.47±0.03 and 0.26±0.02, at 0.1 µM, 1 µM and 10 µM, respectively, relative to the parent cell line (0.45±0.06, 0.27±0.02 and 0.03±0.01). While MOR CisR cells showed a significant survival of colonies relative to parent cells at 10 µM (0.32±0.03 vs 0.02±0.01), this was not significant at 1 µM (0.45±0.05 vs 0.37±0.03). These clonogenic survival data further confirm the cisplatin resistant phenotype of A549, SKMES-1, MOR and H460 sublines derived from each parent cell line.

### Cisplatin-resistant Cell Lines Exhibit Enriched Fractions of CD133+CD44+ Cells

**Figure 7:**
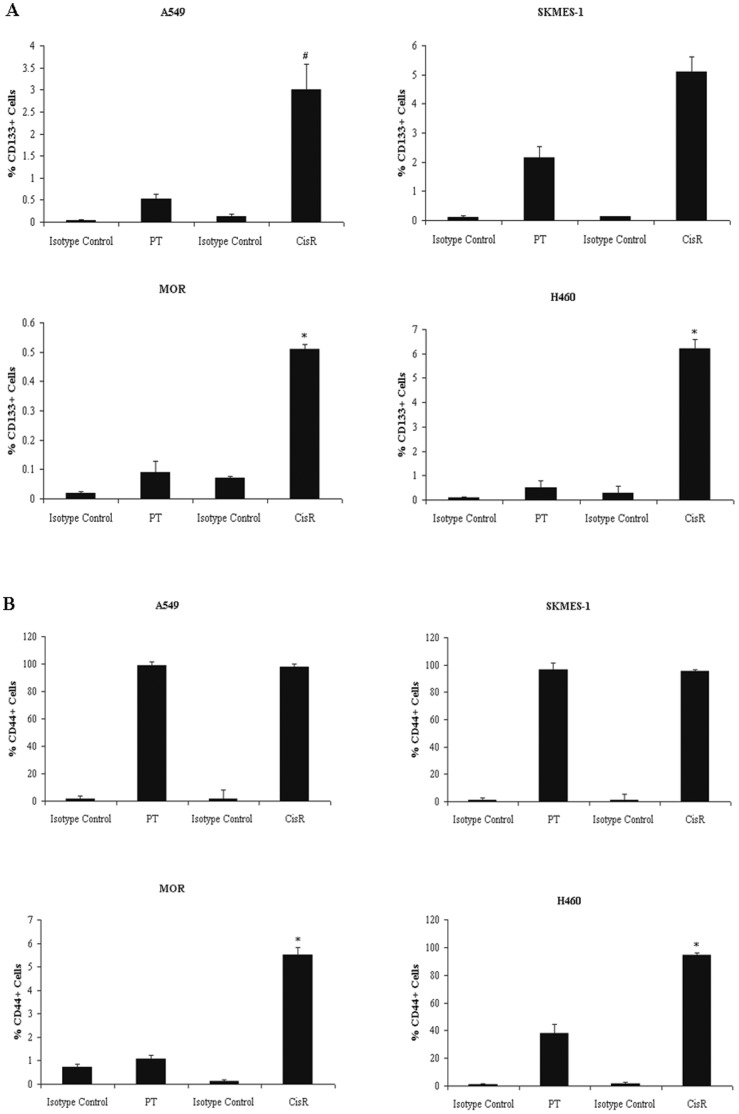
Enrichment of CD133+ and CD44+ fractions in cisplatin resistant sublines. Antibody staining of PT and CisR cell lines for CD133 cell surface expression was carried out by flow cytometry using a CD133/1 (AC133) phycoerythrin (PE)-labelled antibody and IgG1 isotype control antibody. The percentage CD133+ cells were plotted for all cell lines (A). Differential expression of the CSC marker CD44 was examined using an anti-human CD44 FITC-conjugated antibody and corresponding IgG2b isotype control antibody. Expression levels of CD44 were determined for all cell lines and plotted as a percentage of the tumour cell population expressing CD44 (B). Data are expressed as Mean ± SEM from three independent experiments (n = 3) Data are expressed as Mean ± SEM from three independent experiments (n = 3) (^#^p<0.01, ^*^p<0.001).

Because the cancer stem-cell compartment comprises of a very small fraction of the total cancer cell population, it is necessary to utilise specific cell surface markers for cancer stem cells. The expression profile of putative stem cell surface markers, CD133 and CD44, were examined between parent and corresponding cisplatin resistant cell lines. Using double staining by flow cytometry, we examined whether the established cisplatin resistant cell lines displayed an enrichment of CD133+ cells and whether cells expressing CD133 were also CD44 positive. A549, MOR and H460 cell lines exposed to cisplatin showed an increased enrichment of CD133+ cells relative to each matched parent cell line (Fig. 7A). A 5-fold enrichment in the CD133+ population in CisR A549 (2.89±0.58 vs 0.53±0.11) and MOR (0.51±0.02 vs 0.09±0.04) cells was observed compared to parent cells, while a greater than 12-fold increase was observed in H460 CisR cells (6.20±0.40 vs 0.50±0.30). The cell surface marker, CD44, was also expressed on each of these cell lines (Fig. 7B). Levels of CD44 were similar in A549 PT and CisR cells, while a significant increase in expression of CD44+ cells was observed MOR CisR cells relative to PT cells (5.52±0.31 vs 1.09±0.15). Similarly, a significant increase was detected in H460 CisR cells (94.42±0.52 vs 38.22±6.08), with much greater levels of CD44+ cells also seen in MOR CisR cells relative to parent cells (5.525±0.305 vs 1.085±0.145). SKMES-1 cells contained a greater than 2-fold enrichment of CD133+ cells within the cisplatin resistant population relative to the parent cell line (5.10±1.27 vs 2.16±0.69). Both PT and CisR SKMES-1 cell lines had similar levels of CD44, similar to that observed in A549 cells, demonstrating a cell progeny with the same CD44 expression profile. For CD133 and CD44 markers, there was no statistically significance between PT and CisR SKMES-1 cells. Interestingly however, all cisplatin-resistant NSCLC cell lines with increased CD133+ fractions also exhibited increased numbers of CD44+ cells (CD133+/CD44+).

### Identification of Increased ALDH Activity in Cisplatin Resistant Cell Lines

**Figure 8:**
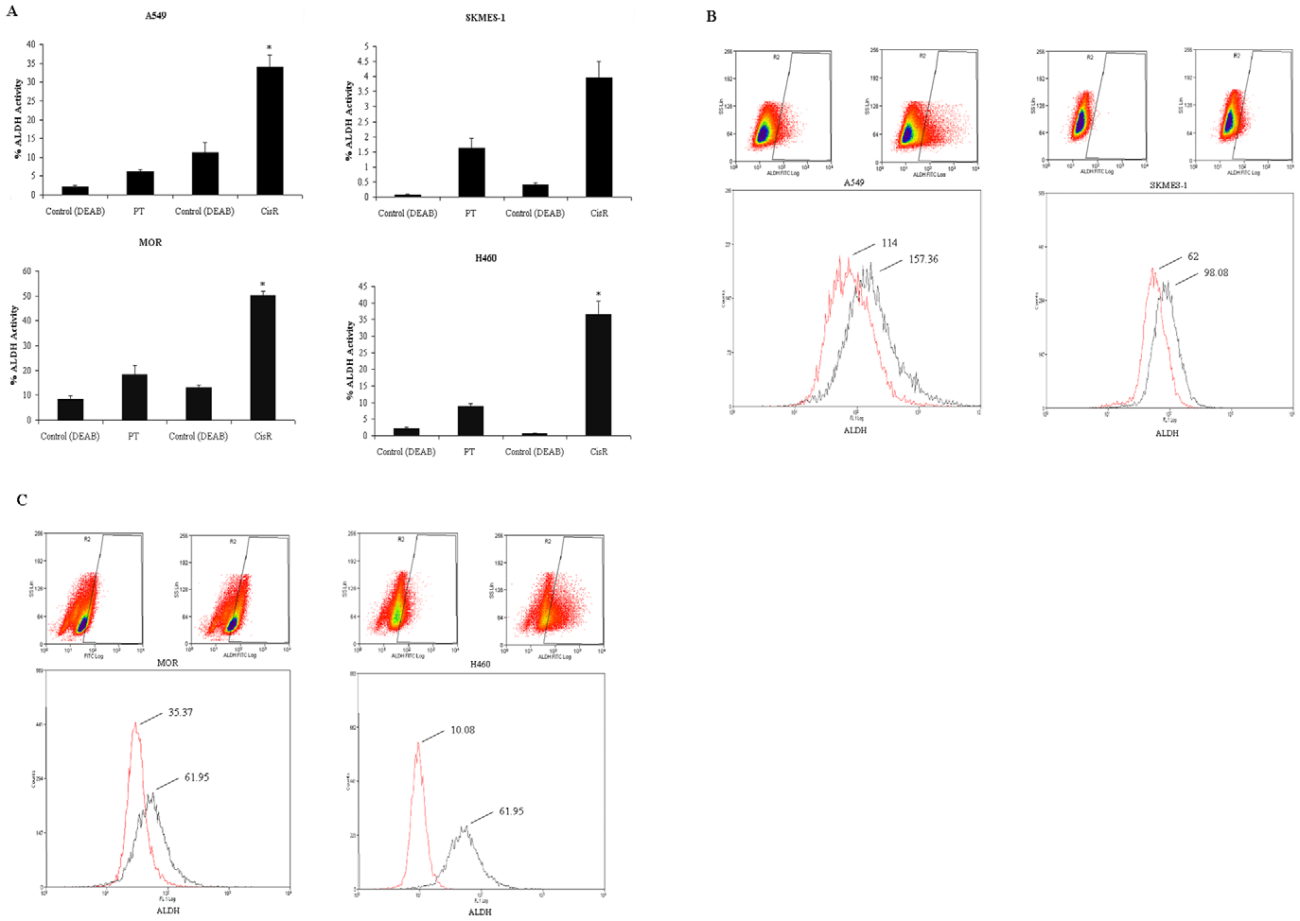
Chemoresistant cell lines display increased aldehyde dehydrogenase (ALDH1) activity. ALDH1 activity was measured between PT and CisR NSCLC cells using the Aldefluor assay. Cells were incubated with ALDH1 substrate that converts intracellular ALDH1 into a negatively charged reaction product, preventing diffusion from the cells. A control sample was also included for each parent and resistant cell line that consisted of a specific inhibitor of ALDH1, diethylaminobenzaldehyde (DEAB), in order to establish baseline fluorescence. The reaction was measured in the green fluorescence channel of a flow cytometer. The percentage ALDH1 activity between parent and cisplatin resistant cells was calculated (A). Dot plots and histograms showing mean intensity fluorescence (MFI) between parent (red) and cisplatin resistant (black) cell lines are represented (B). Data are expressed as Mean ± SEM from three independent experiments (n = 3) (^*^p<0.001).

Using the Aldefluor assay to assess the presence and size of the cell population with ALDH enzymatic activity in our panel of four NSCLC cell lines, a significant increase in ALDH activity was demonstrated within each population of CisR cells relative to parent cells (Fig. 8A) as illustrated by representative dot plots and mean fluorescence intensity histograms (Fig. 8B). Relative to PT cells, A549, MOR and H460 CisR cells had significantly increased levels of ALDH+ cells (*A549*, 34.04±3.10 vs 6.08±0.60, *MOR*, 50.24±1.63 vs 18.40±3.79, *H460*, 36.39±2.34 vs 8.89±0.75). However, while there was a trend towards an increase in the ALDH+ fraction in the SKMES-1 CisR cell line, this was not significant relative to the parental cell line (3.96±1.16 vs 1.62±0.32). This pattern of expression was similar to that observed for CD133 in SKMES-1 CisR cells, where only a modest increase in CD133+ cells was also found.

### Cancer Stem Cell Marker Expression Profile of Nanog, Oct-4 and SOX-2 Proteins

**Figure 9:**
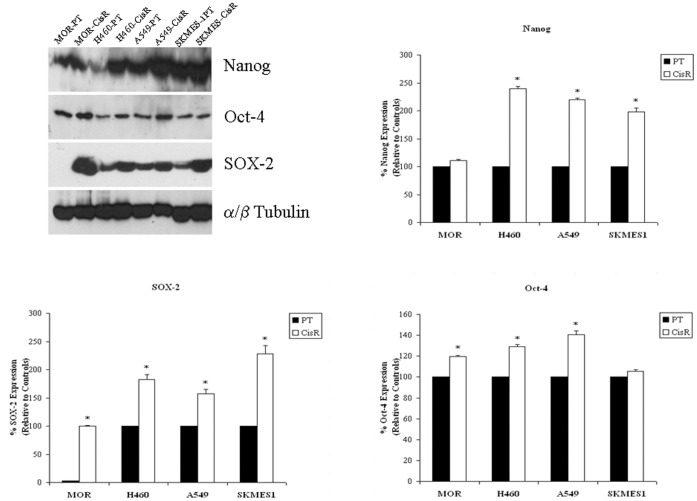
Cancer stem cell markers, Nanog, Oct-4 and SOX-2, are upregulated in cisplatin resistant cells. Total proteins were isolated from parent and corresponding cisplatin resistant sublines and subjected to SDS-PAGE gel electrophoresis and transfer by Western blot. Using a panel of human embryonic stem cell markers, Nanog, Oct-4 and SOX-2 protein expression was examined between parent and resistant cell lines. While H460, A549 and SKMES-1 resistant cells exhibited increased expression of Nanog and SOX-2 proteins, Oct-4 expression was significantly upregulated in MOR, H460 and A549 cells only. Cisplatin resistant MOR cells demonstrated increased levels of SOX-2 and Oct-4 proteins relative to parental cells. Data are expressed as Mean ± SEM from three independent experiments (n = 3) (^*^p<0.001).

In order to further define a distinct cisplatin resistant stem cell population of NSCLC cells within our panel of cell lines, a number of key human embryonic cancer stem cell markers were examined at the protein level between parent and cisplatin resistant cell lines. Three distinct cancer stem cell markers, Nanog, Oct-4 and SOX-2 were assessed (Fig. 9). Differential expression was observed across all cell lines. Significantly increased expression of Nanog was observed in H460 (239.67±4.055), A549 (220±2.517) and SKMES1 (198±7.00) cisplatin resistant cell lines compared to controls or parent cells (100%). Little, if no difference, was observed in MOR cisplatin resistant cells relative to parental cells. Protein levels of Oct-4 were significantly upregulated in MOR (120±0.8819), H460 (129±2.082) and A549 (140.66±2.963) cell lines but not in SKMES1 (105.33±1.453) cells. Significant increases in the levels of SOX-2 protein expression were observed across all cisplatin resistant cell lines relative to parent cells.

### Resistant Cells Demonstrate Increased Expression of the EMT Regulators, c-Met and β-catenin

**Figure 10:**
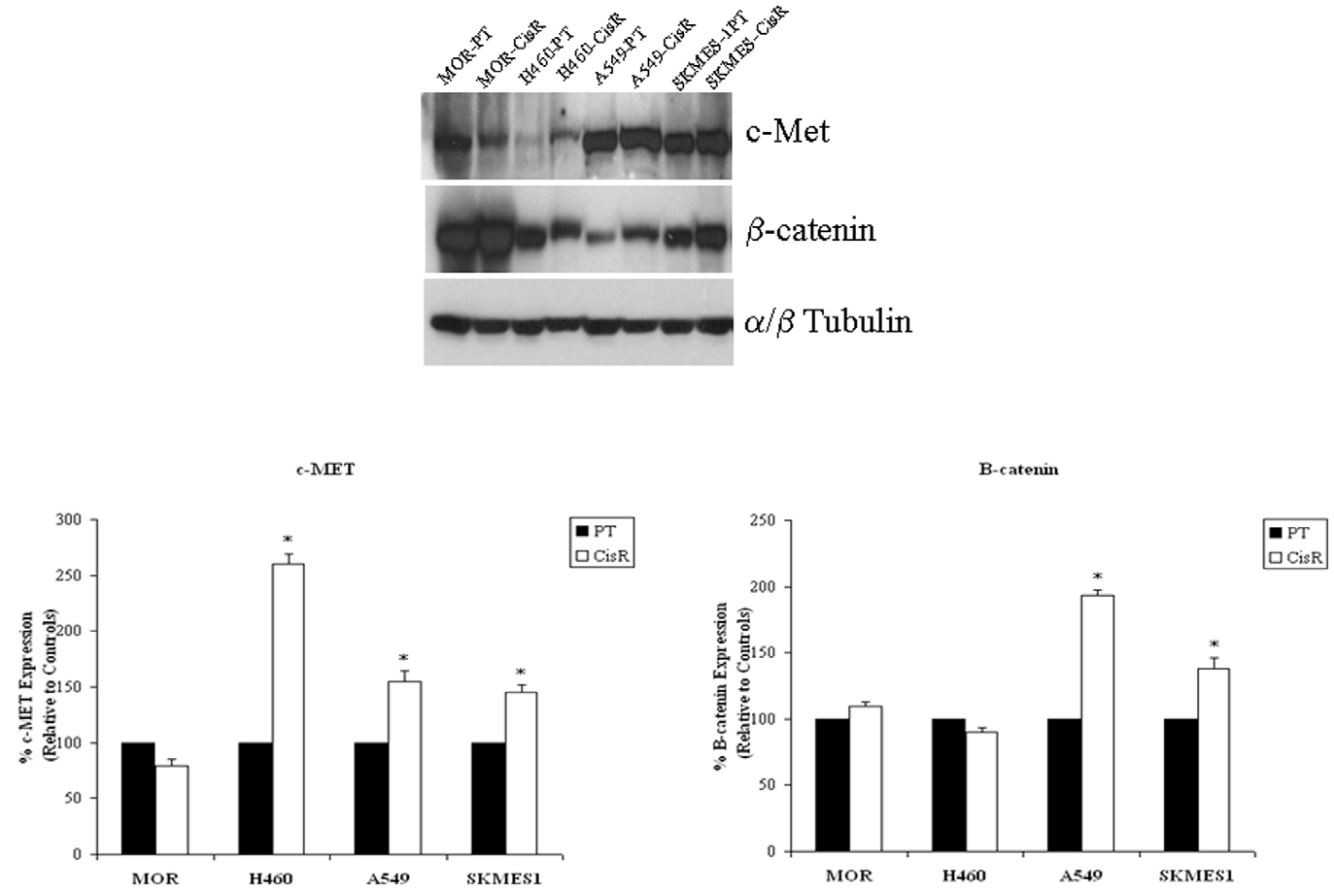
EMT marker expression, c-Met and β-catenin. Total proteins from parent and corresponding cisplatin resistant sublines were subjected to SDS-PAGE gel electrophoresis and transfer by Western blot. Expression levels of the EMT markers, c-Met and β-catenin, were examined across all cell lines. c-Met protein levels were significantly upregulated in H460, A549 and SKMES-1 resistant cell lines, while β-catenin levels were significantly upregulated in A549 and SKMES-1 cells only. Data are expressed as Mean ± SEM from three independent experiments (n = 3) (^*^p<0.001).

The epithelial to mesenchymal transition (EMT) is a key step in the progression of tumours towards metastasis and invasion. Moreover, cancer cells undergoing EMT have been found to show increased resistance to apoptosis and certain chemotherapeutic drugs and acquire traits reminiscent of those expressed by stem cells. In a preliminary analysis, expression levels of two important EMT regulators, c-Met and β-catenin, were examined at the protein level in our panel of cisplatin resistant and parent cell lines (Fig. 10). While H460 (260.62±8.426), A549 (155.25±9.357) and SKMES1 (145.62±6.741) cisplatin resistant cell lines showed significantly higher levels of c-Met protein compared to that observed in their parent counterparts (100%), β-catenin was significantly upregulated in A549 (193.33±4.269) and SKMES1 (138.27±7.679) cells only.

### Cisplatin Resistant Lung Cancer Cells Show Decreased Cisplatin-GpG DNA Adduct Formation

**Figure 11:**
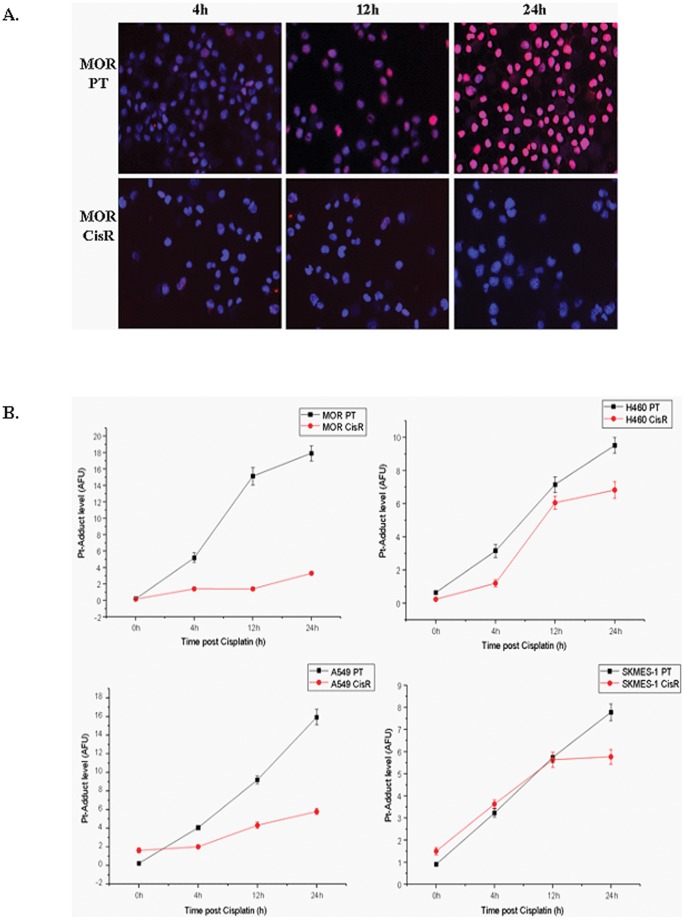
Cisplatin-DNA adduct formation and immunofluorescence. Lung cancer cell lines were treated with cisplatin for up to 24 h and fixed on Superfrost Gold Slides using ice-cold methanol. Cells were stained overnight at 4°C using a primary antibody that specifically recognizes CDDP-GpG DNA adducts (RC-18). Antibody binding was detected using an anti-rat Cy3®-labelled antibody and counterstained using DAPI (1 µg/ml (w/v). Images were acquired on an Axioplan fluorescence microscope (A). Adducts were quantified and measured as arbitrary fluorescence units (AFU’s) upon normalisation of integrated antibody-derived fluorescence from 200 individual nuclei of the corresponding DNA content. Data are presented as the mean AFU ±95% confidence interval (CI) from three independent experiments (B).

To determine the level of DNA adducts in the nuclear DNA of cisplatin resistant cells, we established a quantitative immunocytological assay using a monoclonal antibody-based immunocytological measurement of DNA intrastrand cross-links. Following treatment of parent and cisplatin resistant NSCLC cells with cisplatin over a time-course of 24 h, cells were stained for the quantitative analysis of Pt-(GpG) cross-links in DNA using a specific antibody RC-18. In our panel of parental cell lines, an increase in cispatin-DNA adduct formation was readily detectable in the nuclei. This was in contrast to that observed in corresponding cisplatin resistant cells where there was significantly less adduct formation in all resistant cell lines, most notably in MOR cells (Fig. 11A). The measurements of integrated immunofluorescence signals from individual nuclei by quantitative image analysis revealed a distinct pattern of adduct levels between each parent and cisplatin resistant cell line. The accumulation of Pt-DNA lesions 24 h post treatment was significantly higher in all resistant cell lines (Fig. 11B).

### Enhanced DNA Double-strand Break Repair Ability of Resistant Sublines

**Figure 12:**
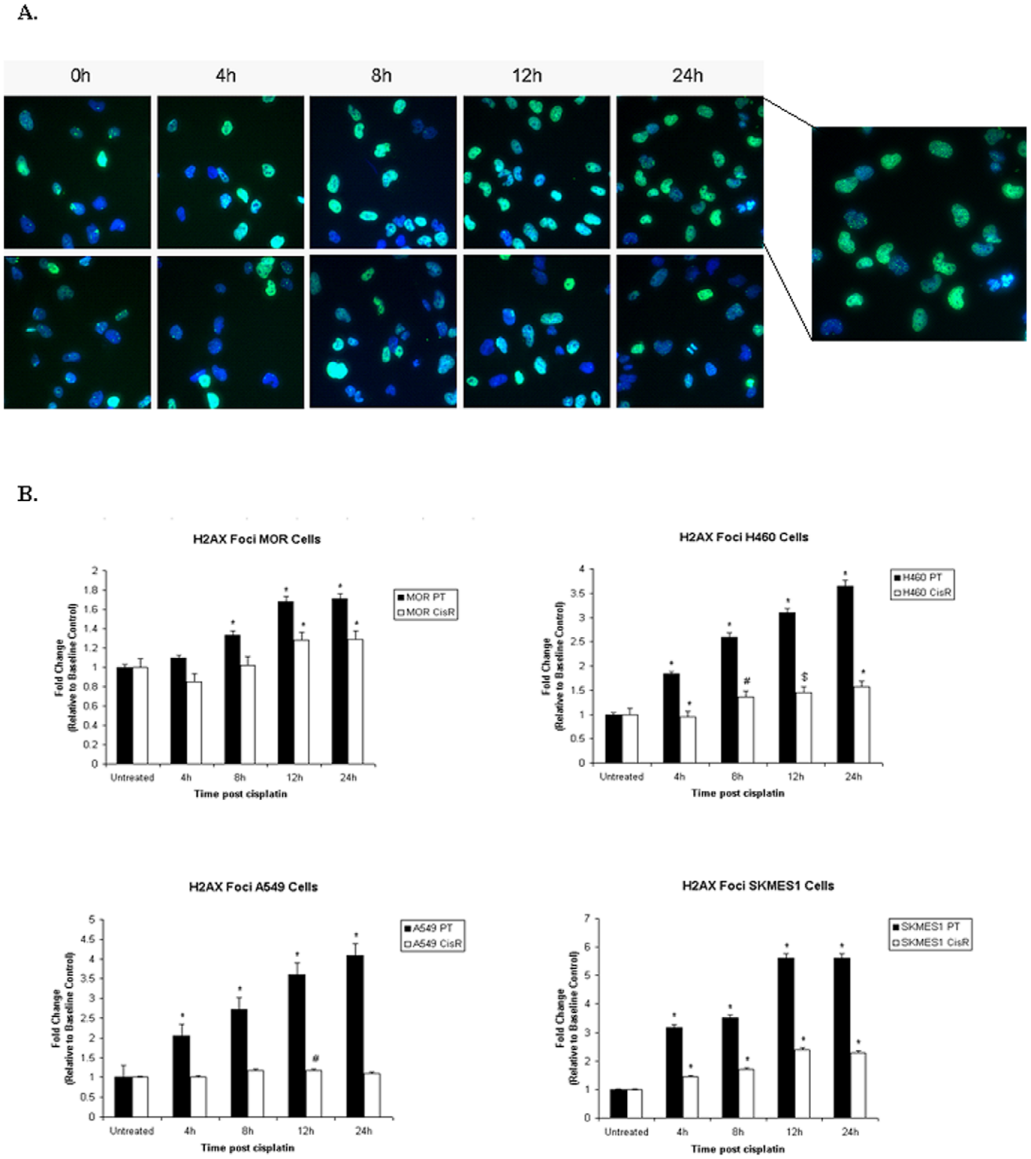
Measurement of γH2AX foci formation and DNA damage. Following treatment of parent and chemoresistant cells with cisplatin for 4, 8, 12 and 24 h, cells were fixed in formaldehyde and incubated with a primary rabbit anti-human anti-phospho-histone 2AX (Ser139) antibody. Cells were subsequently labelled with an Alexafluor 488-labelled goat anti-rabbit secondary antibody and Hoechst 33342 nuclear stain prior to analysis by high content analysis using the InCell Analyser 1000 (A). Data are expressed as Mean ± SEM from three independent experiments (n = 3) (^#^p<0.05, ^$^p<0.01, ^*^p<0.001) (B).

To investigate DNA double strand break (DSB) repair capacity in our panel of cell lines, the H2AX foci formation assay was used following treatment of parent and cisplatin resistant sublines over a period of 24 h. At 4, 8, 12 and 24 h post treatment, resistant cells repaired DNA-DSB’s more efficiently than parent cells, as indicated by the significantly lower amount of phosphorylated-γH2AX foci (Fig. 12A). While exposure of parental cells to cisplatin resulted in a gradual accumulation of γ-H2AX foci with significant increases as early as 4 h post cisplatin treatment, this effect was most pronounced by 24 h. In contrast, the number of foci was significantly lower in resistant sublines following treatment with cisplatin (*MOR* 68.509±1.72 vs 82.645±0.73, p<0.01; *H460* 47.81±0.65 vs 74.48±1.62, p<0.001; *A549* 39.40±1.26 vs 69.11±0.93, p<0.001; *SKMES-1* 72.09±0.98 vs 98.66±1.52, p<0.001) suggesting a increase in the DNA repair capacity of these chemoresistant cell lines. Within each cell line, differences in foci number varied at each time point. In A549 cisplatin resistant cells, little difference in phosphorylated-H2AX between time points was observed, with an significant increase at 12 h post treatment only.

### Cellular Uptake of Cisplatin is Reduced in Chemoresistant Cells

**Figure 13:**
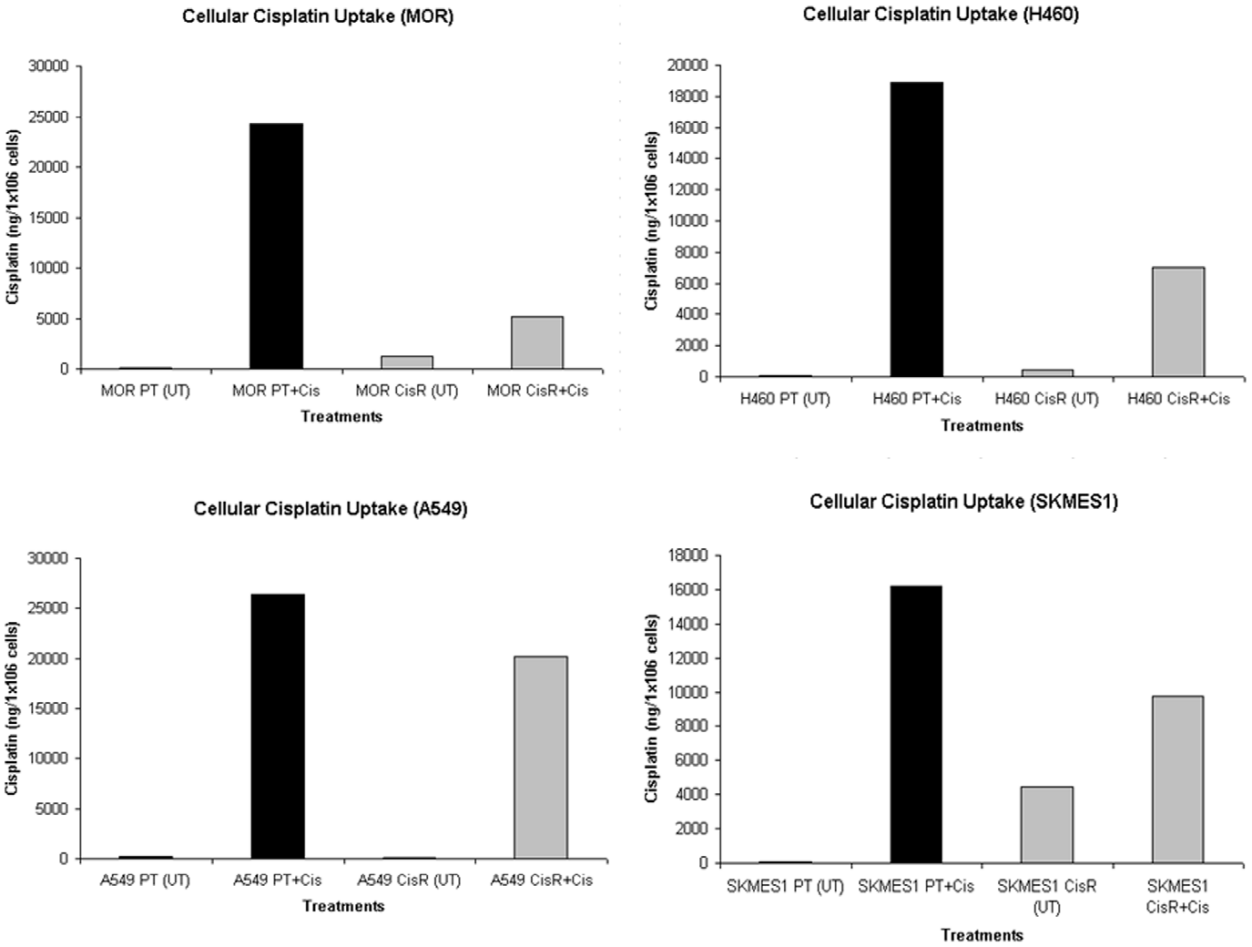
Quantification of cisplatin uptake by lung cancer cells using ICP-MS. Exponentially growing cells were treated with cisplatin for 24 h after which time they were washed in PBS, harvested and counted. Digestion of cells (1×10^6^) in 1% nitric acid for 24 h at 70°C was carried out prior to ICP-MS analysis. Platinum determination was performed using Inductively Coupled Plasma Mass Spectrophotometry. Instrumental settings were optimised in order to yield maximum sensitivity for platinum.

To determine differences in sensitivity to the growth inhibitory effects of cisplatin between parent and resistant lung tumour cells were accompanied by differences in whole cell platinum accumulation, as is commonly observed in cells selected for such platinum resistance, our panel of cell lines were treated with cisplatin for 24 h and intracellular cisplatin levels were quantified by ICP-MS (Fig. 13). Across all four parental cell lines, findings from ICP-MS analysis demonstrated a increased uptake of cisplatin after treatment for 24 h relative to untreated parental cells (*MOR* 24,319.20 ng vs 98.45 ng; *H460* 18,890 ng vs 74.57 ng; *A549* 26,417.60 ng vs 183.63 ng; *SKMES-1* 16,184.90 ng vs 105.10 ng) Resistant cells however, had significantly reduced uptake of cisplatin following exposure to cisplatin for a similar time period, compared to that observed in their parental counterparts (*MOR* 5,217.8 ng vs 23,319.20 ng; *H460* 6,984.30 ng vs 18,890 ng; *A549* 20,204 ng vs 26,417.60 ng; *SKMES-1* 9,781.80 ng vs 16,184.90 ng).

## Discussion

Since its introduction into clinical trials, cisplatin has had a major impact in the treatment of cancer, changing the course of treatment for several tumours such as those of the ovary, testes, head, neck and lung (2). To date, the most effective systemic chemotherapy for NSCLC is platinum-based combinations that remain the standard first-line chemotherapy for this cancer type. While an understanding of the mode of action is desirable in refining therapeutic approaches that further enhance the anti-tumour activity of this platinum drug, cisplatin poses a number of major problems, one of which is the acquisition of cisplatin resistance that undermines its curative potential. This understanding is also critical for elucidating mechanisms underlying the drug-resistant phenotype associated with cisplatin resistance, particularly in NSCLC. An example highlighting this limitation is with ovarian cancer which generally responds well to cisplatin-based therapy. Unfortunately, the initial response rate of up to 70% is not durable and results in a 5-year patient survival rate of only 15–20%, primarily as tumours become resistant to therapy [14]. Likewise, in small cell lung cancer, the relapse rate can be as high as 95% [15]. The onset of resistance creates a further therapeutic complication in that tumours failing to respond to cisplatin are cross-resistant to diverse unrelated anti-tumour drugs [16]. This suggests that cisplatin and other agents likely share common mechanisms of resistance. In this respect, it is noteworthy that cisplatin-resistant tumours are fully cross-resistant to the platinum analogue carboplatin [17], [18]. As cisplatin-based chemotherapy for NSCLC appears to have reached a plateau and a better understanding of the mechanisms of cisplatin resistance are slowly unravelling, there is an urgent need for a better understanding of the molecular mechanisms underlying the cisplatin resistant phenotype.

We have generated a clinically-relevant, isogenic model of cisplatin resistance in a panel of NSCLC cell lines from original, age-matched parent cell lines and characterised these in terms of their proliferative and apoptotic potential, cell cycle distribution, clonogenic survival ability and stem-like properties. Using IC_50_ concentrations, cisplatin resistant cell lines were established over time through chronic *in vitro* exposure to the drug after which time IC_50_ values were re-assessed in cisplatin treated cell lines and found to be significantly higher, demonstrating a more resistant phenotype. Changes in the proliferative and apoptotic properties of cisplatin resistant cell lines relative to their corresponding parent cell lines in response to increasing concentrations of cisplatin suggested increased resistance to the chemotherapeutic drug with significant differences observed between parent and resistant cells at various concentrations. A549 and H460 cell lines, in particular, were found to be most resistant to cisplatin in terms of their proliferative and apoptotic response to cisplatin. Differences in the cell cycle distribution of PT and CisR cell lines, at basal levels, were also observed, with cisplatin resistant cells having a higher accumulation of cells in the G0/G1 phase of the cell cycle and a corresponding decrease in the number of cells in S phase. This difference was most notable in A549 and H460 cells.

One of the most common cellular checkpoints affected in response to cisplatin treatment is G2/M arrest where p21^WAF1^, one of several genes transactivated by p53 as a result of exposure to cisplatin, is involved in both inducing and sustaining this cell cycle arrest [19]. However, the accumulation of p21^WAF1^ following DNA damage has been classically associated with a G0/G1 arrest [20]. It is of interest that A549 and H460 cell lines were most resistant of the four cell lines characterised, while the squamous cell carcinoma cell line, SKMES-1, was the least resistant of these. Possible molecular factors that may influence, in part, the resistance phenotype observed between our panel of cell lines may be attributable to their p53 status. While A549, MOR and H460 cells have wild-type p53, SKMES-1 cells are p53 mutant. Activation of p53 by cisplatin-induced DNA damage has been reported to have various effects on cellular sensitivity to cisplatin. In some studies, activation of p53 has been shown to provide cytoprotection against cisplatin [21], [22] In contrast, increased resistance to cisplatin with disruption of normal WT p53 function has also been demonstrated [23].

Since the discovery of cancer stem cells in haematopoietic cancers and other solid tumours, little is known to date regarding the biology of lung cancer stem cells. The existence of cancer stem cells within a lung tumour cell population may explain the ineffectiveness of current treatments in consistently eradicating tumour cells. Therapies may target the majority of cancer cells while residual lung cancer stem cells may regenerate a cancer cell population resulting in tumour relapse following chemotherapy. As such, there is an increasing need to identify and develop new therapeutic targets for specifically eradicating this cell population. While the marker profile of lung cancer stem cells remains to be explored, some commonly used strategies that have been used to date include the cell surface stem cell markers, CD133 and CD44, in addition to aldehyde dehydrogenase activity. Recent studies using NSCLC cell lines and fresh lung tumour tissues suggest CD133 as the lung CSC marker of choice [7], [24], [25], [26] while cytometric analysis and sorting of marker-positive cells is currently the standard method used [27]. In a recent study by Bertolini *et al*., cisplatin treatment of lung cancer cells resulted in the enrichment of a CD133+ fraction of cells with a cisplatin resistant phenotype following acute cytotoxic exposure to cisplatin. Likewise, *in vivo* subpopulations of CD133+ cells were spared by cisplatin treatment of lung tumour xenografts established from primary lung tumours. Exposure of A549 lung tumour cells to cisplatin using IC_80_ concentrations resulted in an 8-fold enrichment of CD133+ cells. In support of these findings, cisplatin resistant A549 cells generated in our study, showed a greater than 5-fold increase in CD133+ expressing cells (IC_50_ concentration) relative to parent cells, highlighting and further confirming CD133 as a potential marker of cisplatin resistance in NSCLC. Biochemical studies demonstrating a functional role for CD133 in cell cycle regulation and proliferation have been reported [28], consistent with some of the functional studies highlighted in the cisplatin resistant NSCLC cell lines established in this study. Chemoresistant cells expressing increased levels of CD133 also showed a significant arrest in the G0/G1 phase of the cell cycle relative to parent cells.

The membrane-bound glycoprotein, CD44, is found expressed in many tumour cell types and is an important factor in tumour growth, invasion and metastasis. Recent studies have provided support for its role as CSC marker. In colorectal cancer, the clonal expansion and xenograft initiation capacity of CD44+ CSCs could be inhibited by CD44 knockdown [29]. In small cell lung cancers, it was shown that activation of CD44-MAPK-PI3K signalling results in the increased expression of urokinase plasminogen activator and its receptor, uPAR, and MDR1, resulting in enhanced invasive and multi-drug resistant cancer phenotypes [30]. In our panel of cisplatin-resistant NSCLC cell lines, an enrichment of CD44-expressing subpopulations was demonstrated. Such findings are in agreement with recent studies examining the identification of lung CSCs in a series of *in vitro* and *in vivo* studies [31]. However, differences in CSC marker profile expression do exist between studies. In the study reported by Leung *et al*., 0% and 95.90% of CD44+ cells was observed in A549 and H23 cell lines, respectively, while in a study by Stuelten *et al*. [32], 84.41% and 30.95% were detected. Our findings using A549 cells are in agreement with those of Stuelten *et al*. where 97.69% CD44+ cells were found within the cisplatin-resistant population and 98.71% in parent cells. Such variations in expression between studies may be explained by individual variation among different cell lines or differences in the composition or functional characteristics of the cancer stem cell populations. Determining the true percentage of CSC’s within tumours or established cell lines remains controversial in the absence of a specific CSC marker, particularly in lung cancer.

The aldehyde dehydrogenase family of enzymes belong to a family of intracellular enzymes involved in cellular detoxification and oxidisation of intracellular aldehydes, resulting in drug resistance [33], [34]. Its function and clinical significance in relation to stem cell function is still under investigation in lung cancer. There is however, documented evidence to support ALDH as a marker for lung cancer stem cells. In a study by Jiang *et al*., high levels of ALDH protein expression correlated with poor prognosis, consistent with the idea that ALDH+ lung tumour cells are enriched with lung cancer stem cells [35]. It is of interest that in our panel of cisplatin resistant NSCLC cell lines that displayed a significant increase in the number of CD133+ cells, there was a significant corresponding enrichment of the cancer stem cell marker, CD133, relative to that seen in parent cells. This was significantly increased in A549, MOR and H460 cells, with the exception of the squamous cell carcinoma cell line, SKMES-1. Such findings are in agreement with a more resistant cell phenotype. The histological and regional diversity found in lung cancer may, in part, be attributed to the presence of diverse pools of self-renewing stem cells in the adult lung epithelium [36].

Evidence that cisplatin resistant subpopulations of cells within our panel of cell lines display characteristics of putative cancer stem cells is further supported in this study using a panel of cancer stem cell markers which were differentially upregulated across our panel of cell lines. Nanog, Oct-4 and SOX-2 stem cell markers were significantly upregulated in a number of cisplatin resistant cell lines compared to their corresponding parental counterparts. However, while such increases in expression of Nanog, Oct-4 and SOX-2 represent a pluripotency regulation network, significantly elevated levels of SOX-2 protein were found compared to that found Nanog and Oct-4. Recent studies demonstrate that CSC’s have higher tumorigenic properties than those of differentiated cancer cells and that the transcription factor, SOX-2, plays a vital role in maintaining the unique properties of CSC’s [37]. However, the function and underlying mechanism of SOX-2 in carcinogenesis of lung cancer are still elusive. In a study by Chen *et al*, expression of SOX-2 in human lung tissues of normal individuals as well as patients with adenocarcinoma, squamous cell carcinoma, and large cell carcinoma demonstrated specific overexpression of SOX-2 in all types of lung cancer tissues. This finding supports the notion that SOX-2 contributes to the tumorigenesis of lung cancer. In addition, higher expression of the oncogenes *c-MYC*, *WNT1*, *WNT2* and *NOTCH1* was detected in side population (SP) cells than in non-side population (NSP) cells of A549 lung cancer cells, indicating a possible mechanism for the tumorigenic potential of CSC’s. Silencing of the SOX-2 gene reduced the tumorigenic properties of A549 cells with subsequent attenuated expression of *c-MYC*, *WNT1*, *WNT2*, and *NOTCH1* in xenografted NOD/SCID mice. These results provide evidence that SOX-2 may regulate the expression of oncogenes in CSC’s to promote the development of human lung cancer [38].

The progression of many cancer types is often accompanied by changes in the pattern of gene expression of neoplastic cells, resulting in a highly tumorigenic and invasive cell phenotype. Some of these changes are reminiscent of an epithelial to mesenchymal transition (EMT), a process characterised by loss of epithelial features and gain of mesenchymal properties. While loss of E-cadherin has emerged as one of the common indicators of EMT, this has been shown to result in the release of β-catenin in addition to its cytoplasmic accumulation and further translocation to the nucleus where it can activate LEF/TCF (lymphoid enhancer factor/T cell factor) transcription. We show in this preliminary analysis of EMT marker expression that β-catenin in significantly upregulated in two of our cisplatin resistant cell lines. Dysregulation of the c-Met receptor, or overexpression of its ligand, hepatocyte growth factor (HGF), has also been associated with an aggressive cancer cell phenotype and the EMT process. Our data highlight the potential involvement of this EMT regulator in NSCLC cells with a cisplatin resistant phenotype with increased protein expression of c-Met in three of four resistant sublines. Further studies using inhibitors to EMT signalling pathways may be warranted to circumvent the resistance conferred by certain cancer cells to chemotherapeutic agents.

The anti-cancer activity of cisplatin is based on the formation of platination products in the nuclear DNA [39]. Several of these adducts have been identified, of which the guanine-guanine intrastrand cross-link, cis-Pt(NH_3_)_2_d(pGpG) [Pt-(GG)], represents >70% of total DNA platination. Persistence of such lesions within the nuclear DNA can ultimately result in impaired replication and transcription, thereby triggering apoptosis. The nucleotide excision repair (NER) pathway has been suggested to be one of the main cellular defense mechanisms against cisplatin-induced intrastrand cross-links [40]. Up until recently, the measurement of platinum concentrations was based predominantly on spectroscopic methods [41]. In this study, we used an adduct-specific monoclonal antibody in combination with digital image analysis to visualise and quantify levels of distinct DNA platination products within the nuclei of individual cells. The degree of DNA adduct formation by cisplatin is cell-type specific and may likely depend on a number of pharmacokinetic parameters such as drug export by membrane transporters [42] or cytoplasmic detoxification [43].

We investigated the effects of cisplatin on the repair of cisplatin-induced double strand breaks (DSB’s) by immunofluorescence imaging of γH2AX foci. Given that γH2AX appears rapidly at DSB’s and disappears as repair proceeds [44], it serves as a sensitive and specific marker for unrepaired DNA damage. These findings, together with our observation that chemoresistant cells displayed decreased cisplatin-GpG DNA adducts following exposure to cisplatin compared to parent cells, are indicative of potential key mechanisms that may be implicated in the process of cisplatin transport and/or repair in our panel of NSCLC cell lines. Data from ICP-MS analysis demonstrated a significant accumulation of cisplatin in parent cells upon treatment with cisplatin compared to that measured in cisplatin resistant cells. Upon treatment, platinum drugs have been shown to be extensively sequestered into subcellular compartments which in turn limit their access to critical targets. While in some cell types, this sequestration process is accompanied by enhanced drug export [45], others have shown enhanced storage of the drug inside the cell, most likely in a non-toxic form [46]. In the latter of these studies, forced expression of the copper transporters ATP7A and ATP7B rendered cells resistant to cisplatin and other platinum drugs. Future studies warrant investigation as to the expression of these copper transporters in cisplatin resistant lung cancer cells and to verify whether this resistance mechanism is independent of copper efflux transporters.

We have generated an isogenic model of cisplatin resistance in a panel of NSCLC cell lines and characterised these based on a number of functional cellular parameters relative to their original parental cell line. The presence and enrichment of stem-cell markers support the presence of a chemoresistant population of lung cancer cells with a stem-like signature that may be useful as a clinically relevant *in vitro* model for studying mechanisms of cisplatin resistance in NSCLC. Moreover, we have identified differences in cisplatin-DNA adduct formation and DNA repair of cisplatin-induced DSB’s between parent and chemoresistant cells following uptake of cisplatin. These findings provide a rationale for more specific therapeutic targeting in the treatment of this disease.
