# Correction: Generation and Characterisation of Cisplatin-Resistant Non-Small Cell Lung Cancer Cell Lines Displaying a Stem-Like Signature

**DOI:** 10.1371/journal.pone.0233739

**Published:** 2020-05-21

**Authors:** Martin P. Barr, Steven G. Gray, Andreas C. Hoffmann, Ralf A. Hilger, Juergen Thomale, John D. O’Flaherty, Dean A. Fennell, Derek Richard, John J. O’Leary, Kenneth J. O’Byrne

In Fig 11A of this article [1], the 12h MOR CisR image is duplicated in error as representing the 4h MOR CisR result. In the revised Fig 11 provided here, the 4h MOR CisR has been replaced with the correct data from the original experiment. The figure legend has been updated to clarify methods used to obtain the quantitative results.

**Fig 11:**
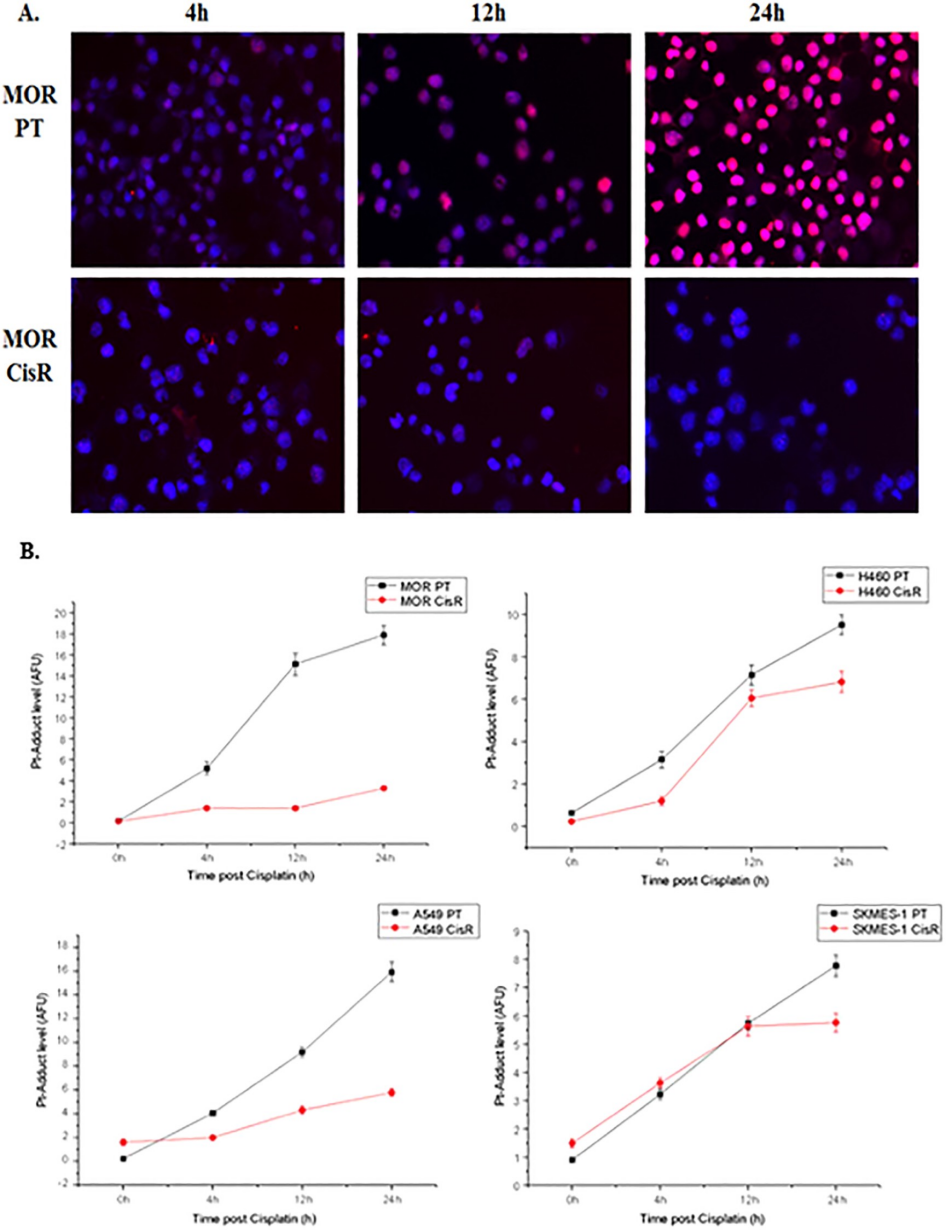
Cisplatin-DNA adduct formation and immunofluorescence. Lung cancer cell lines were treated with cisplatin for up to 24 h and fixed on Superfrost Gold Slides using ice-cold methanol. Cells were stained overnight at 4°C using a primary antibody that specifically recognizes CDDP-GpG DNA adducts (RC-18). Antibody binding was detected using an anti-rat Cy3^®^-labelled antibody and counterstained using DAPI (1 μg/ml (w/v). Images were acquired on an Axioplan fluorescence microscope (A). The quantitative data for adduct levels (B) in all cell lines (MOR, H460, A549 and SKMES-1) were measured using the ACAS-6.0 image analysis system as described in the Materials & Methods section. This CCD camera-based methodology does not deliver photographic images but directly measures the area of individual nuclei, their DNA content and the levels of Pt adduct-derived fluorescence signals from automatically selected pixels. This is represented as relative numbers for all three parameters for 100–500 cells. These are then converted into AFU (arbitrary fluorescence units) values representing the relative adduct level of a single nucleus. The graphics represented in Figure 11B show mean values of approximately 200 nuclei per time point ±95% confidence intervals. In parallel with these analyses, cell images for all cell lines were acquired using Axioplan fluorescence microscopy.

The original images underlying the updated Fig 11A are included in S1 and S2 Files of this notice; S3–S6 Files include image and quantitative data supporting the results in Fig 11B. The original data underlying other results in the article are available upon request from the authors.

A member of *PLOS ONE*’s Editorial Board confirmed that the revised version of Fig 11 and the data provided support the results as reported in the original article.

The authors apologize for the error in the published article.

## Supporting information

S1 File Powerpoint file showing the original Fig 11A (with error), updated Fig 11A, and the Cy3, DAPI, and DAPI Cy3 overlay images used in the MOR CisR (4h) panel of the updated Fig 11A. (PPT)

S2 File MOR cells 4h, 12h and 24h (Cy3, DAPI and overlay). (ZIP)

S3 File H460 cells 4h, 12h and 24h (Cy3, DAPI and overlay). (ZIP)

S4 File A549 cells 4h, 12h and 24h (Cy3, DAPI and overlay). (ZIP)

S5 File SKMES-1 cells 4h, 12h and 24h (Cy3, DAPI and overlay). (ZIP)

S6 File Quantitative data (Adduct Kinetics). (XLSX)
