# Supplementary material for: Correction: Generation and Characterisation of Cisplatin-Resistant Non-Small Cell Lung Cancer Cell Lines Displaying a Stem-Like Signature
Source: PLoS One. 2020 May 21;15(5):e0233739. doi: 10.1371/journal.pone.0233739 (PMC7241695; doi:10.1371/journal.pone.0233739)
Supplement: S1 File — (PPT) [file pone.0233739.s001.ppt]

## Slide 1
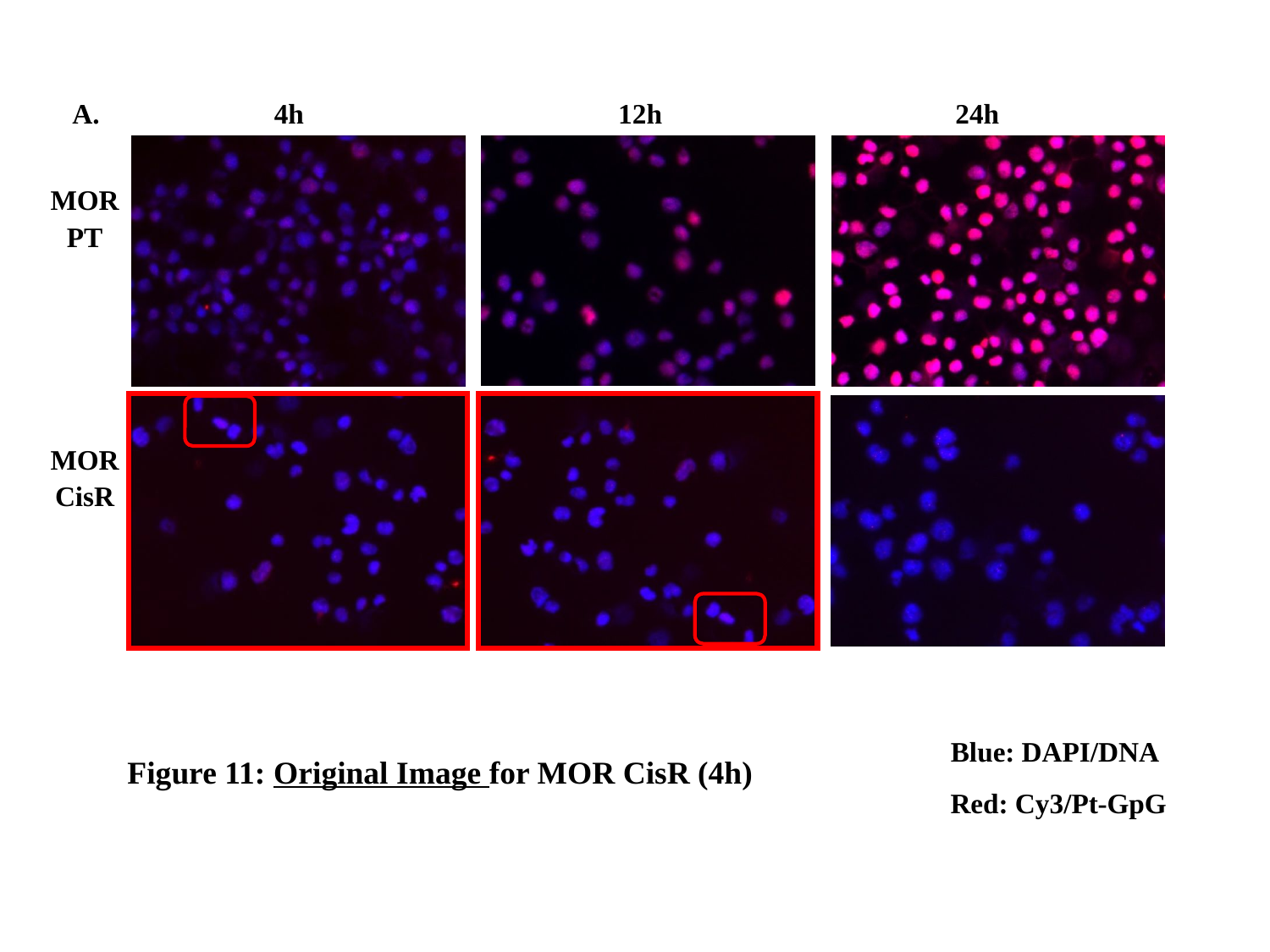

A. 4h 12h 24h
MOR
PT
MOR
CisR
Blue: DAPI/DNA
 Red: Cy3/Pt-GpG
Figure 11: Original Image for MOR CisR (4h)

## Slide 2
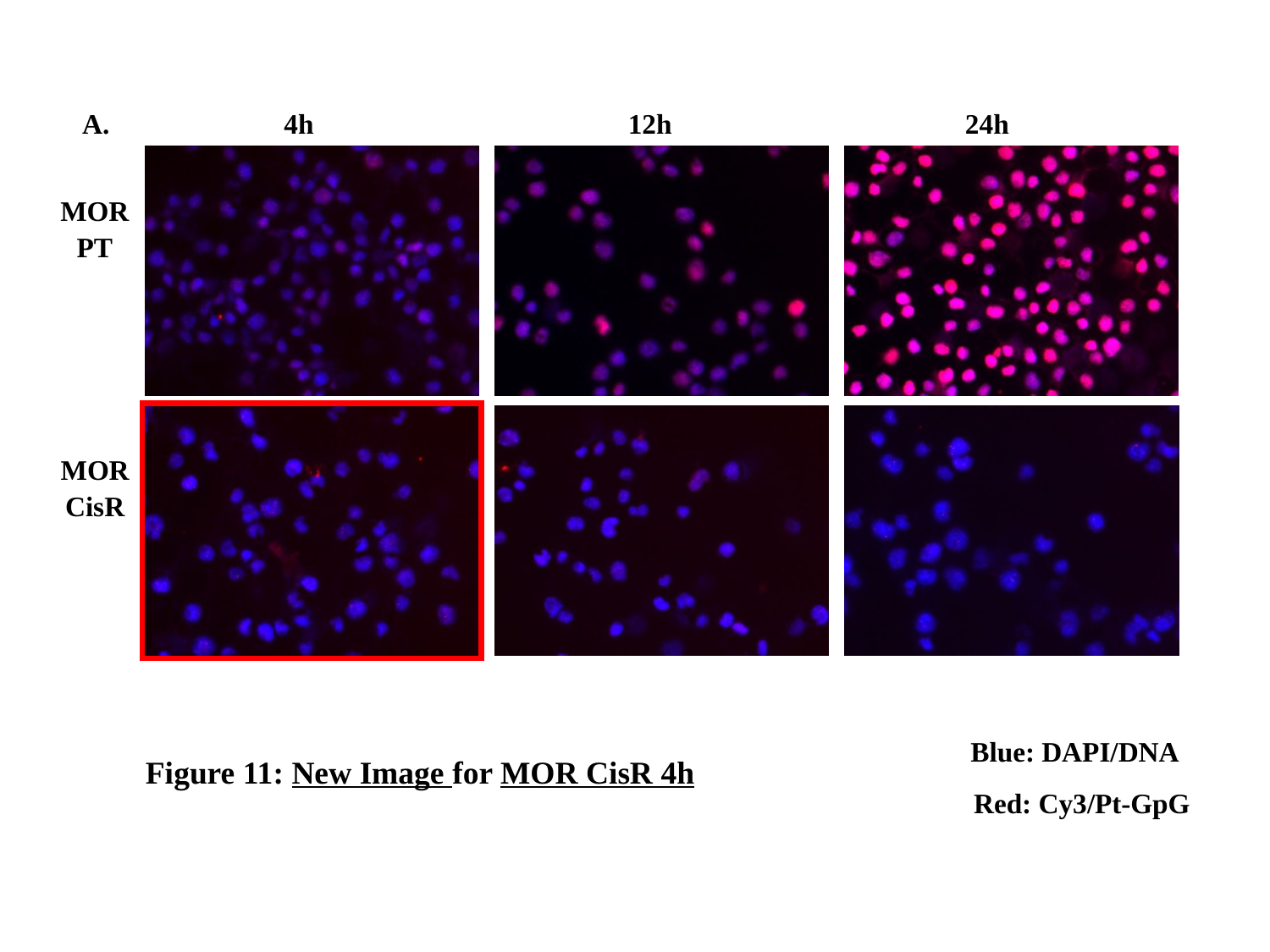

A. 4h 12h 24h
MOR
PT
MOR
CisR
Blue: DAPI/DNA
 Red: Cy3/Pt-GpG
 Figure 11: New Image for MOR CisR 4h

## Slide 3
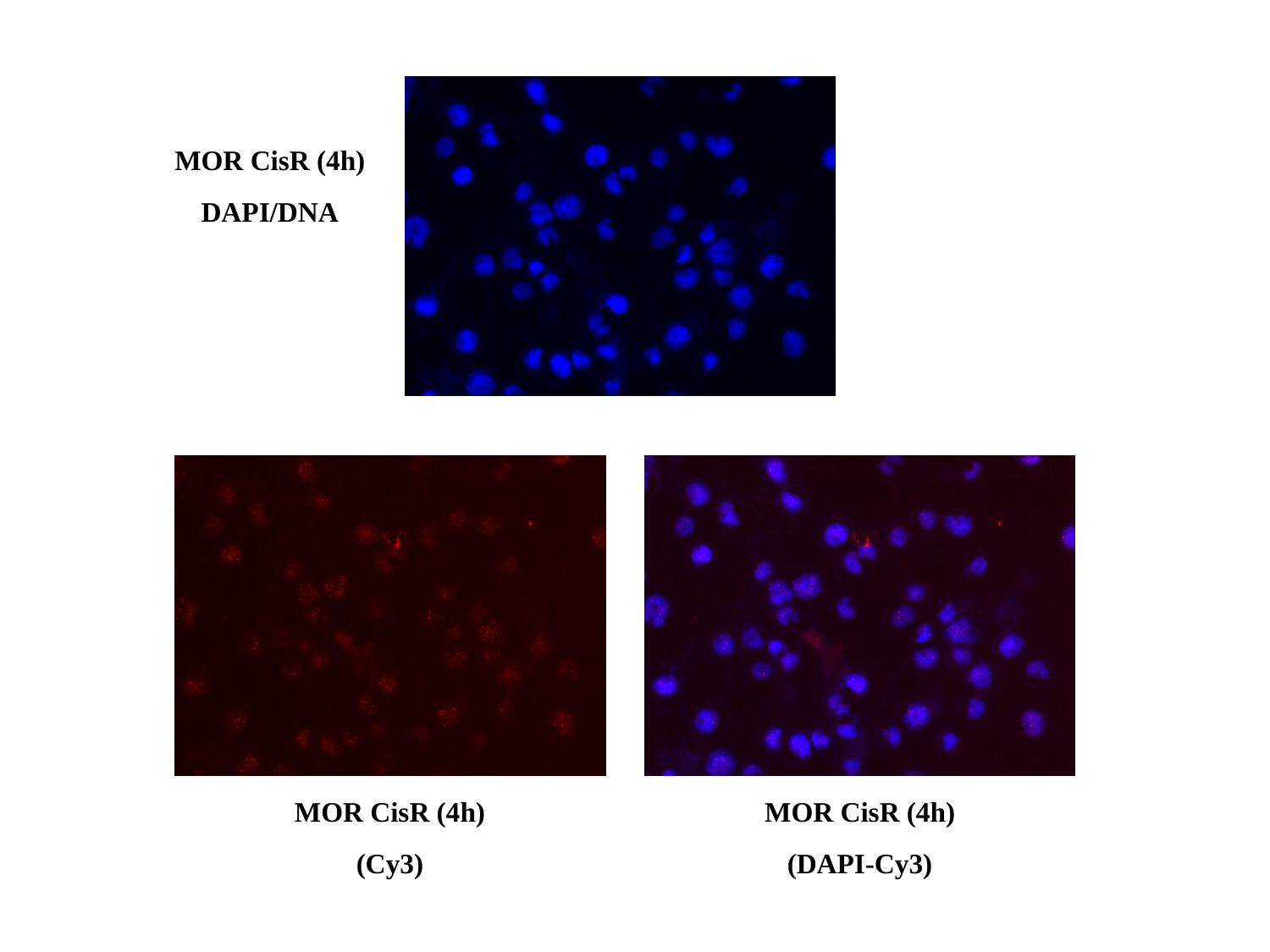

MOR CisR (4h)
DAPI/DNA
MOR CisR (4h)
(Cy3)
MOR CisR (4h)
(DAPI-Cy3)
