# Supplementary figures and images for: Correction: Generation and Characterisation of Cisplatin-Resistant Non-Small Cell Lung Cancer Cell Lines Displaying a Stem-Like Signature
Source: PLoS One. 2020 May 21;15(5):e0233739. doi: 10.1371/journal.pone.0233739 (PMC7241695; doi:10.1371/journal.pone.0233739)

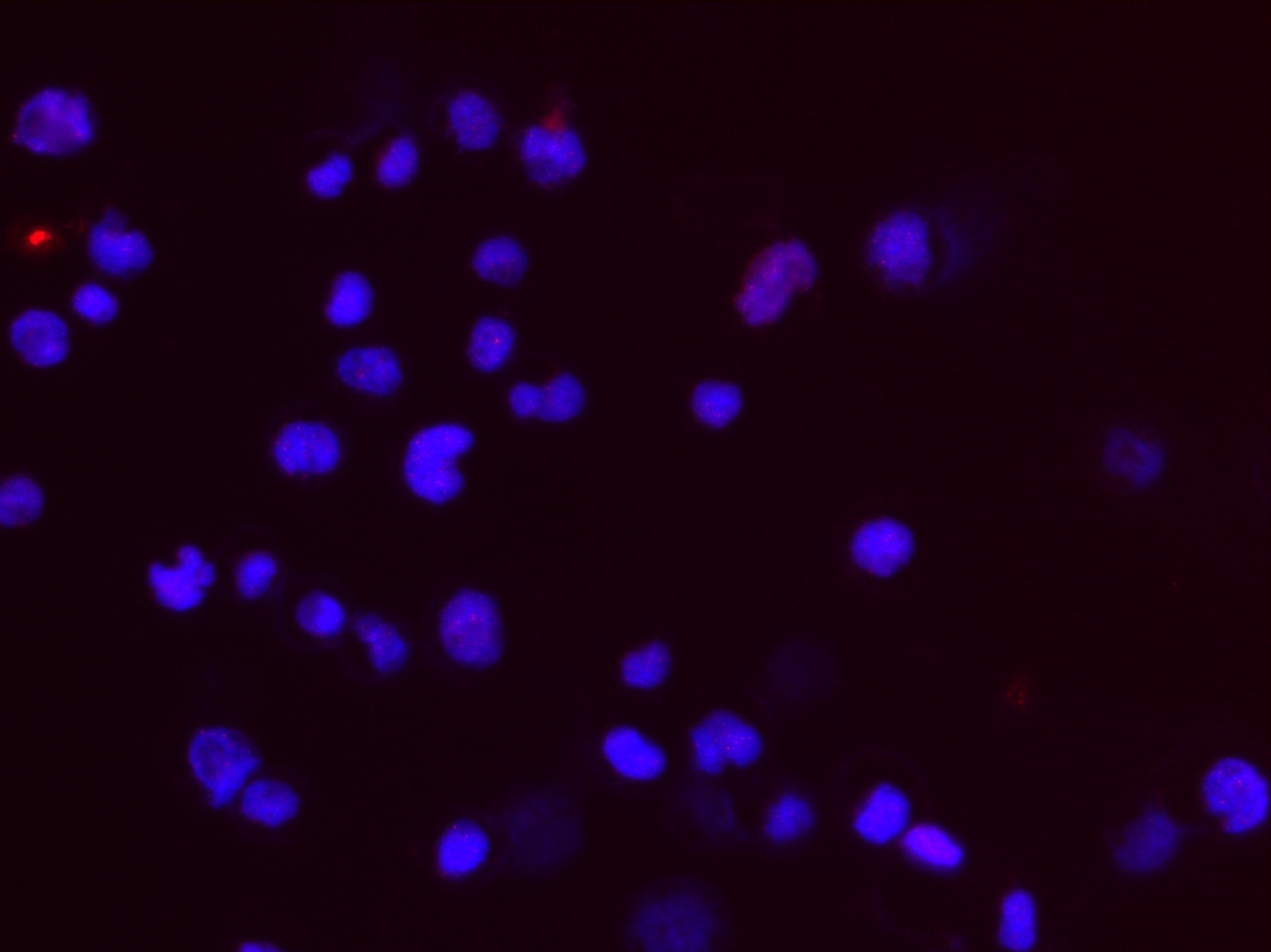

Supplement: S2 File — (ZIP) [file pone.0233739.s002.zip › S2_File/MOR CisR (12h) Res-4-2012-0006_(c2+c3).JPG]

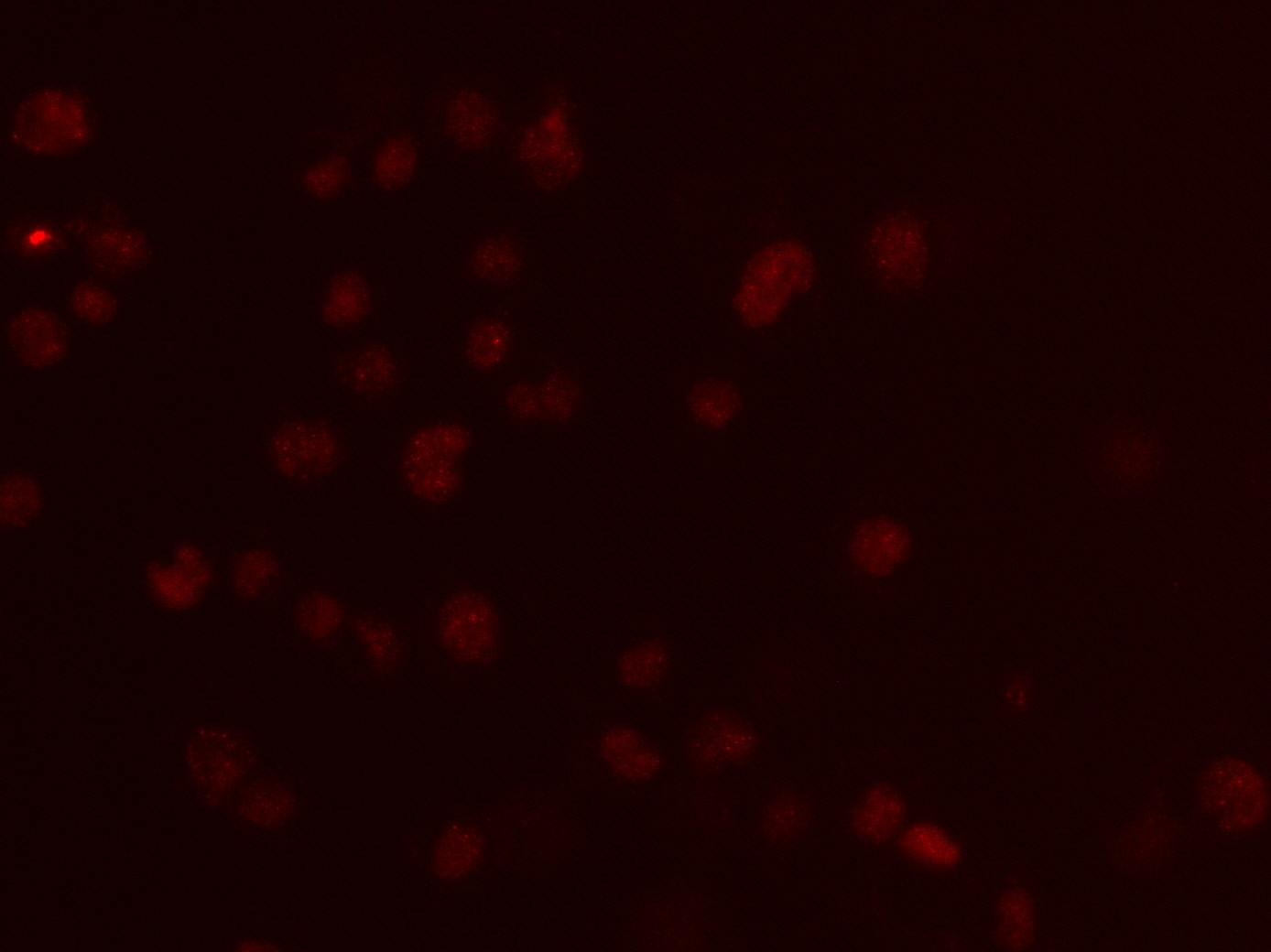

Supplement: S2 File — (ZIP) [file pone.0233739.s002.zip › S2_File/MOR CisR (12h) Res-4-2012-0006_c2.JPG]

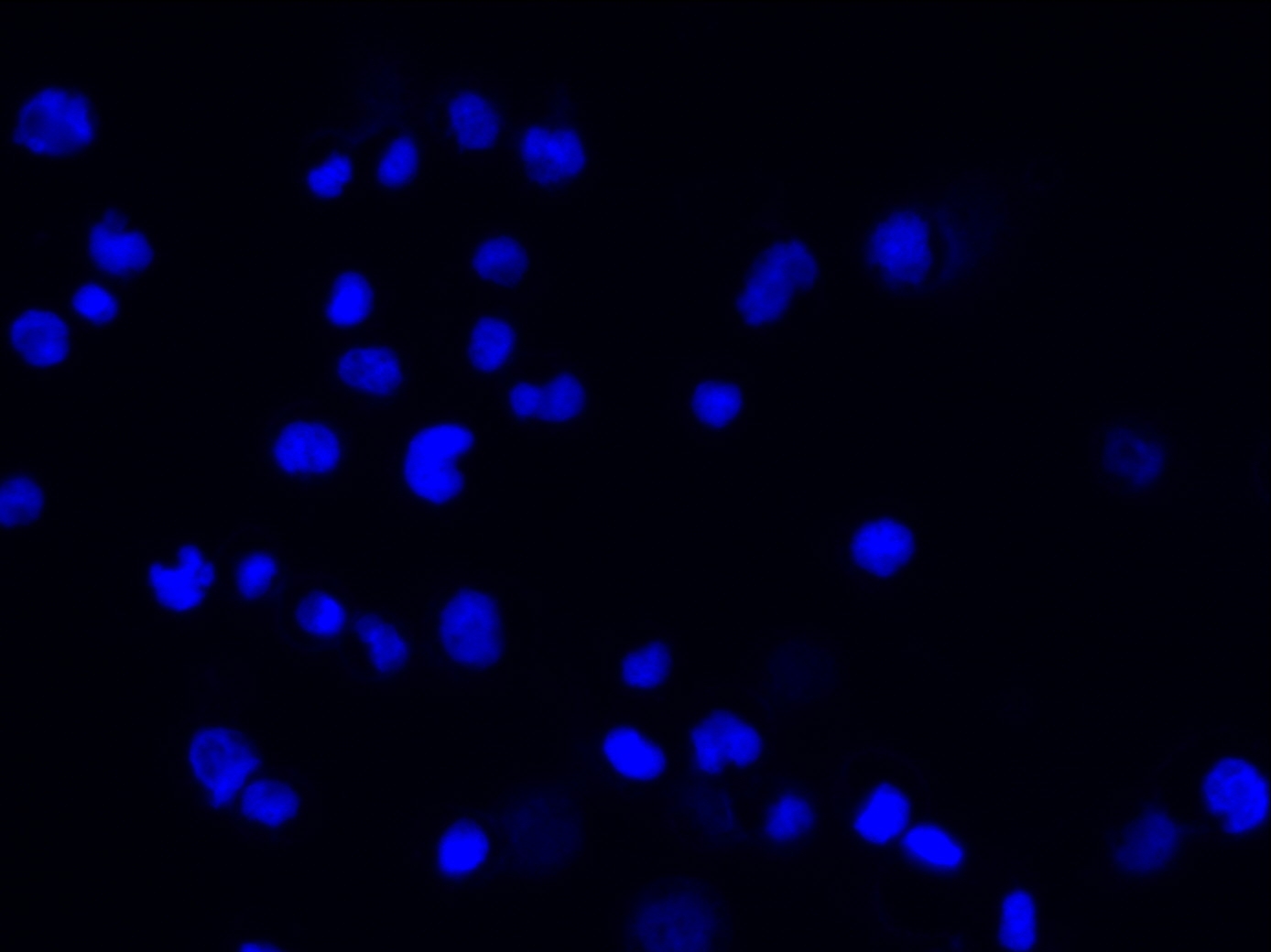

Supplement: S2 File — (ZIP) [file pone.0233739.s002.zip › S2_File/MOR CisR (12h) Res-4-2012-0006_c3.JPG]

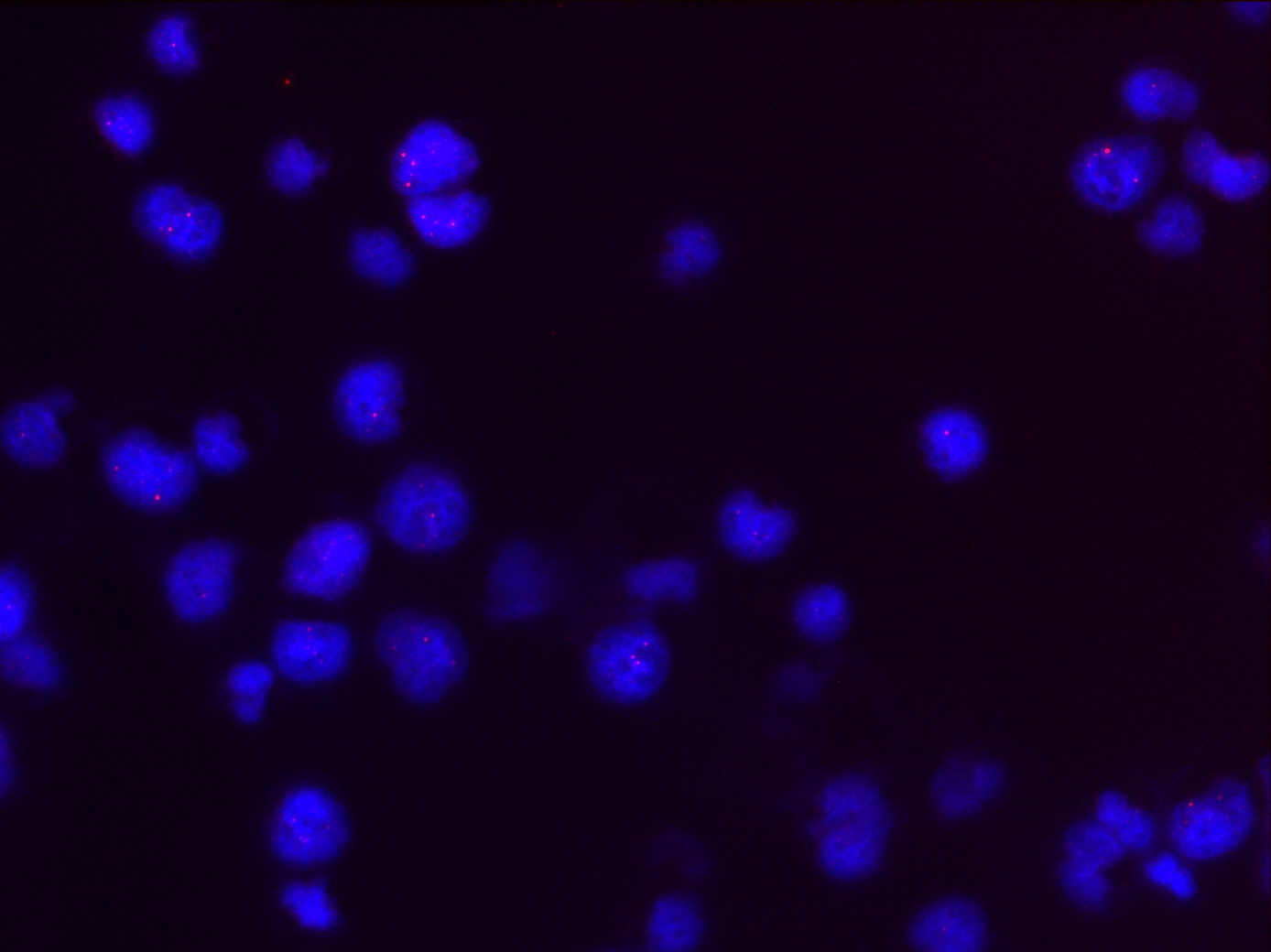

Supplement: S2 File — (ZIP) [file pone.0233739.s002.zip › S2_File/MOR CisR (24h) Res-2-2012-0004_(c2+c3).JPG]

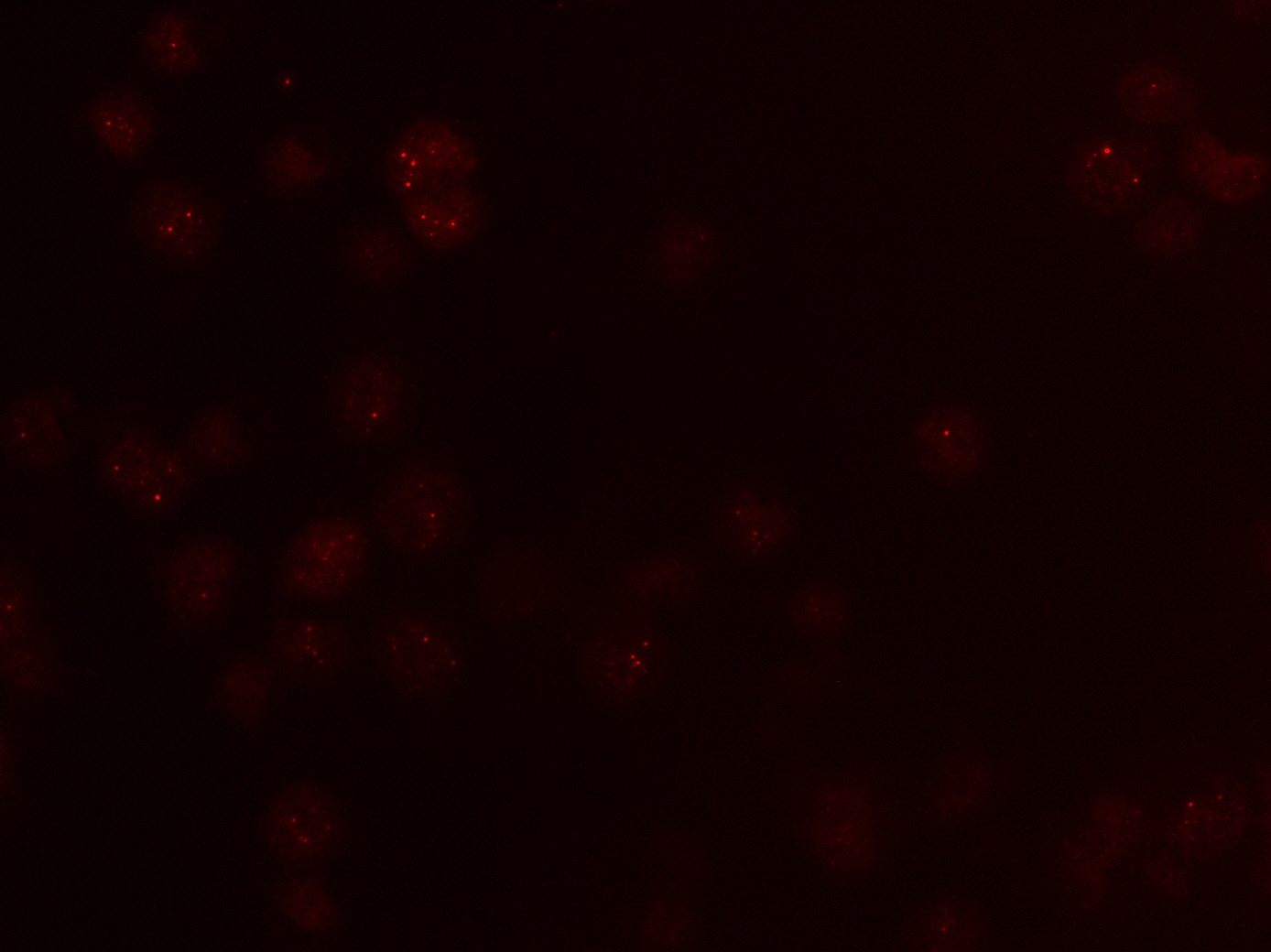

Supplement: S2 File — (ZIP) [file pone.0233739.s002.zip › S2_File/MOR CisR (24h) Res-2-2012-0004_c2.JPG]

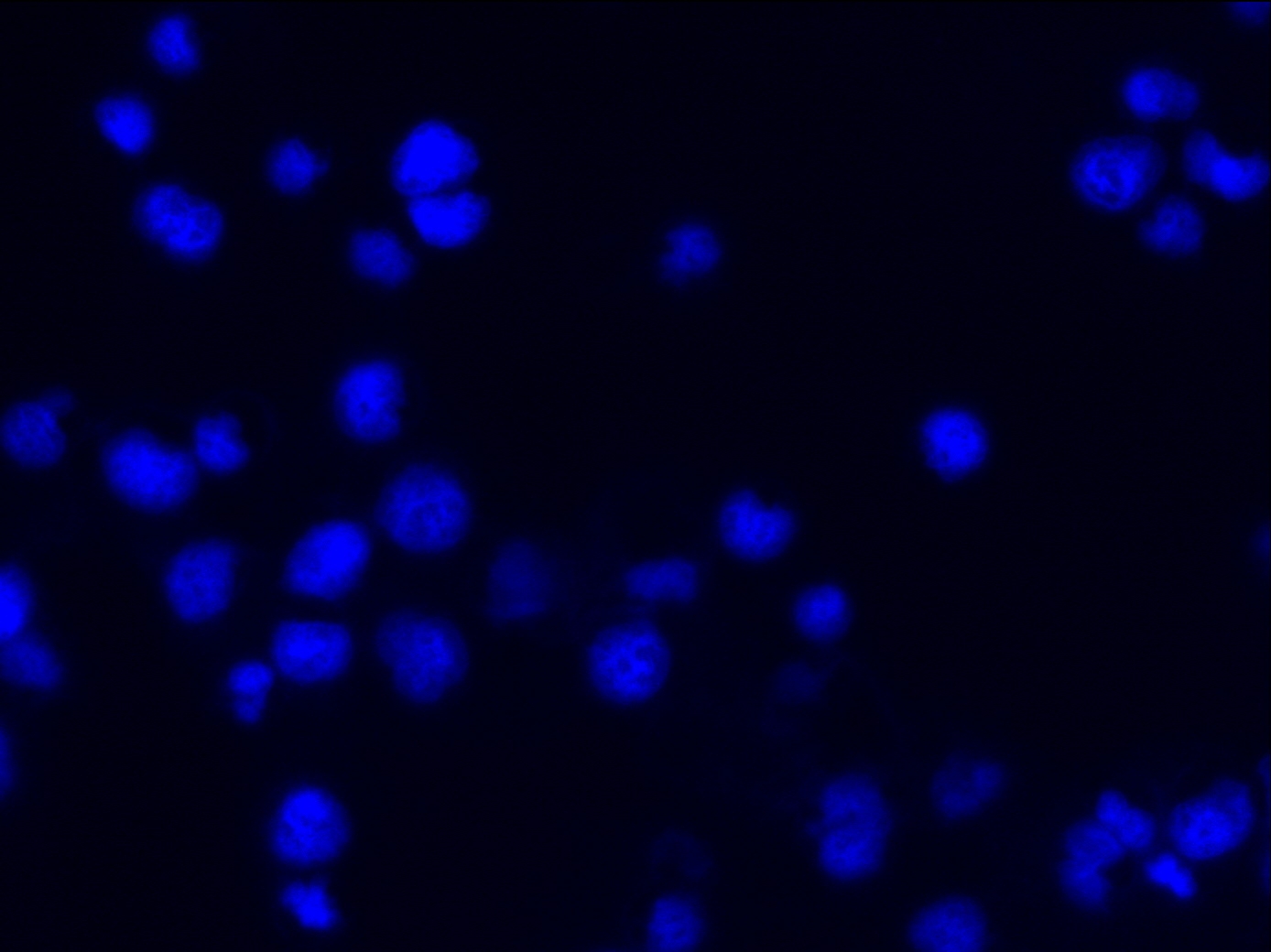

Supplement: S2 File — (ZIP) [file pone.0233739.s002.zip › S2_File/MOR CisR (24h) Res-2-2012-0004_c3.JPG]

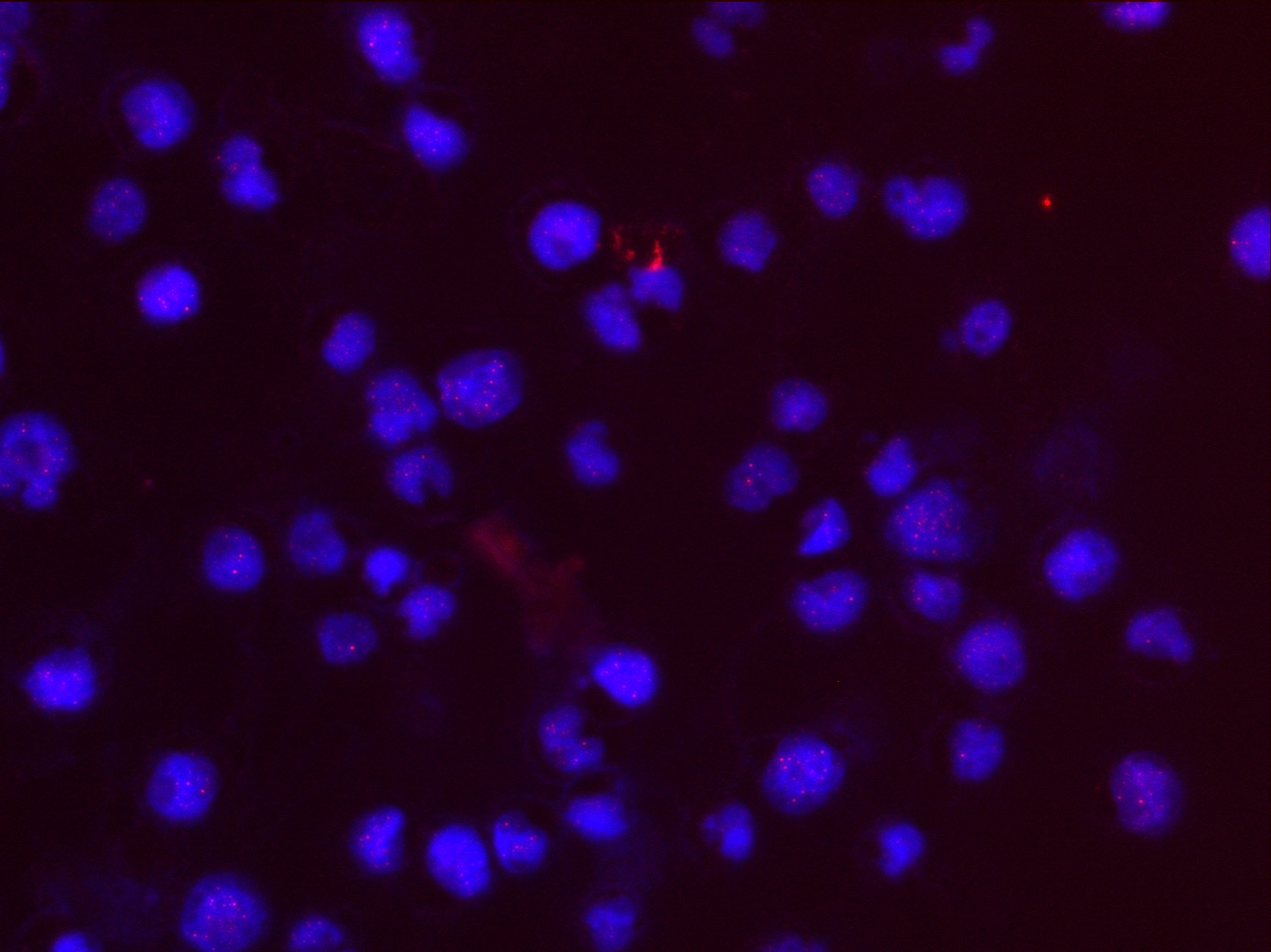

Supplement: S2 File — (ZIP) [file pone.0233739.s002.zip › S2_File/MOR CisR (4h) Res-3-4h-2012-0005_(c2+c3).JPG]

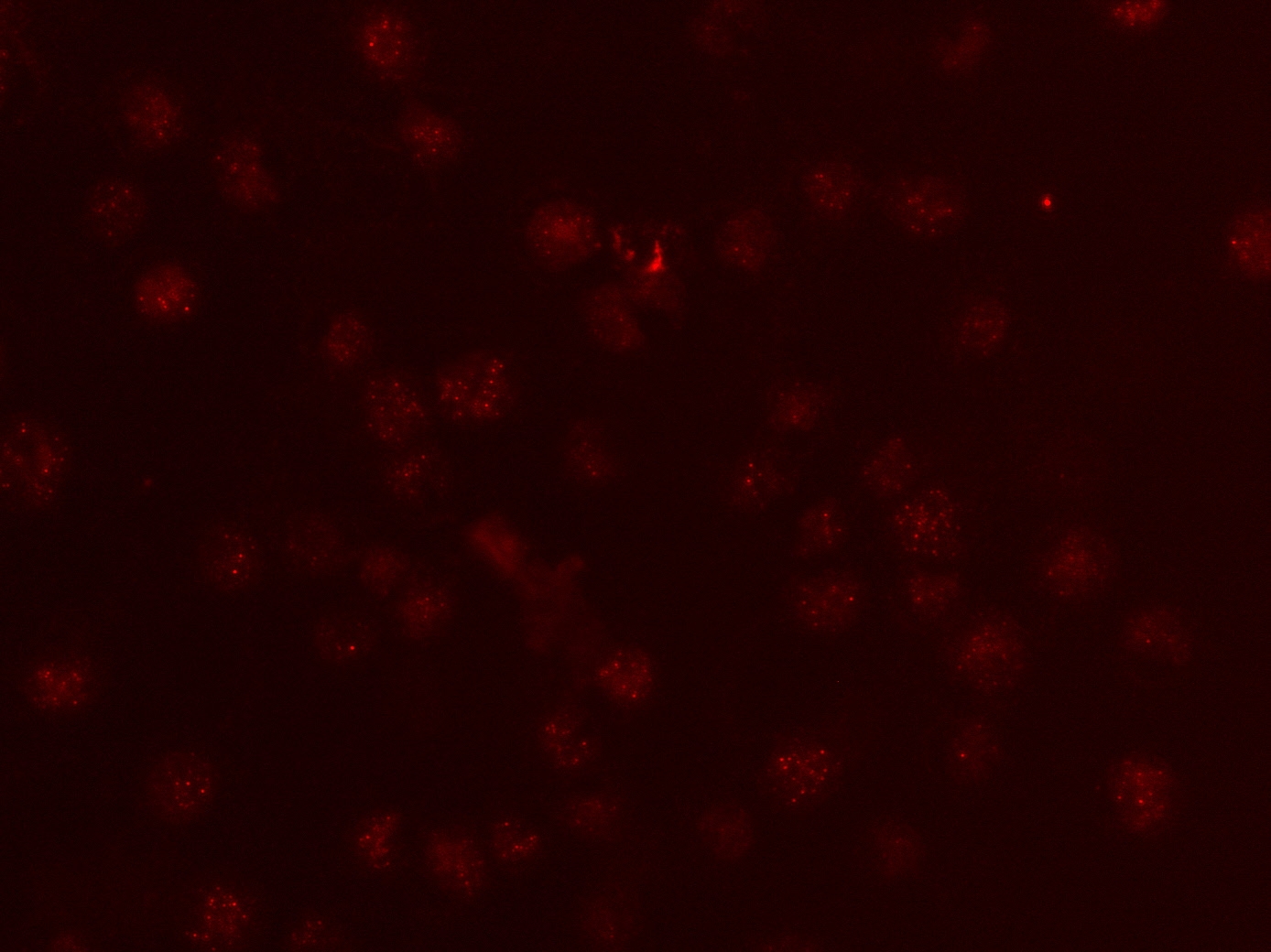

Supplement: S2 File — (ZIP) [file pone.0233739.s002.zip › S2_File/MOR CisR (4h) Res-3-4h-2012-0005_c2.JPG]

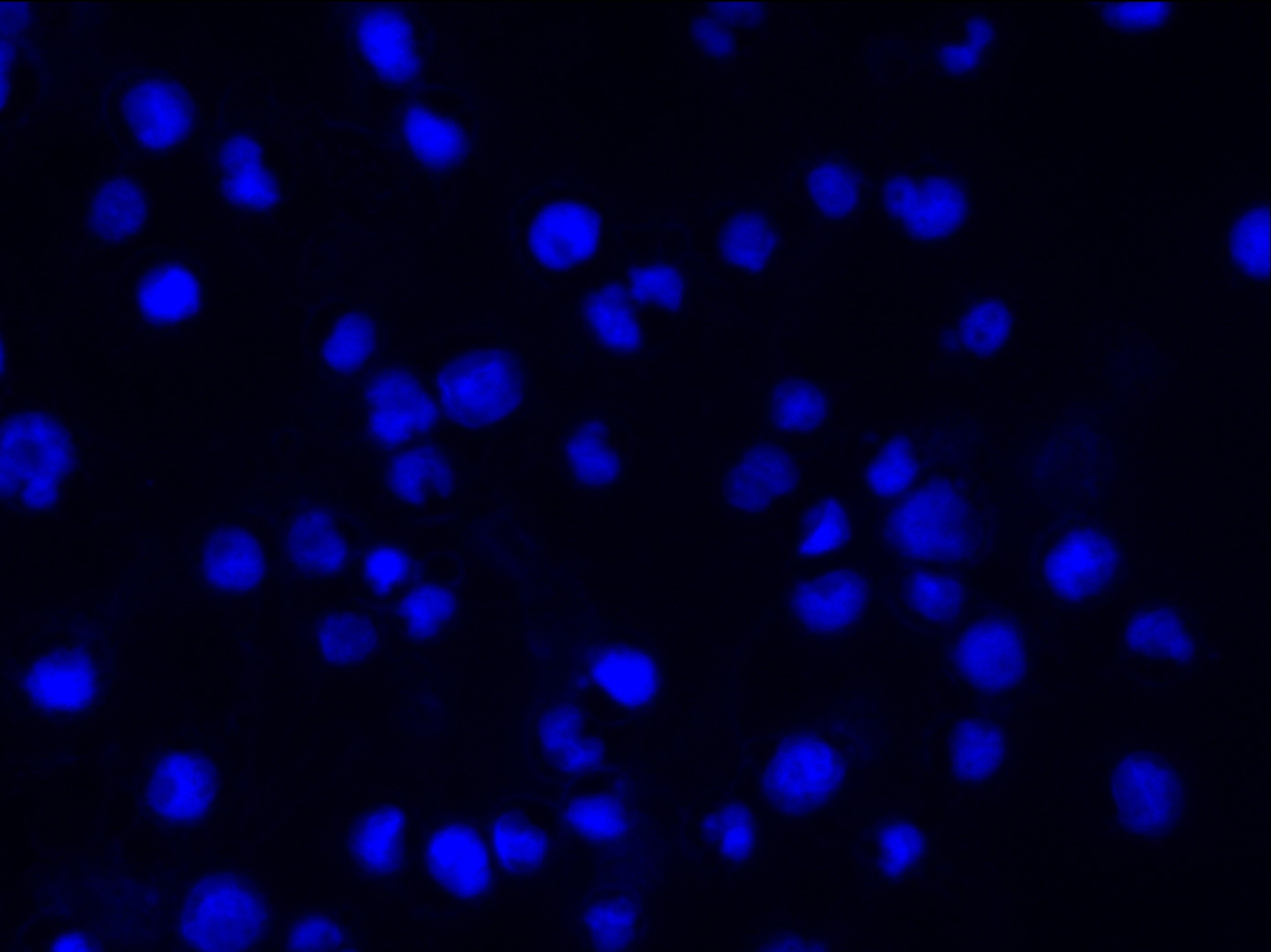

Supplement: S2 File — (ZIP) [file pone.0233739.s002.zip › S2_File/MOR CisR (4h) Res-3-4h-2012-0005_c3.JPG]

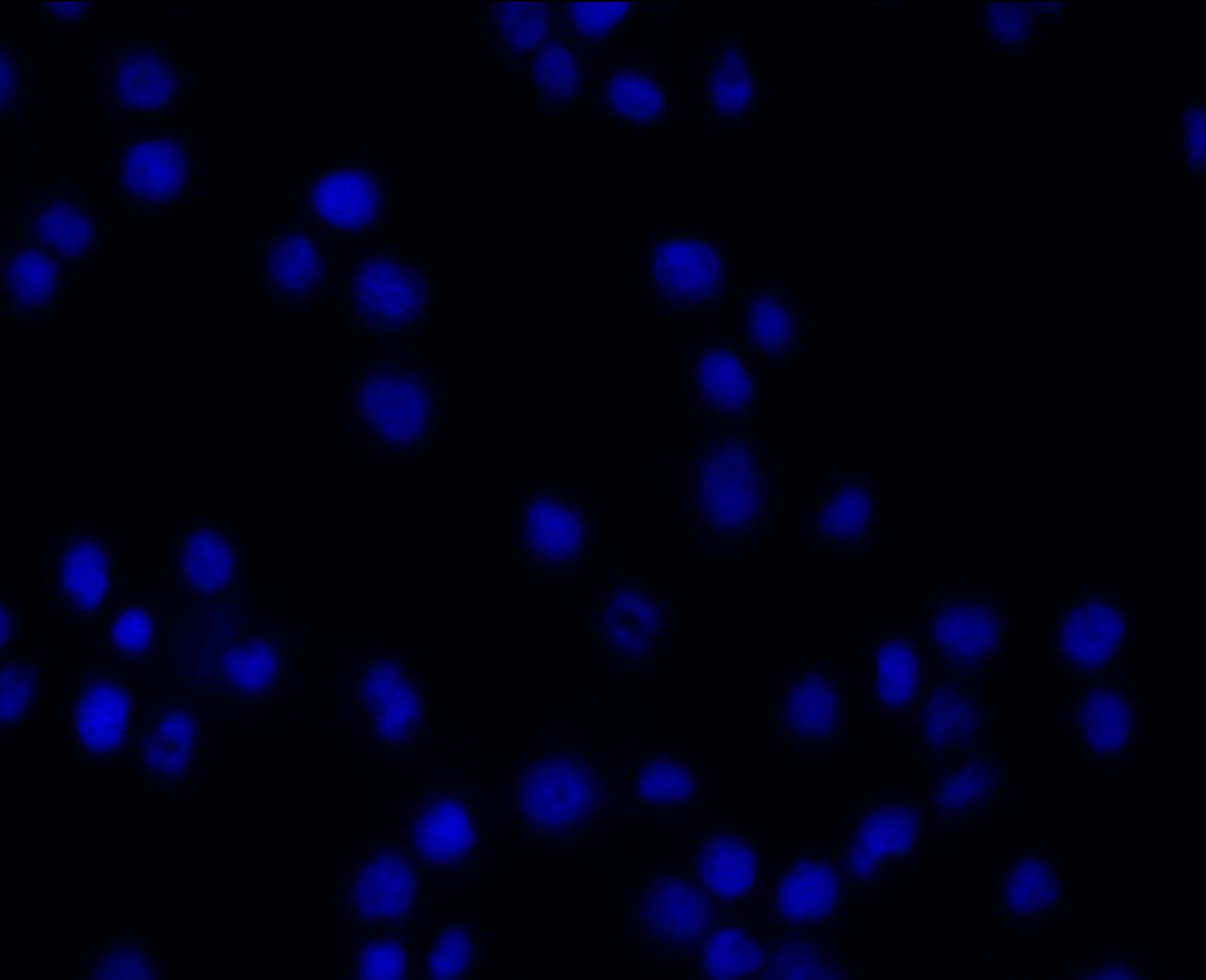

Supplement: S2 File — (ZIP) [file pone.0233739.s002.zip › S2_File/MOR Sens (12h) 2012-002-c2.jpg]

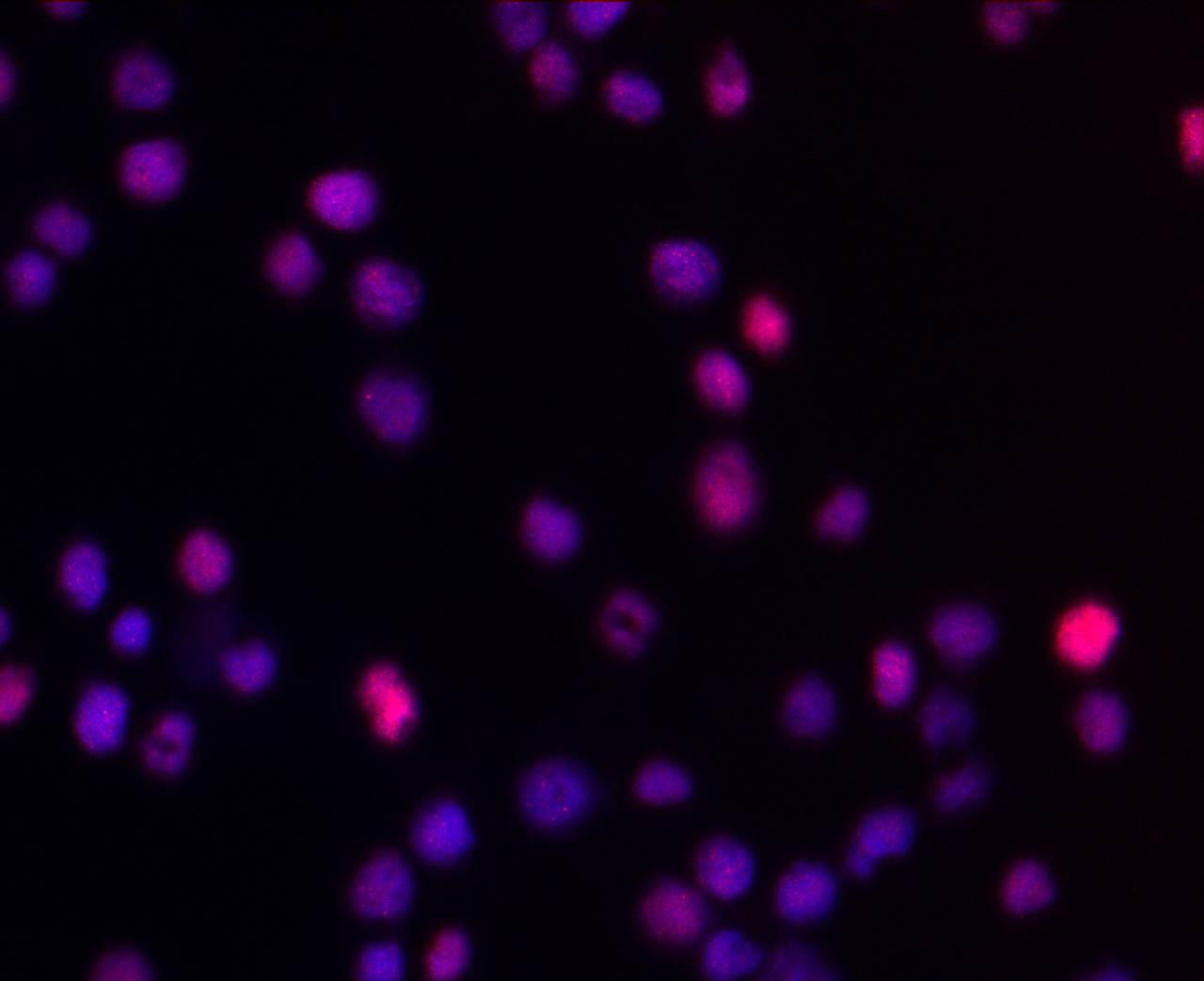

Supplement: S2 File — (ZIP) [file pone.0233739.s002.zip › S2_File/MOR Sens (12h) 2012-002-c2+c3.jpg]

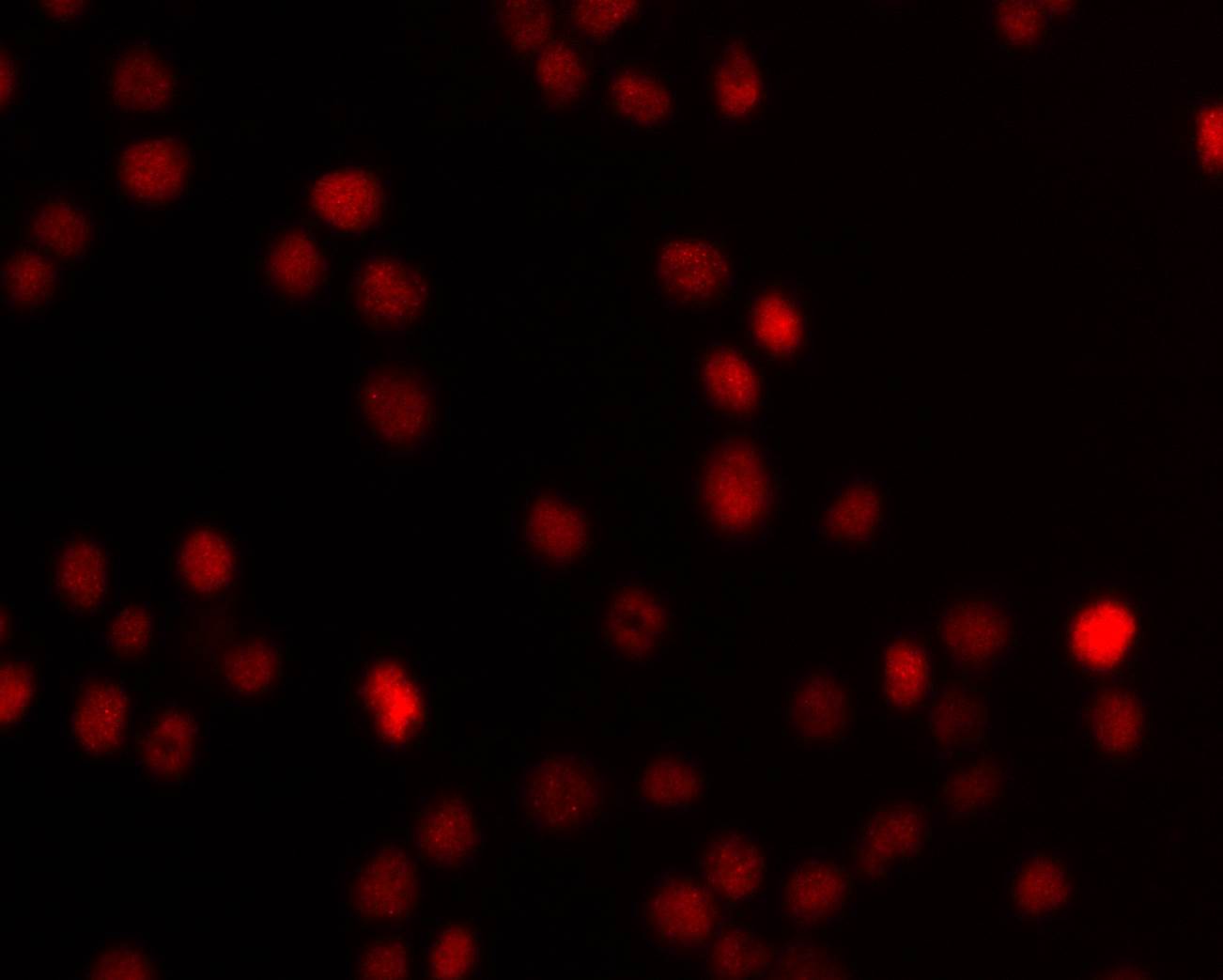

Supplement: S2 File — (ZIP) [file pone.0233739.s002.zip › S2_File/MOR Sens (12h) 2012-002-c3.jpg]

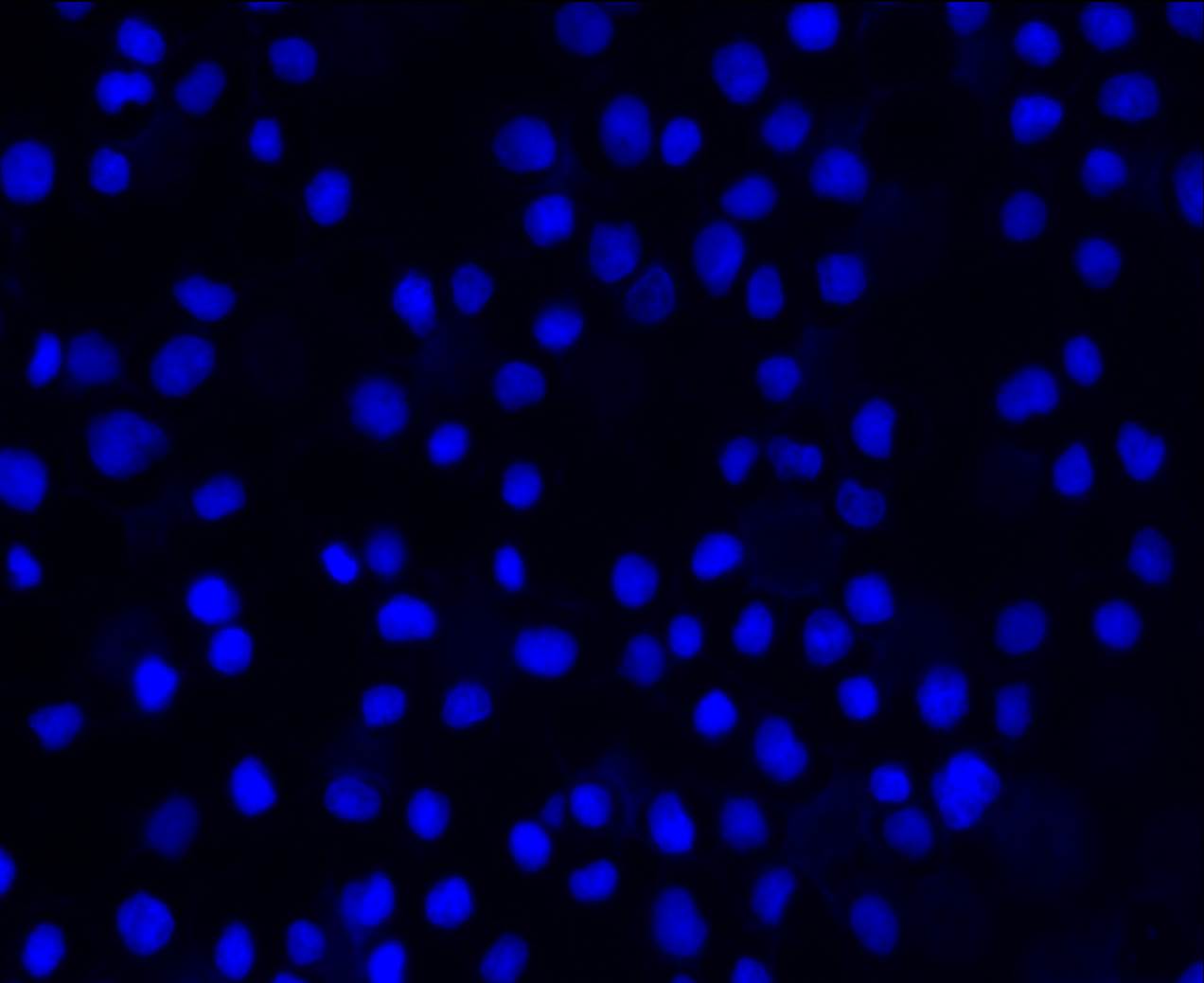

Supplement: S2 File — (ZIP) [file pone.0233739.s002.zip › S2_File/MOR Sens (24h) 2012-002-c2.jpg]

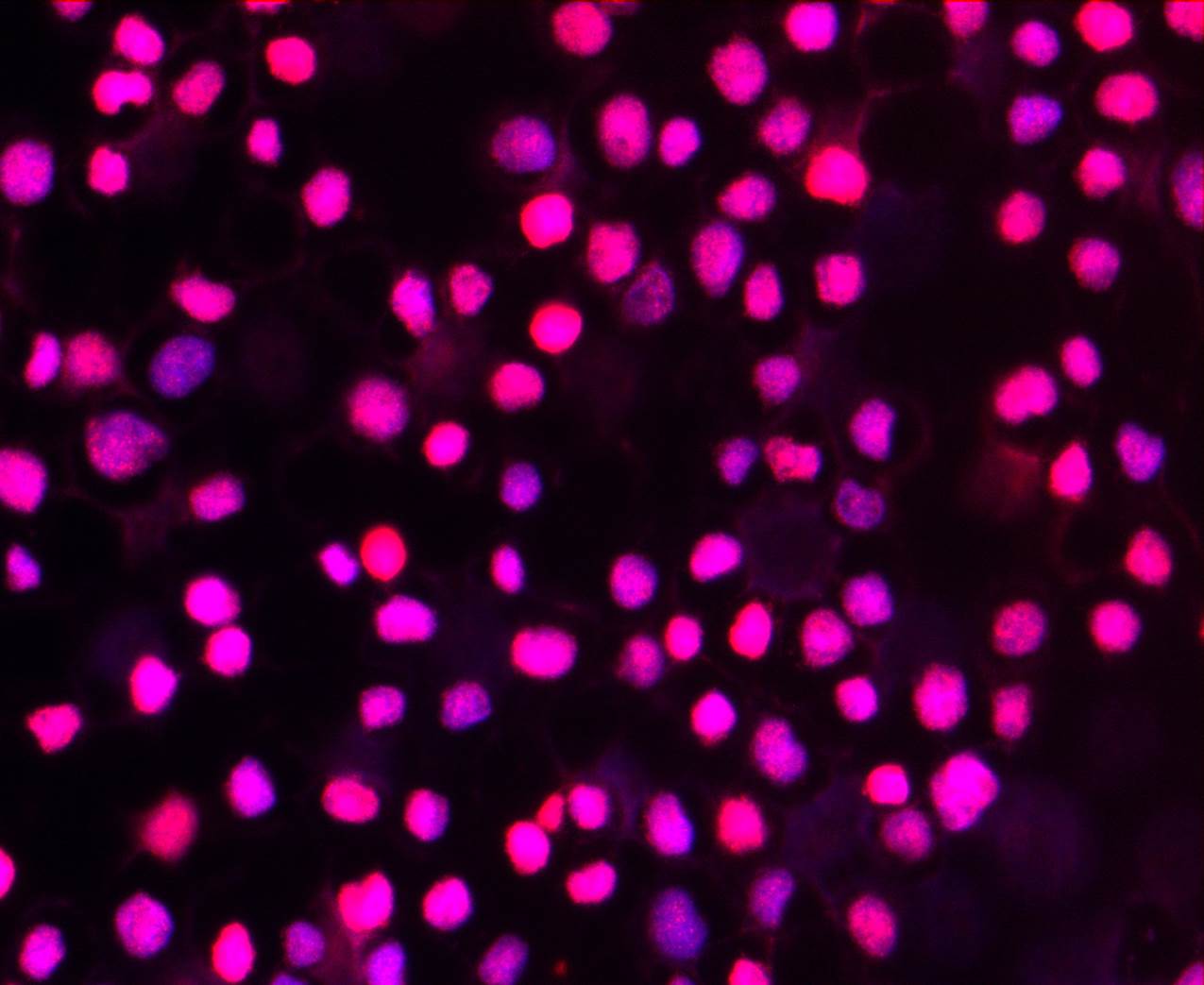

Supplement: S2 File — (ZIP) [file pone.0233739.s002.zip › S2_File/MOR Sens (24h) 2012-002-c2+c3.jpg]

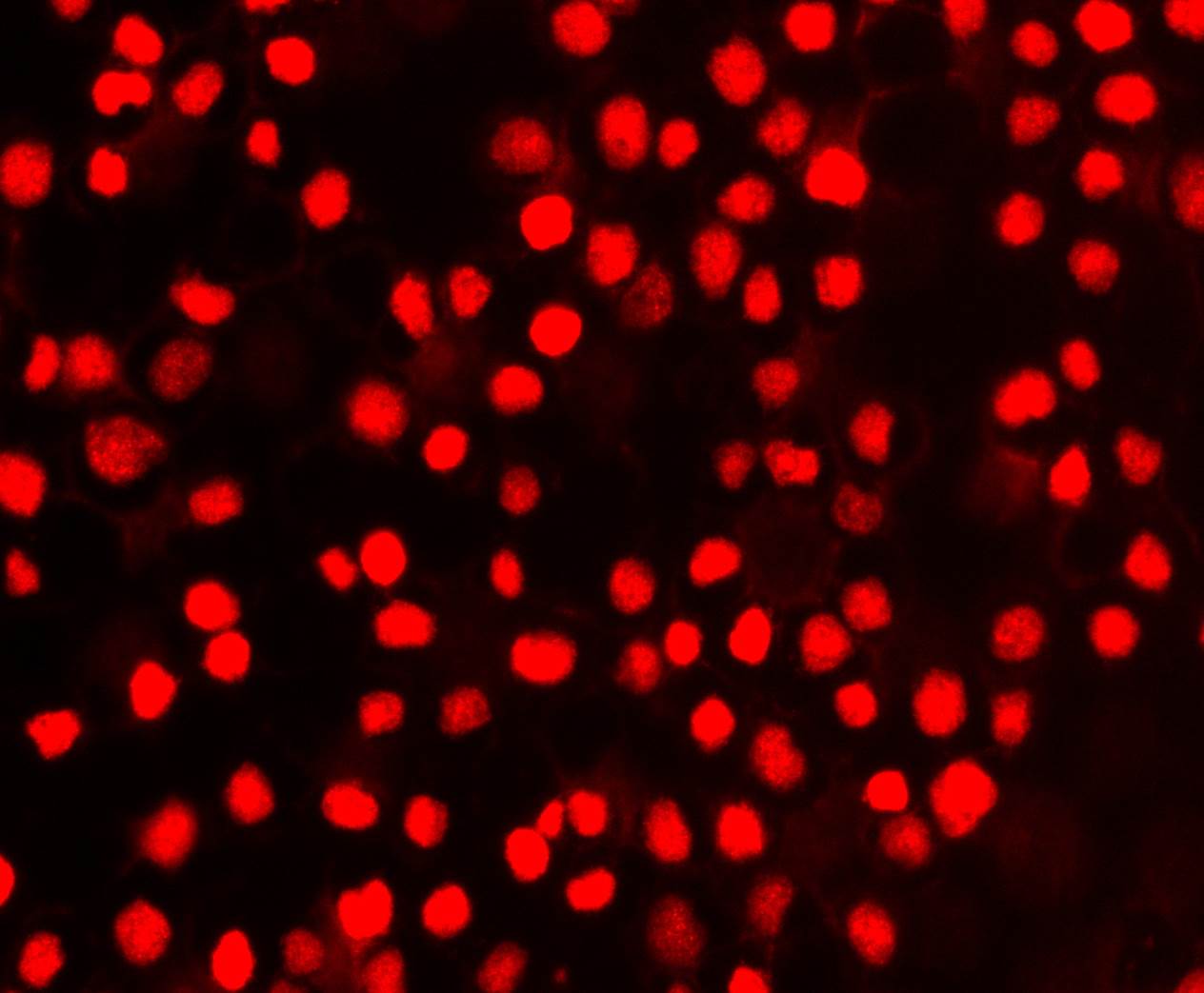

Supplement: S2 File — (ZIP) [file pone.0233739.s002.zip › S2_File/MOR Sens (24h) 2012-002-c3.jpg]

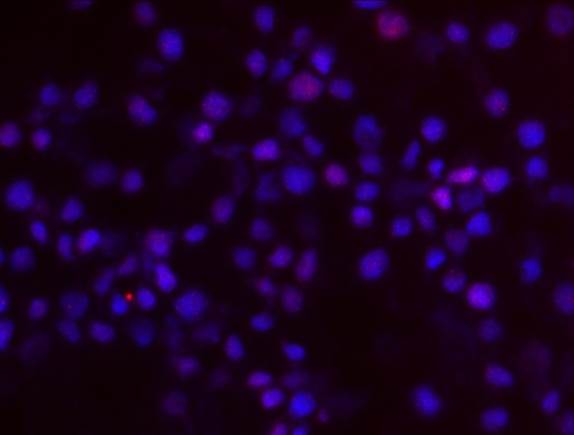

Supplement: S2 File — (ZIP) [file pone.0233739.s002.zip › S2_File/MOR Sens (4h) 2012-002-c2.jpg]

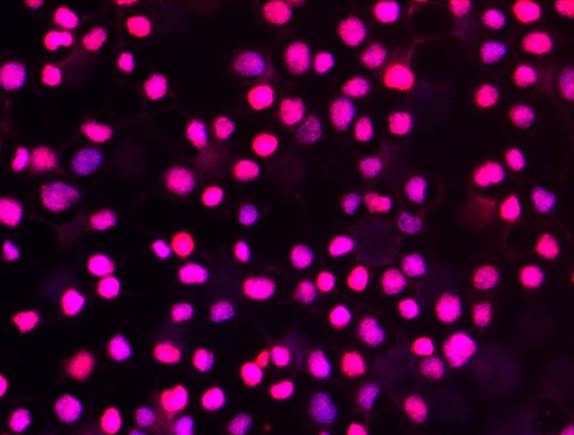

Supplement: S2 File — (ZIP) [file pone.0233739.s002.zip › S2_File/MOR Sens (4h) 2012-002-c2+c3.jpg]

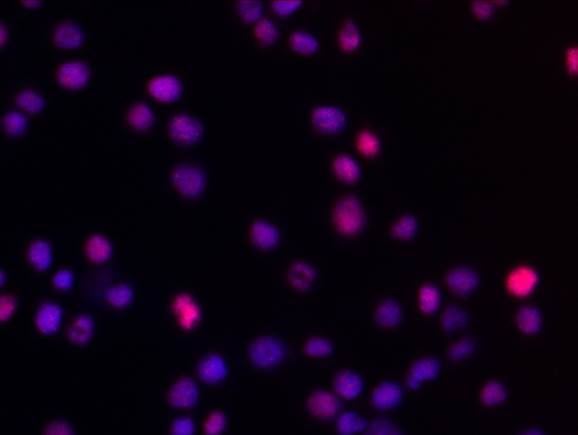

Supplement: S2 File — (ZIP) [file pone.0233739.s002.zip › S2_File/MOR Sens (4h) 2012-002-c3.jpg]

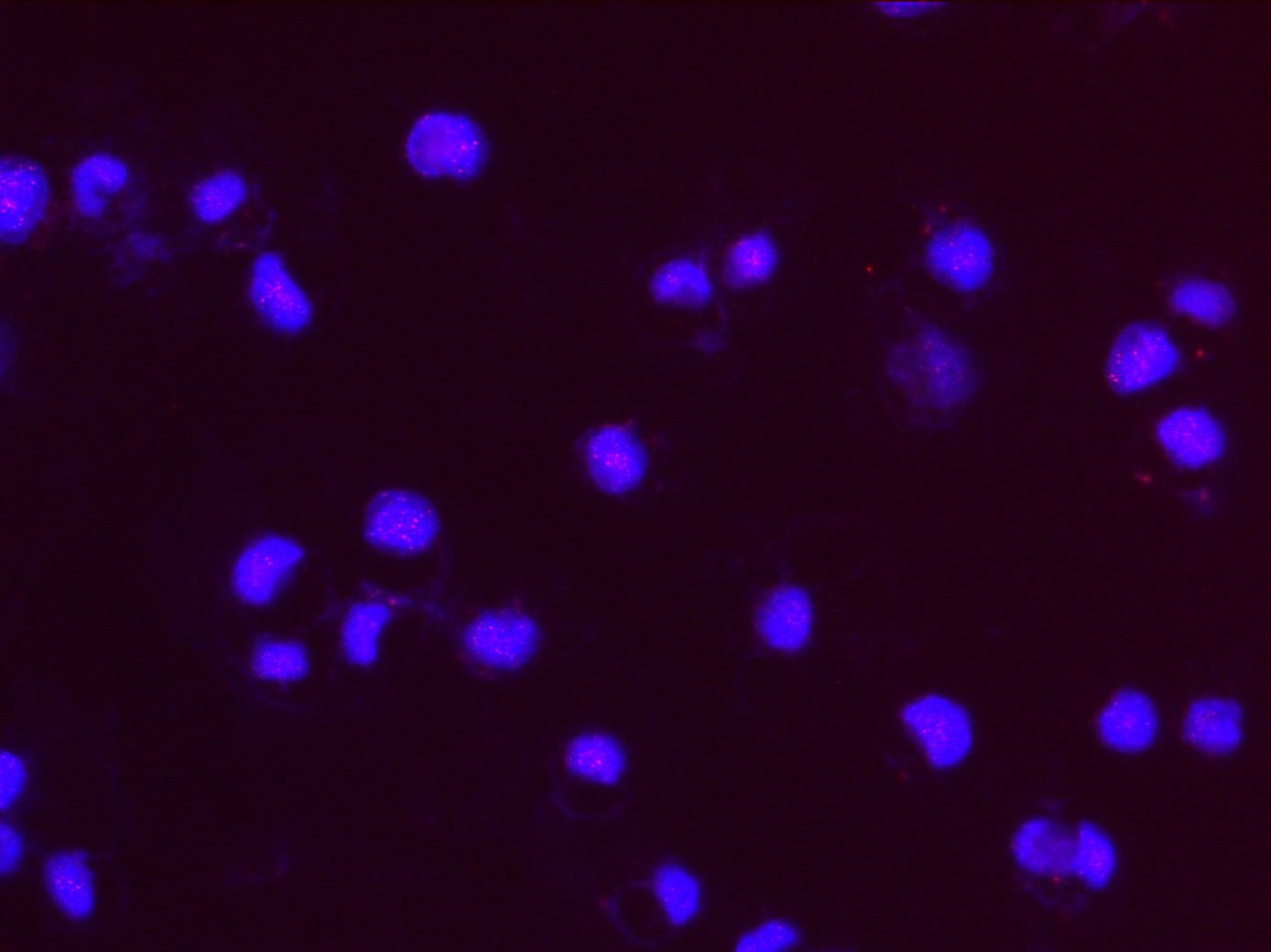

Supplement: S3 File — (ZIP) [file pone.0233739.s003.zip › S3_File/H 460 Res-12h-2012-0025_(c2+c3).JPG]

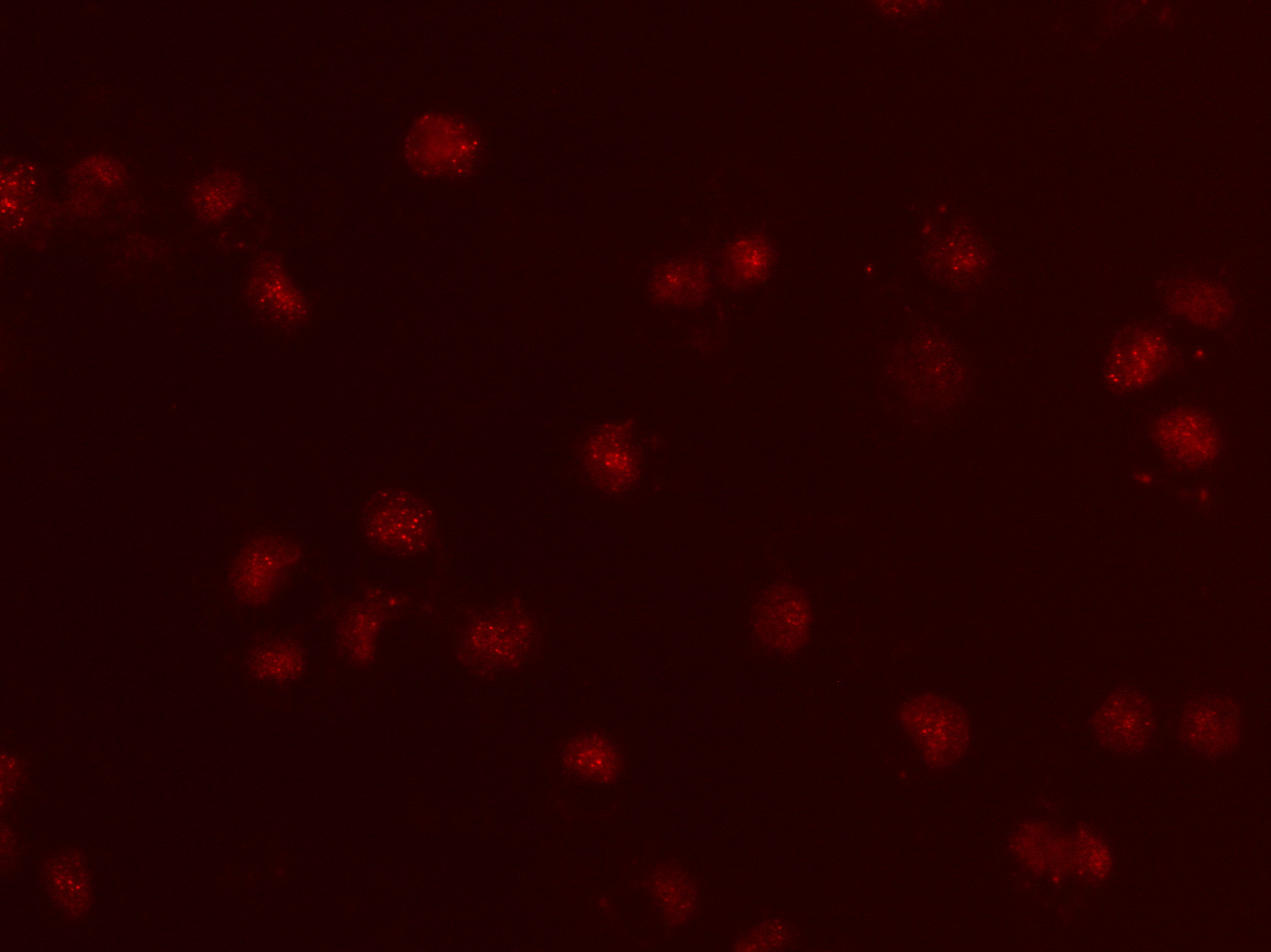

Supplement: S3 File — (ZIP) [file pone.0233739.s003.zip › S3_File/H 460 Res-12h-2012-0025_c2.JPG]

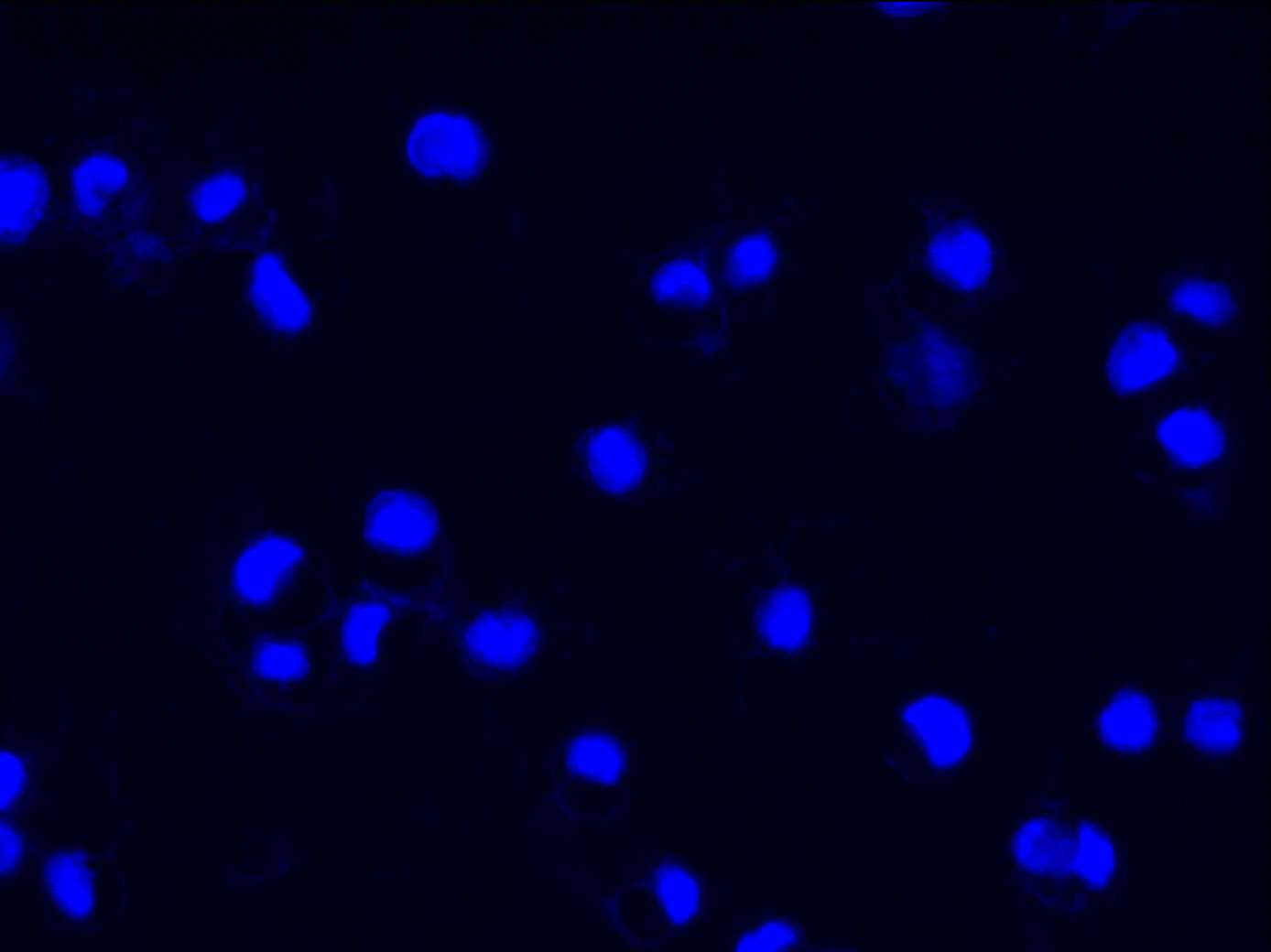

Supplement: S3 File — (ZIP) [file pone.0233739.s003.zip › S3_File/H 460 Res-12h-2012-0025_c3.JPG]

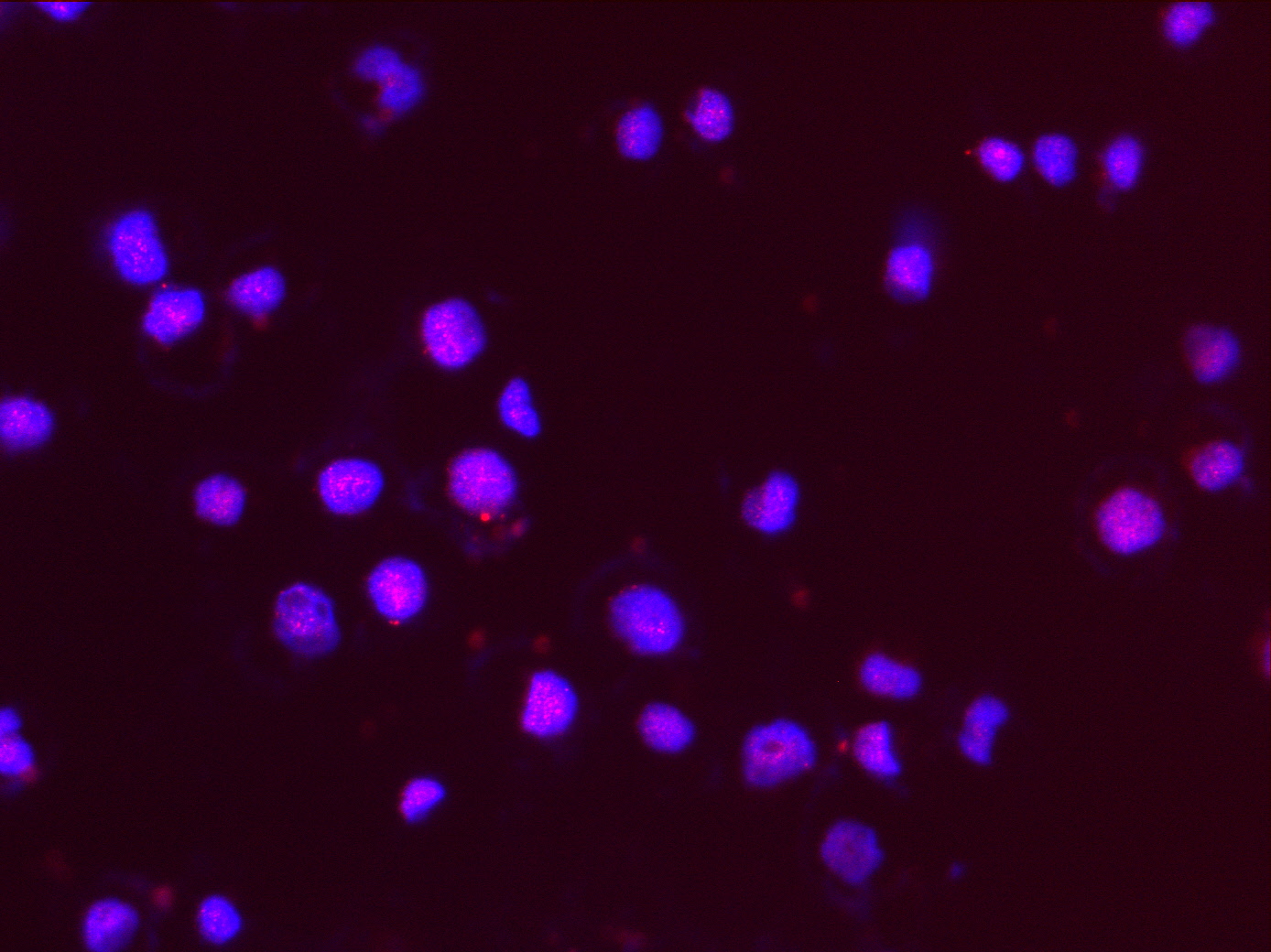

Supplement: S3 File — (ZIP) [file pone.0233739.s003.zip › S3_File/H 460 Res-24h-2012-0026_(c2+c3).JPG]

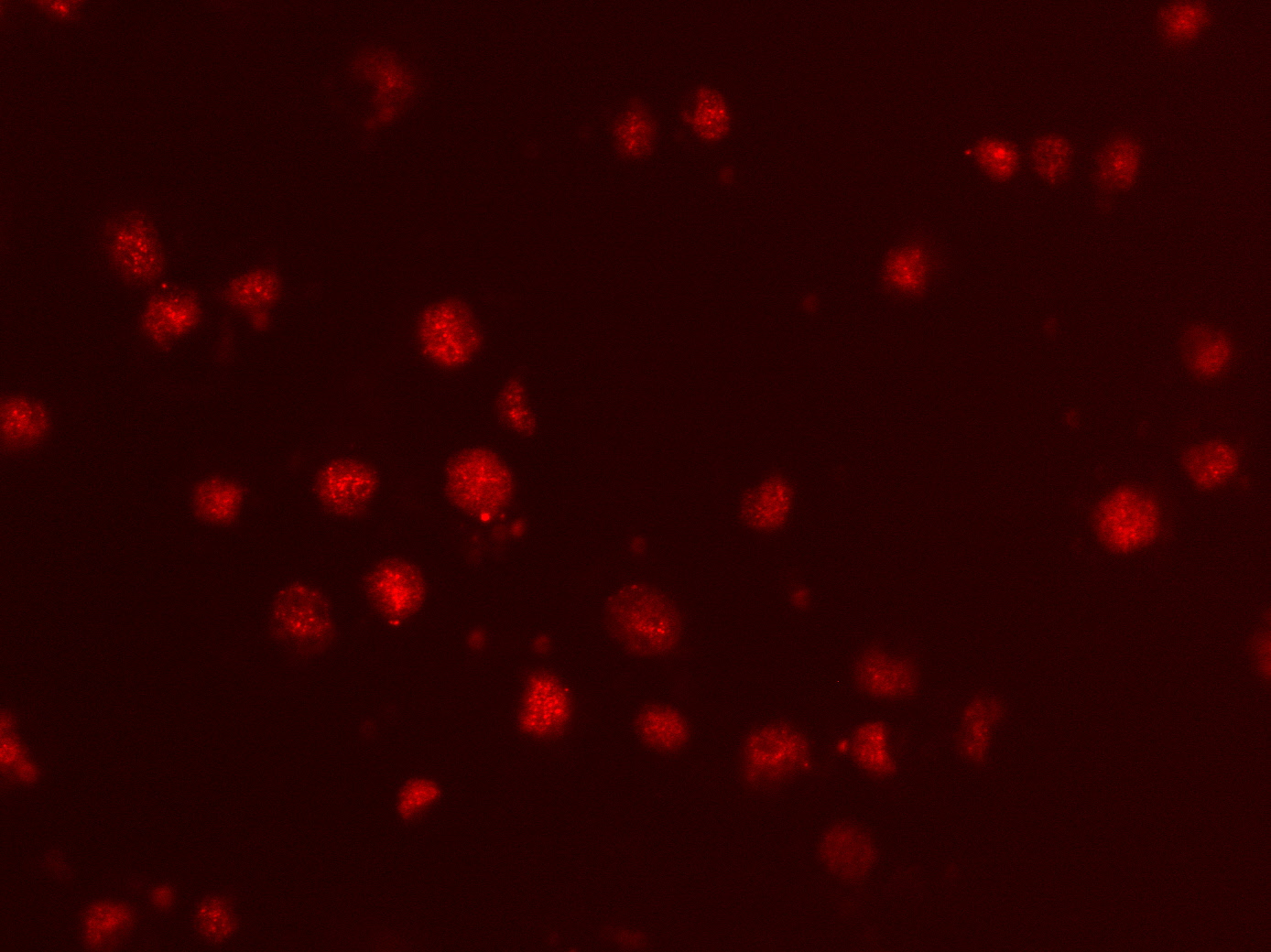

Supplement: S3 File — (ZIP) [file pone.0233739.s003.zip › S3_File/H 460 Res-24h-2012-0026_c2.JPG]

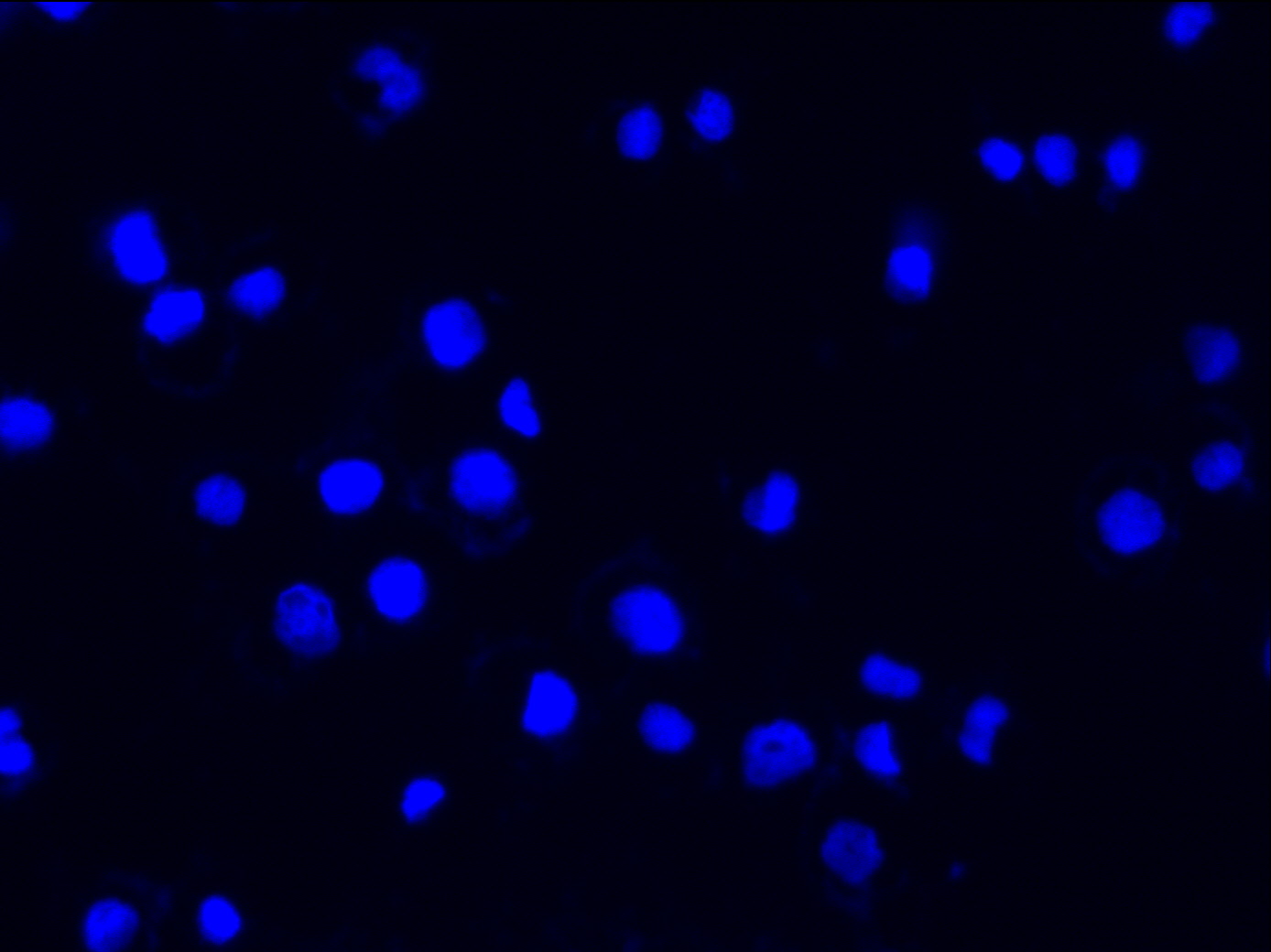

Supplement: S3 File — (ZIP) [file pone.0233739.s003.zip › S3_File/H 460 Res-24h-2012-0026_c3.JPG]

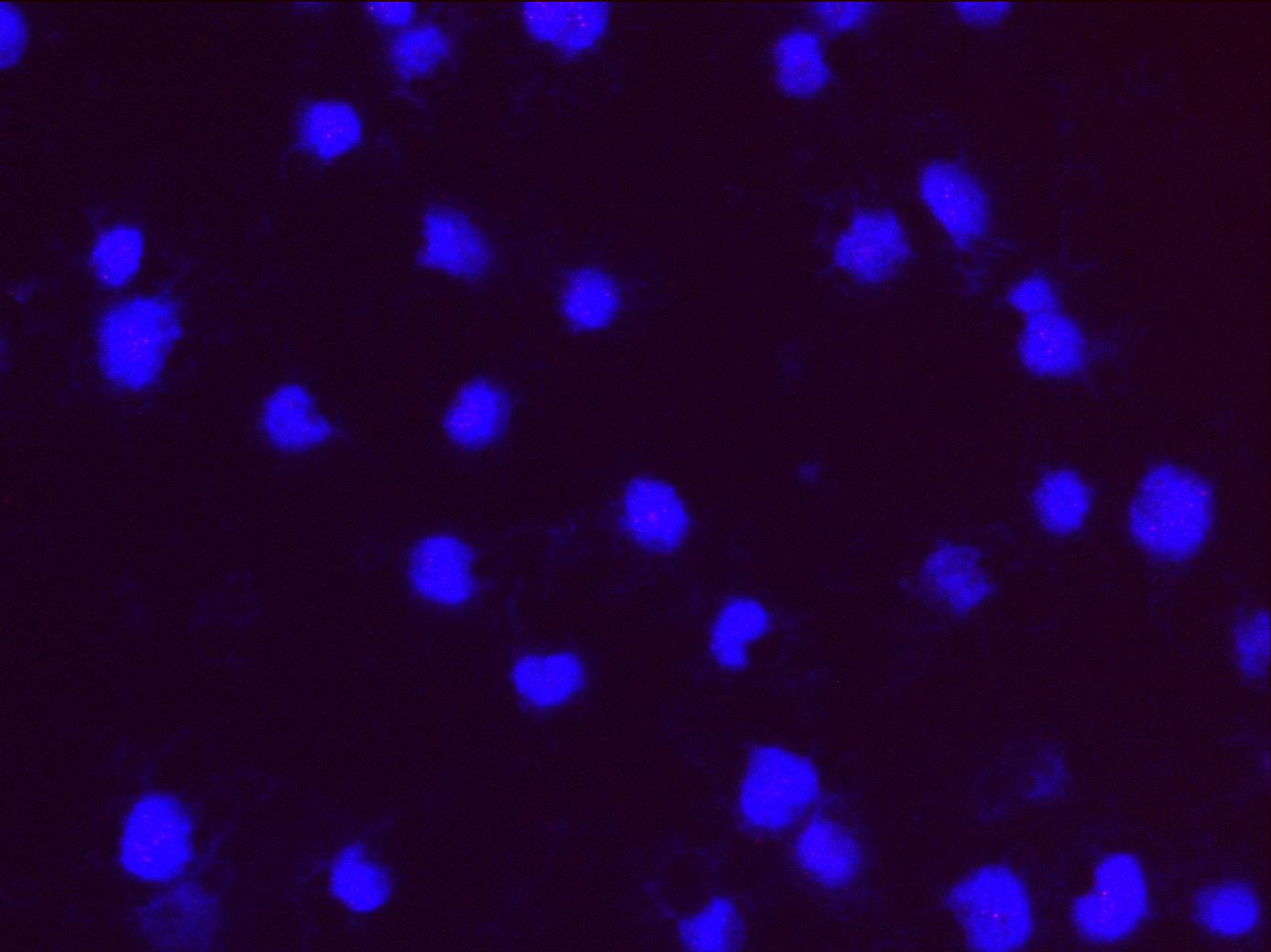

Supplement: S3 File — (ZIP) [file pone.0233739.s003.zip › S3_File/H 460 Res-4h-2012-0024_(c2+c3).JPG]

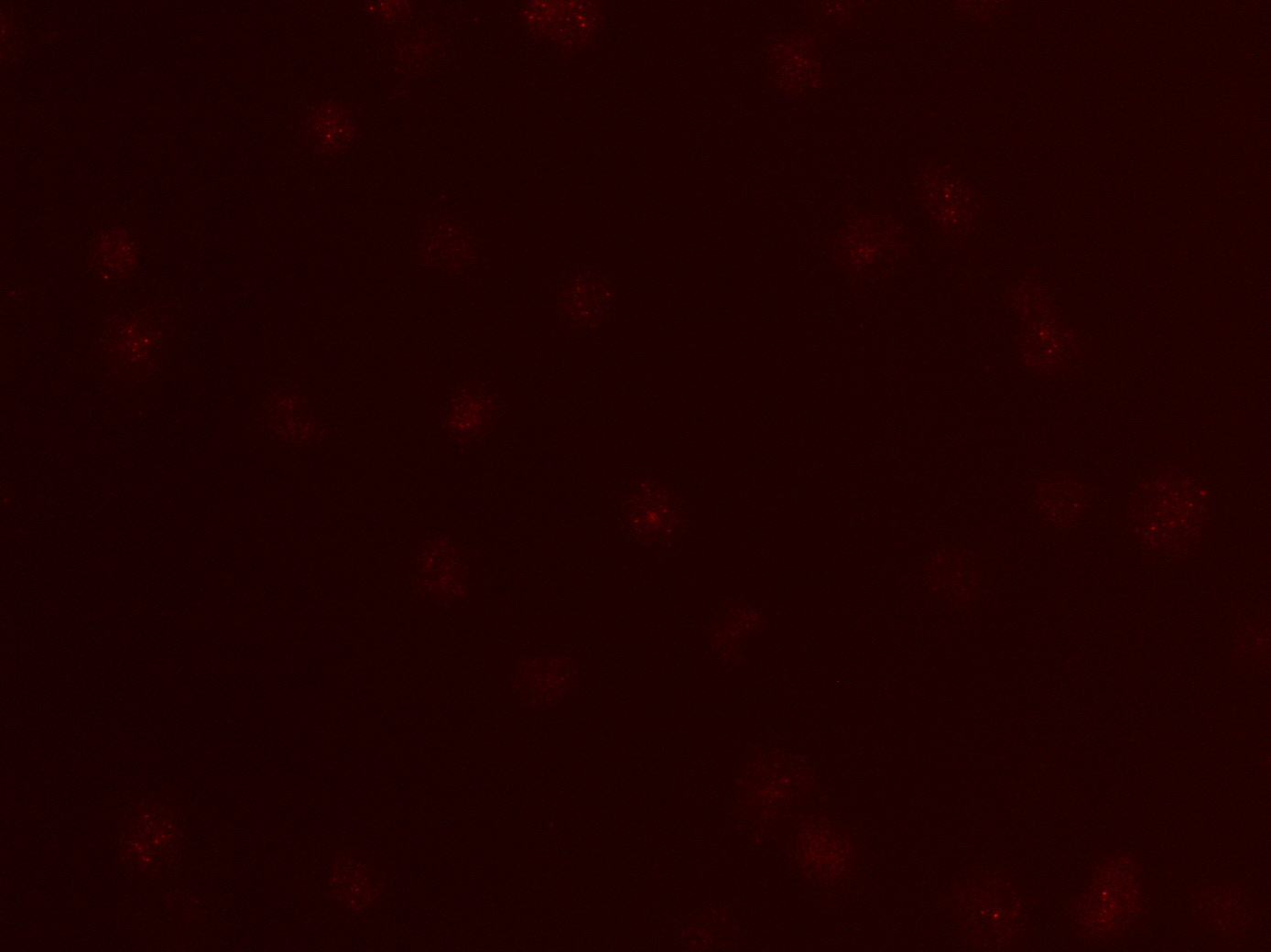

Supplement: S3 File — (ZIP) [file pone.0233739.s003.zip › S3_File/H 460 Res-4h-2012-0024_c2.JPG]

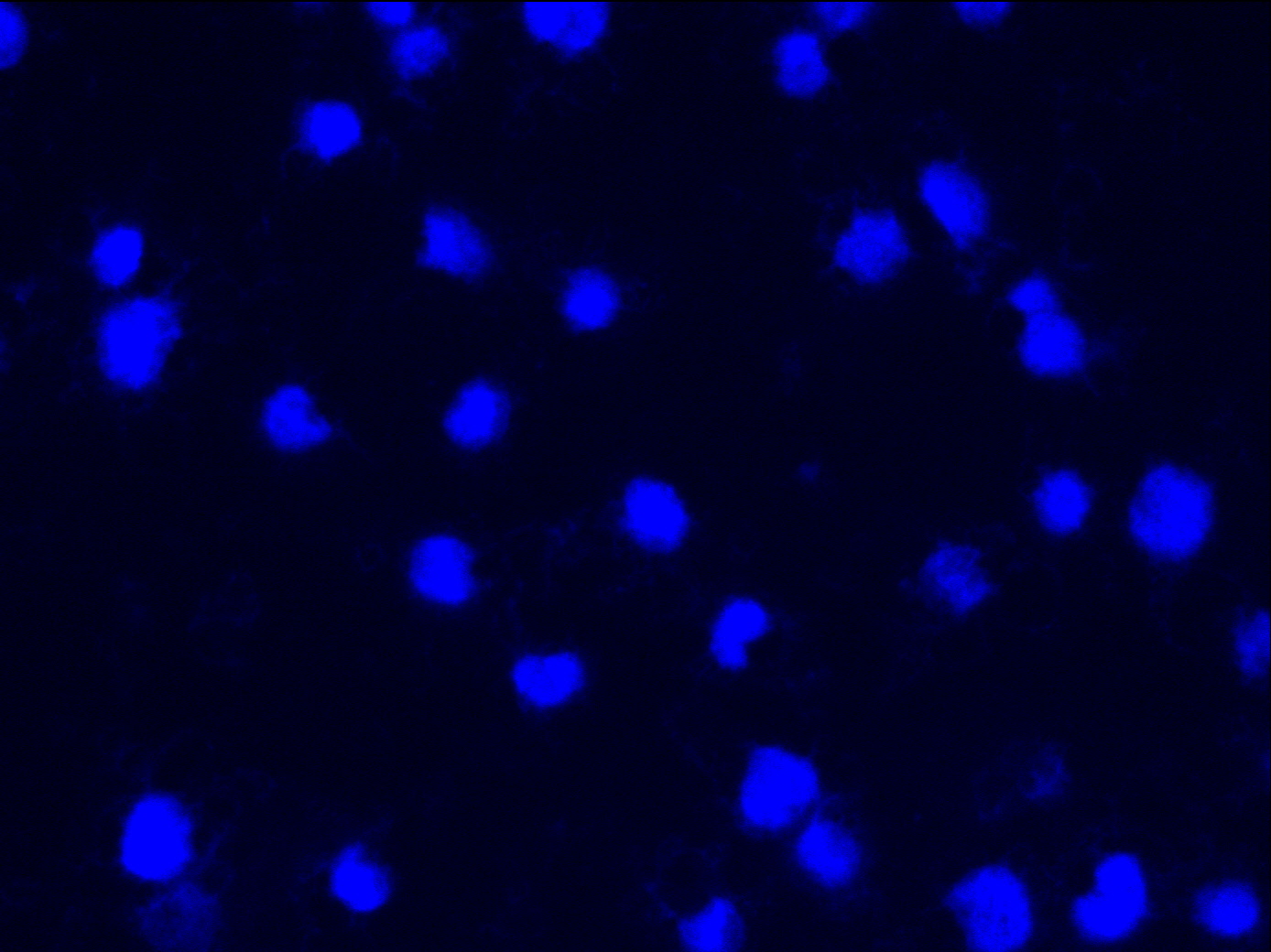

Supplement: S3 File — (ZIP) [file pone.0233739.s003.zip › S3_File/H 460 Res-4h-2012-0024_c3.JPG]

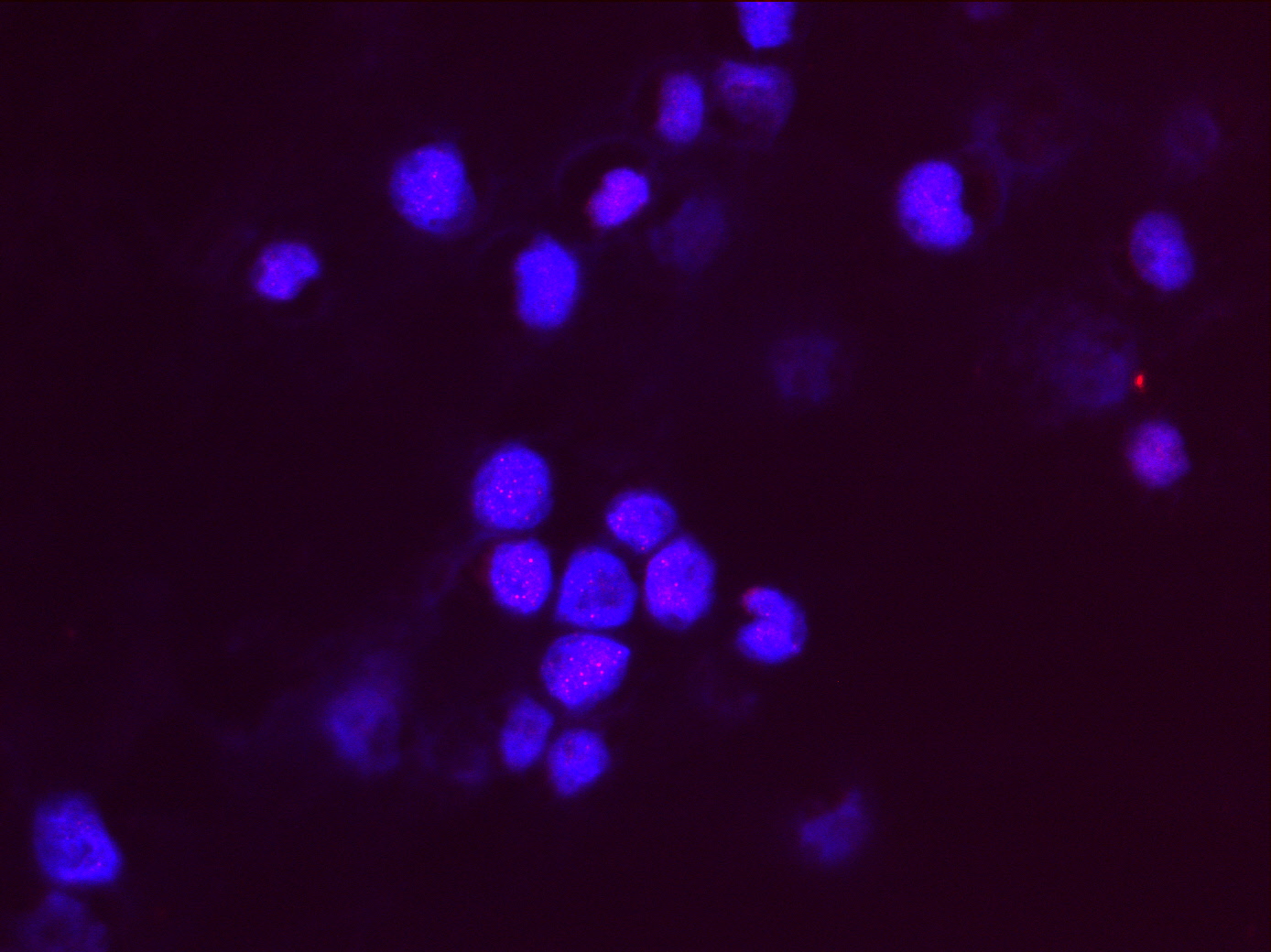

Supplement: S3 File — (ZIP) [file pone.0233739.s003.zip › S3_File/H 460 Sens-12h-2012-0022_(c2+c3).JPG]

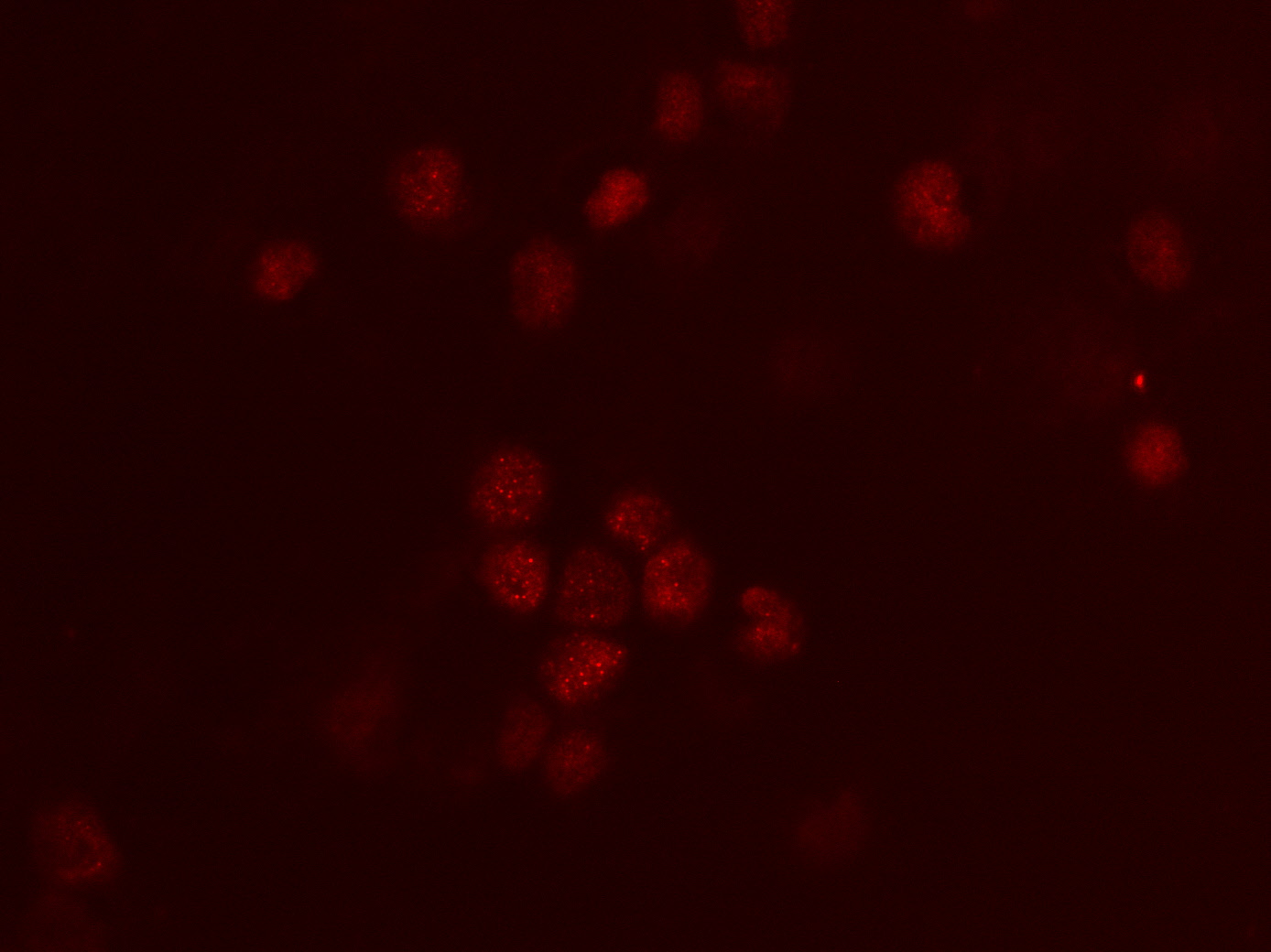

Supplement: S3 File — (ZIP) [file pone.0233739.s003.zip › S3_File/H 460 Sens-12h-2012-0022_c2.JPG]

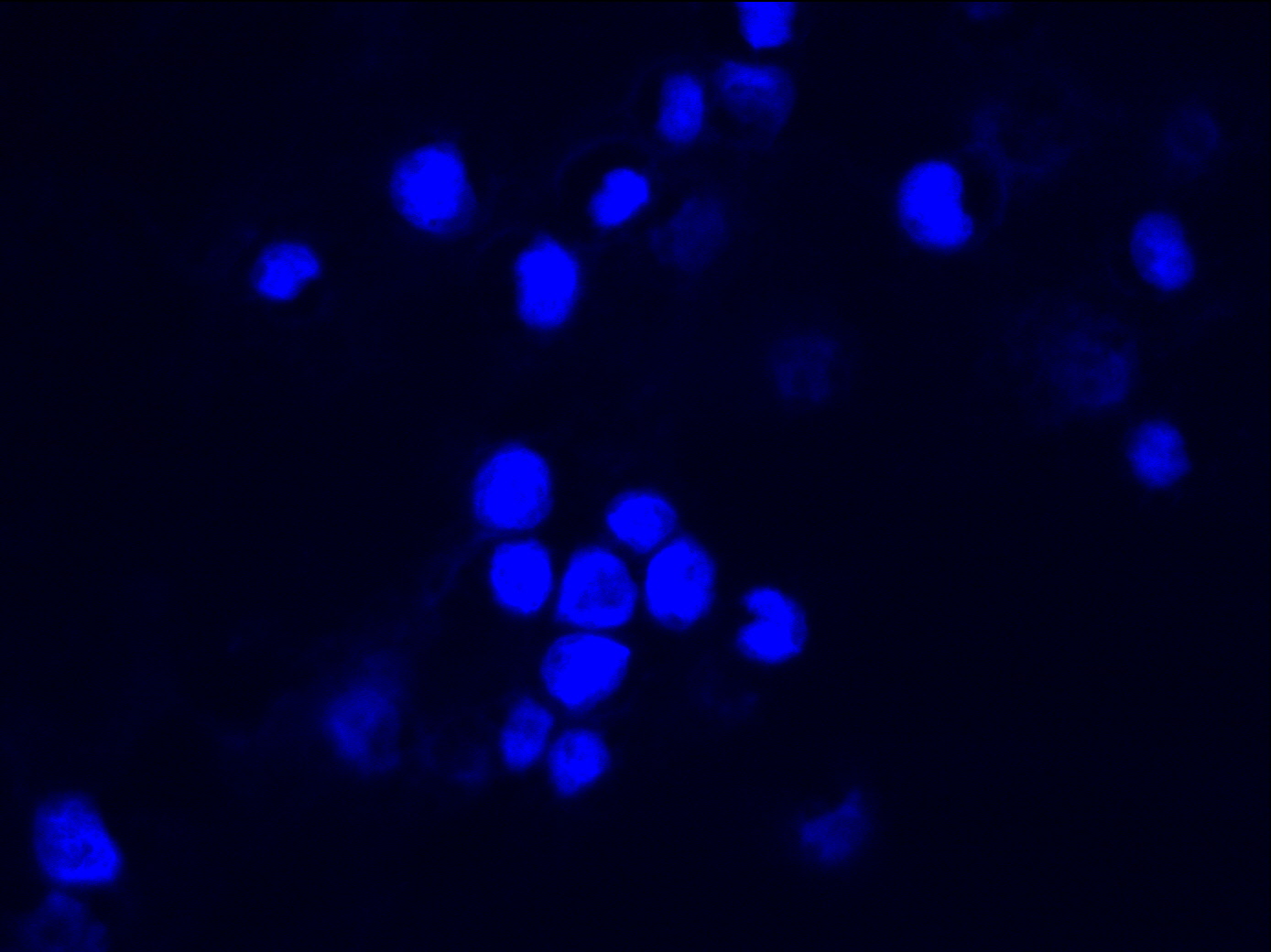

Supplement: S3 File — (ZIP) [file pone.0233739.s003.zip › S3_File/H 460 Sens-12h-2012-0022_c3.JPG]

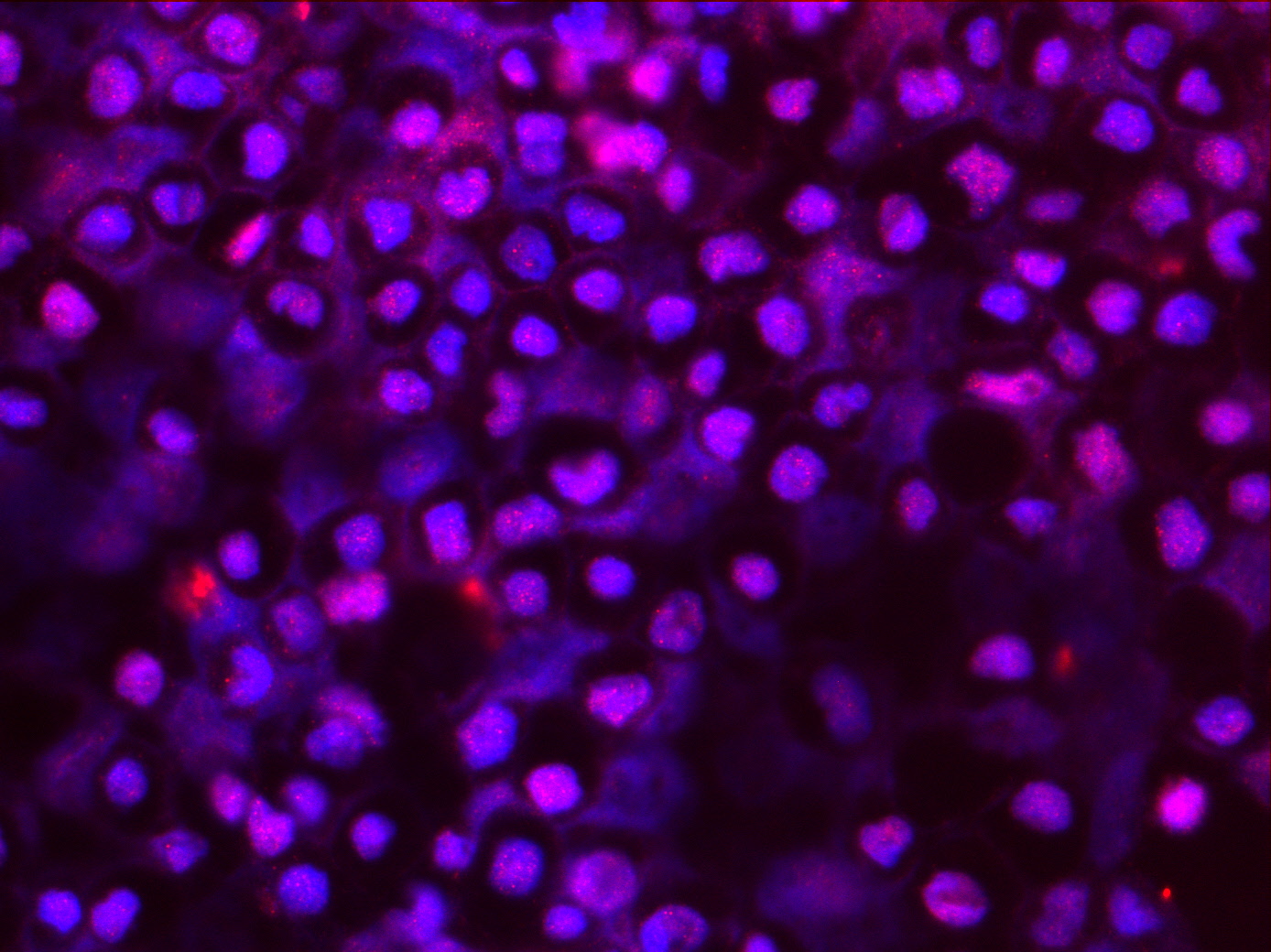

Supplement: S3 File — (ZIP) [file pone.0233739.s003.zip › S3_File/H 460 Sens-24h-2012-0023_(c2+c3).JPG]

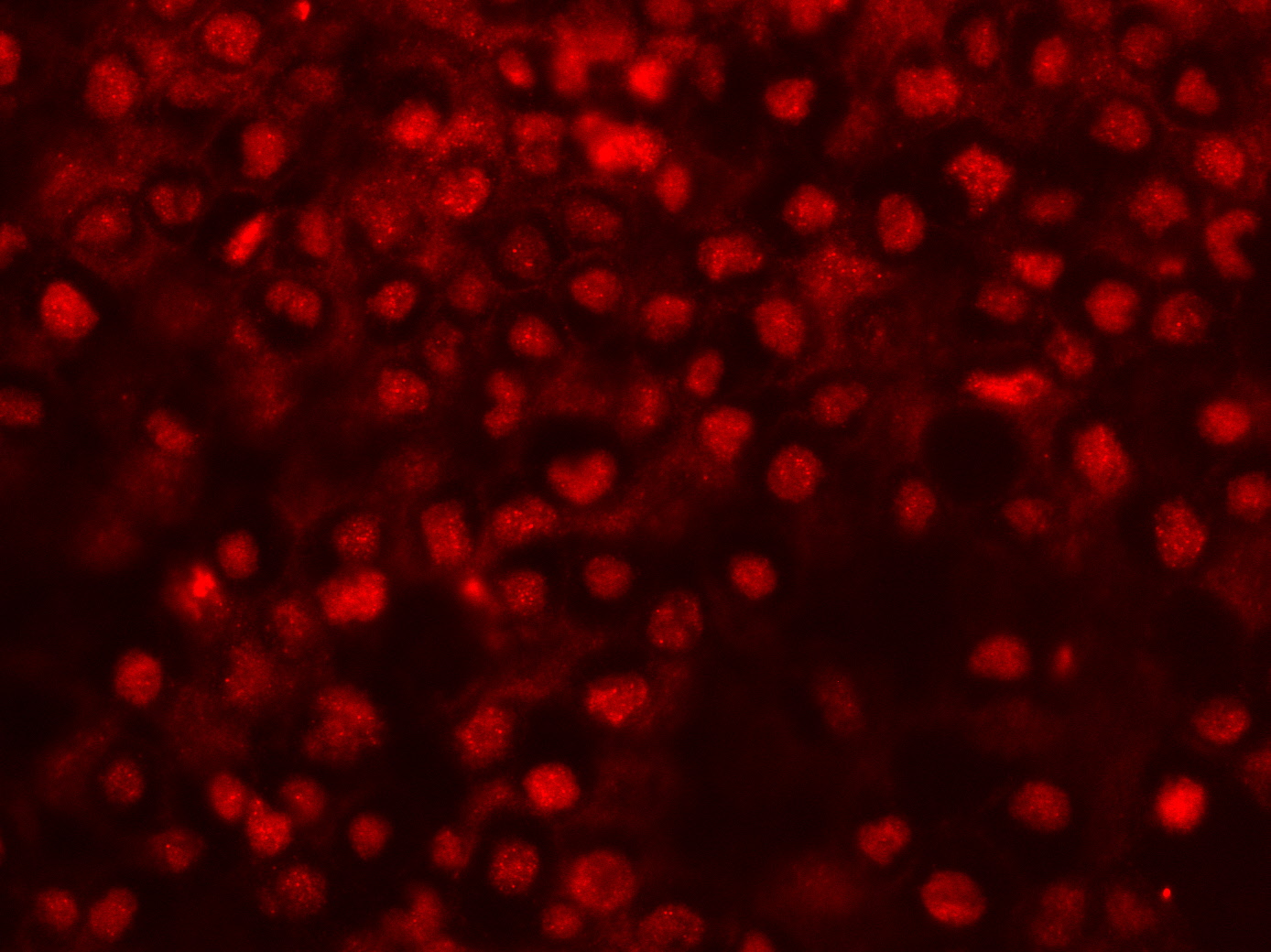

Supplement: S3 File — (ZIP) [file pone.0233739.s003.zip › S3_File/H 460 Sens-24h-2012-0023_c2.JPG]

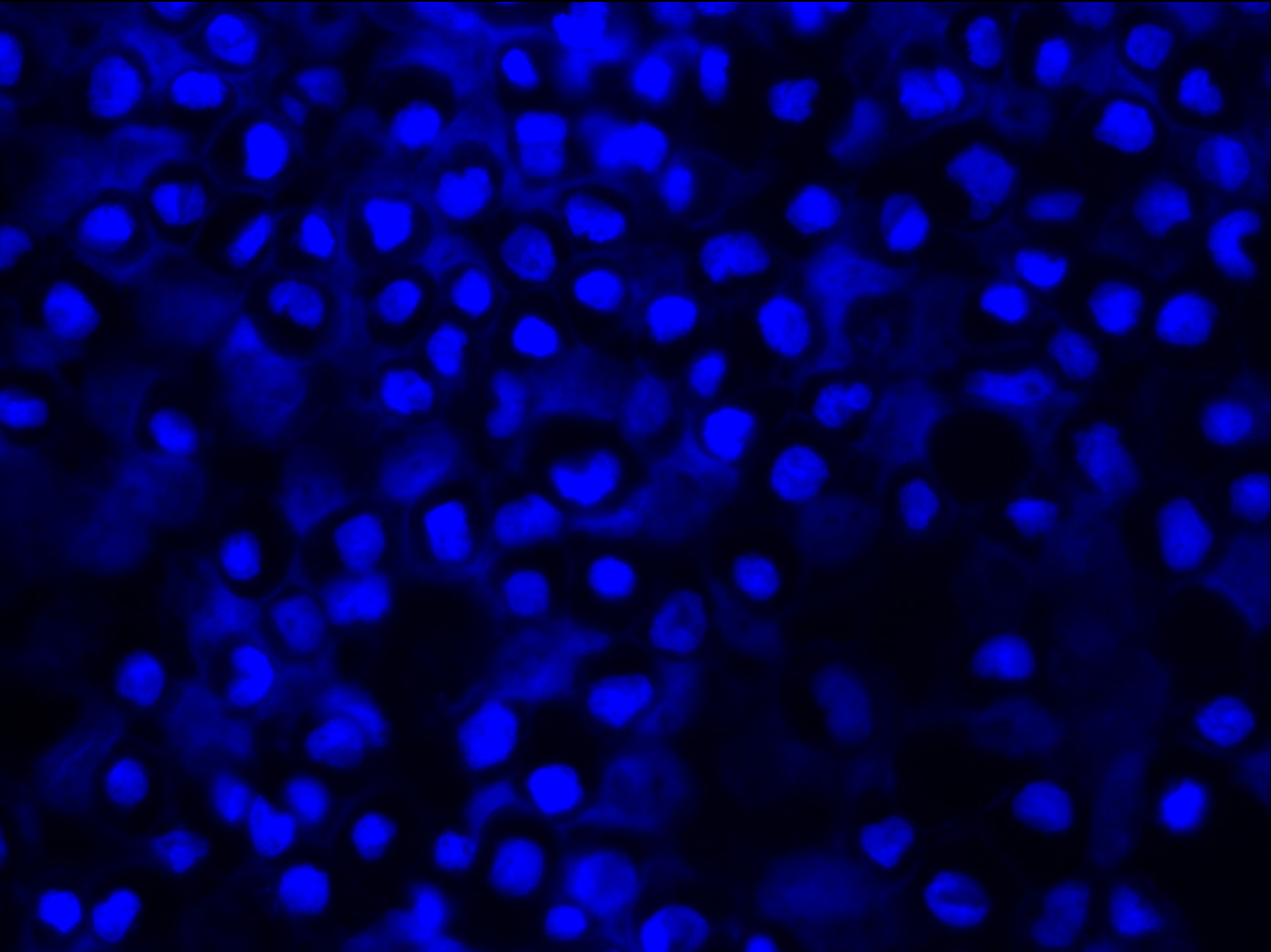

Supplement: S3 File — (ZIP) [file pone.0233739.s003.zip › S3_File/H 460 Sens-24h-2012-0023_c3.JPG]

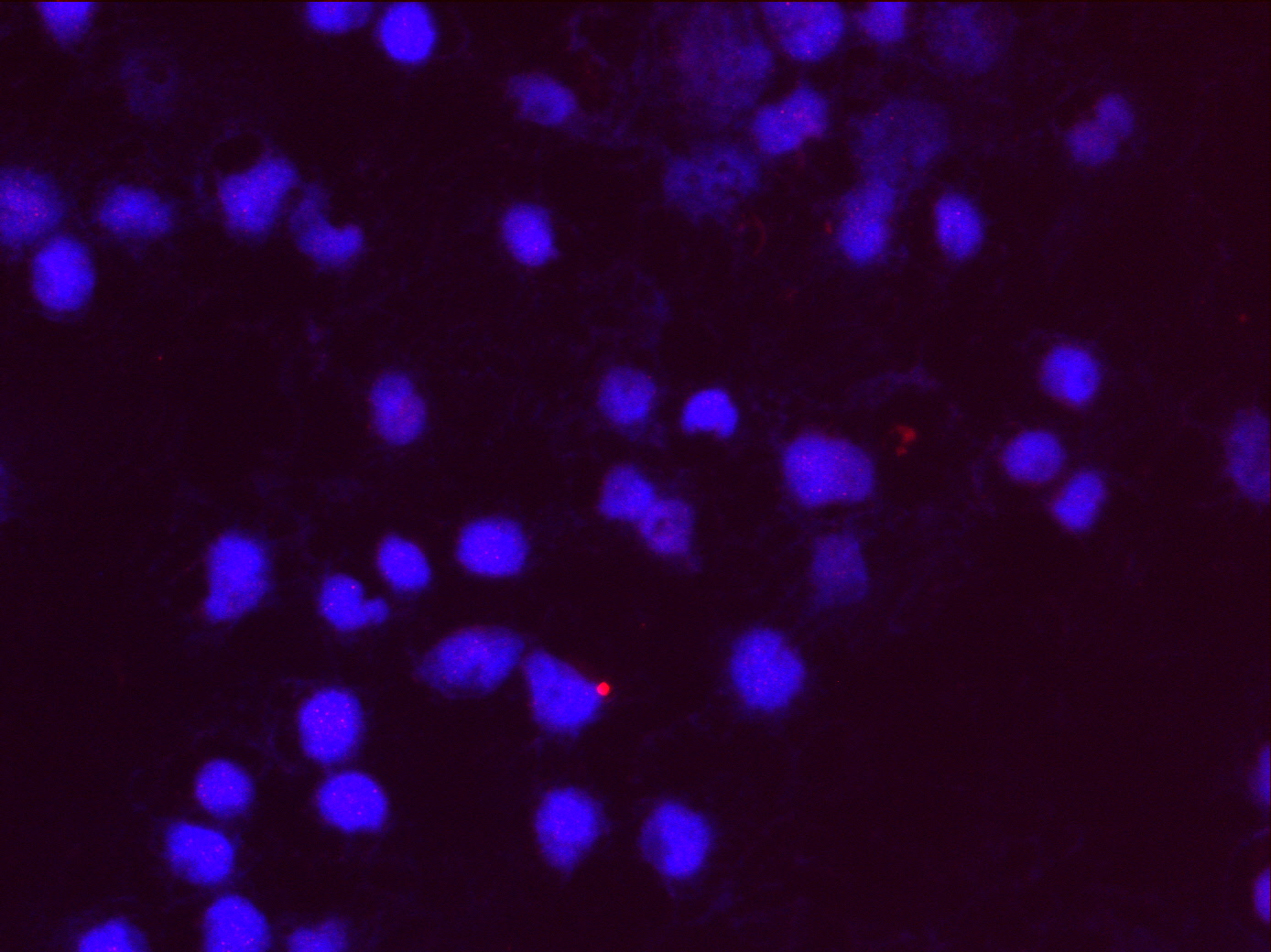

Supplement: S3 File — (ZIP) [file pone.0233739.s003.zip › S3_File/H 460 Sens-4h-2012-0020_(c2+c3).JPG]

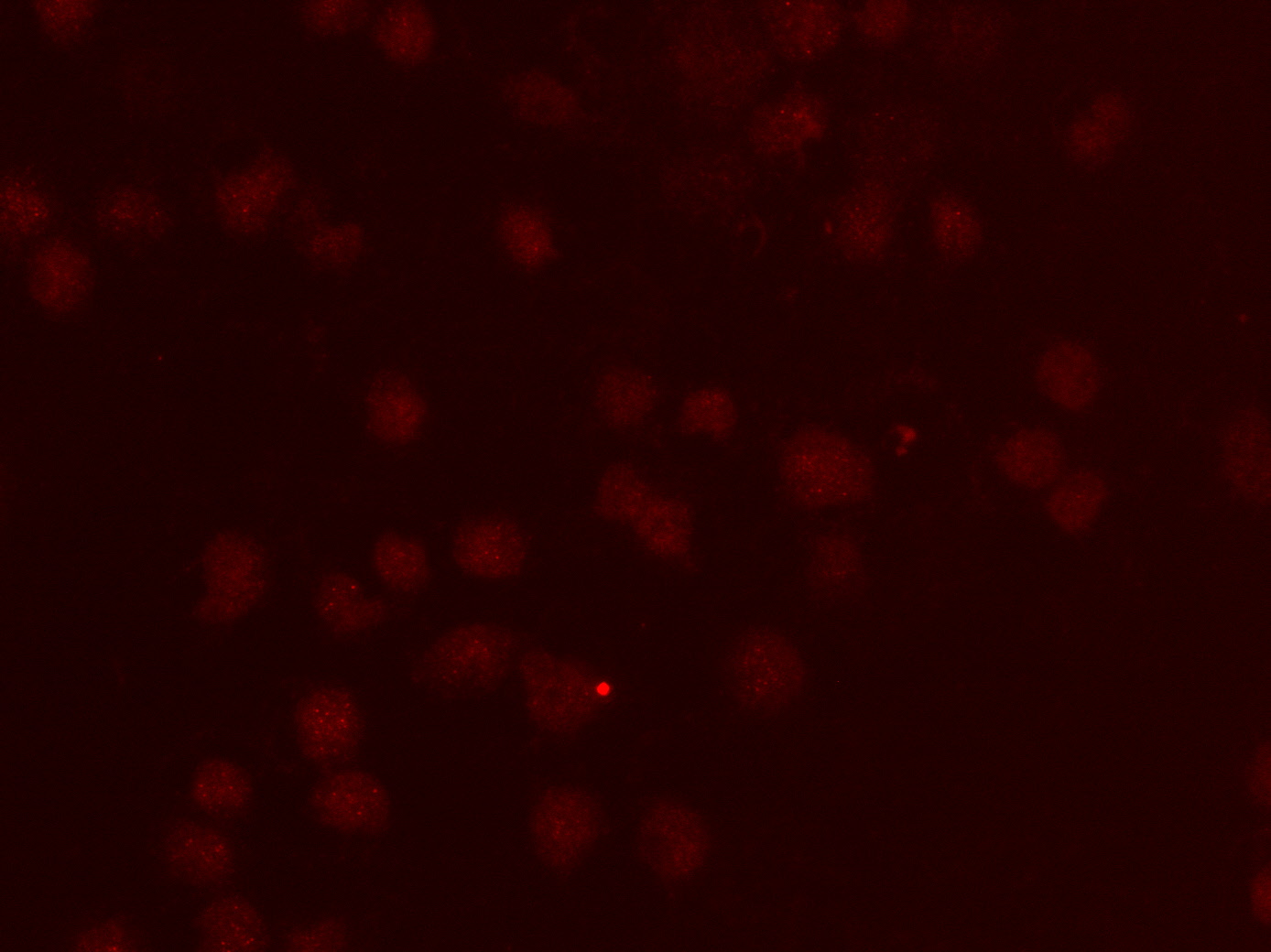

Supplement: S3 File — (ZIP) [file pone.0233739.s003.zip › S3_File/H 460 Sens-4h-2012-0020_c2.JPG]

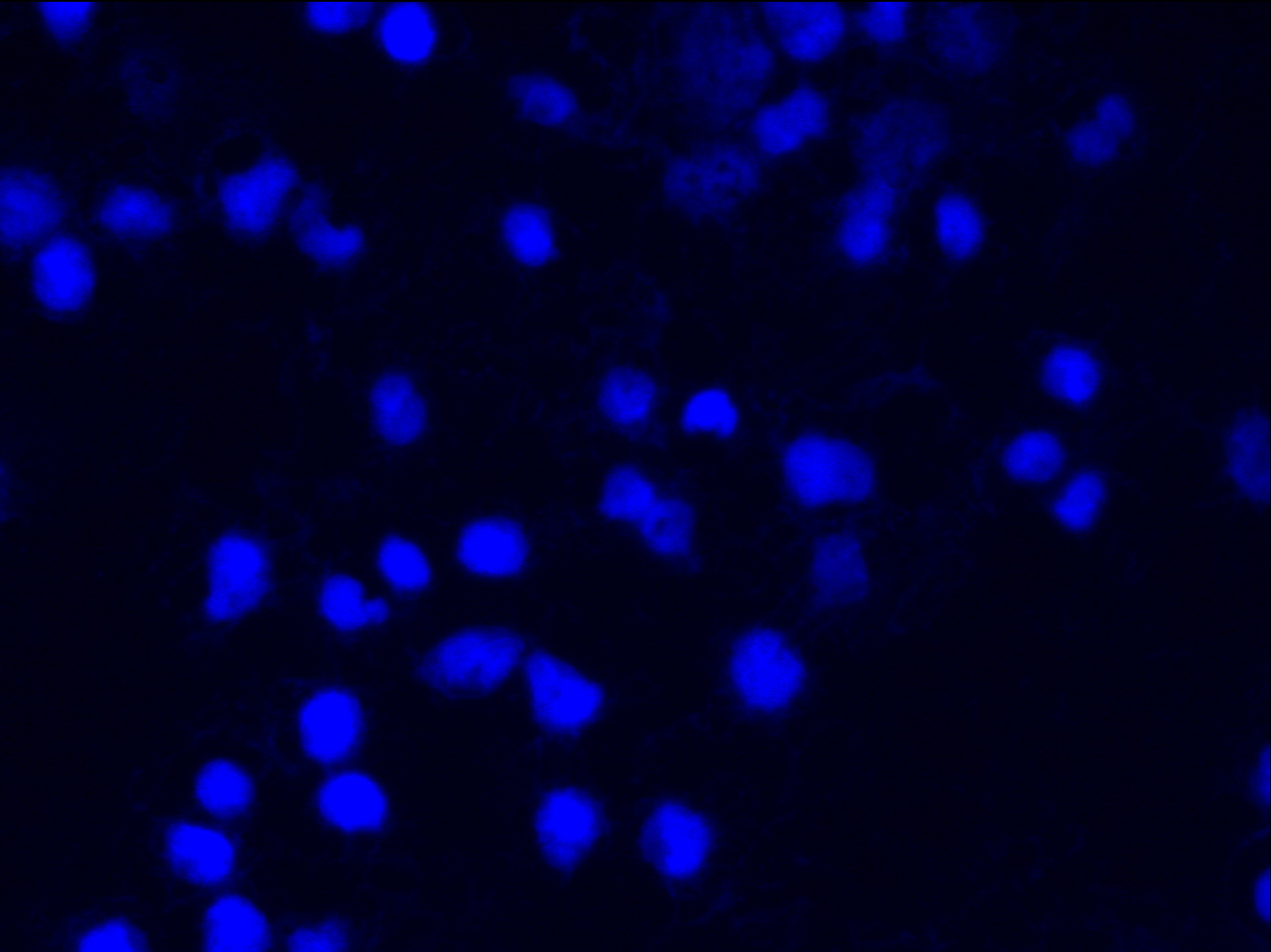

Supplement: S3 File — (ZIP) [file pone.0233739.s003.zip › S3_File/H 460 Sens-4h-2012-0020_c3.JPG]

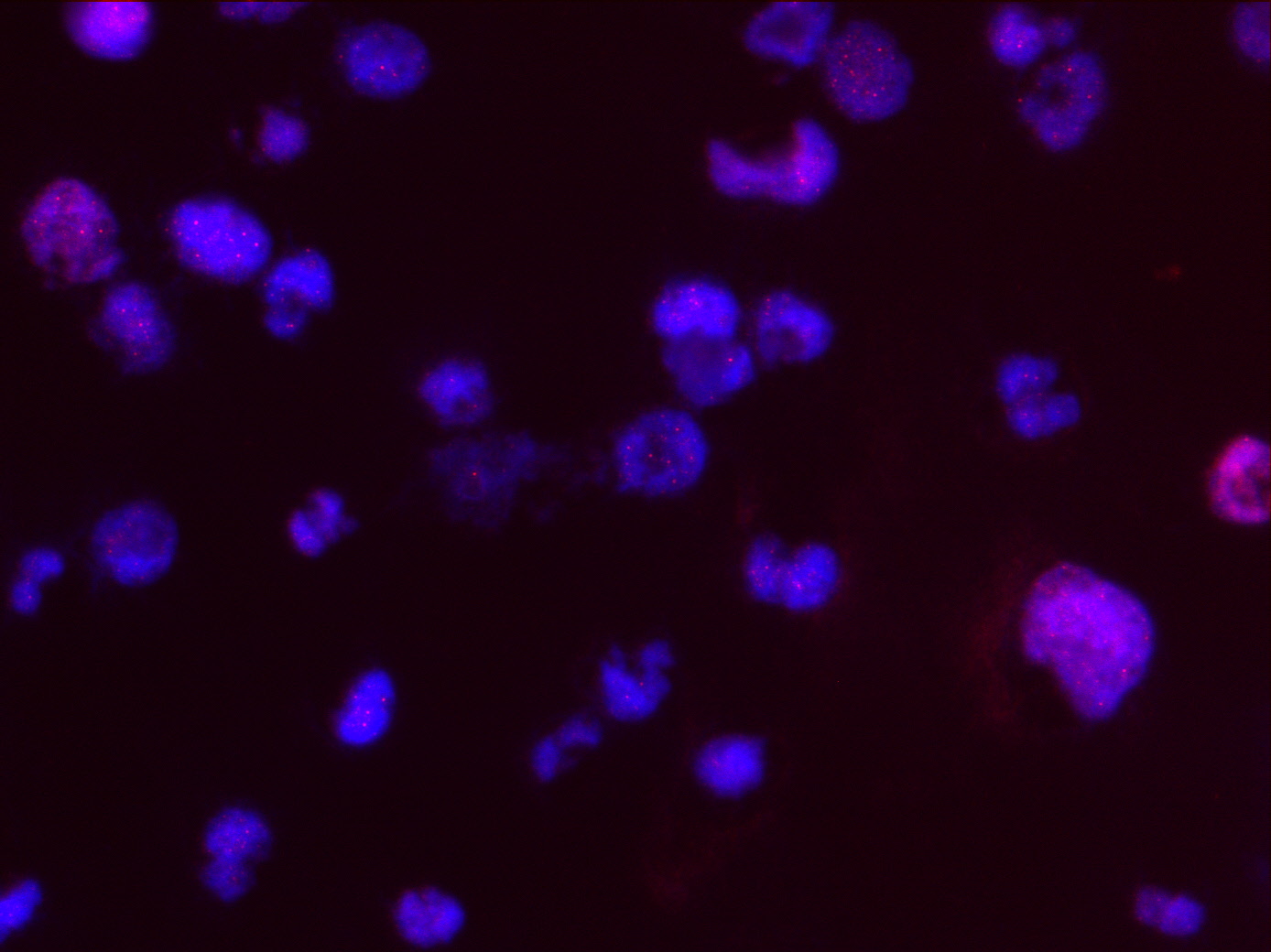

Supplement: S3 File — (ZIP) [file pone.0233739.s003.zip › S3_File/HI 299 Res-4h-2012-0017_(c2+c3).JPG]

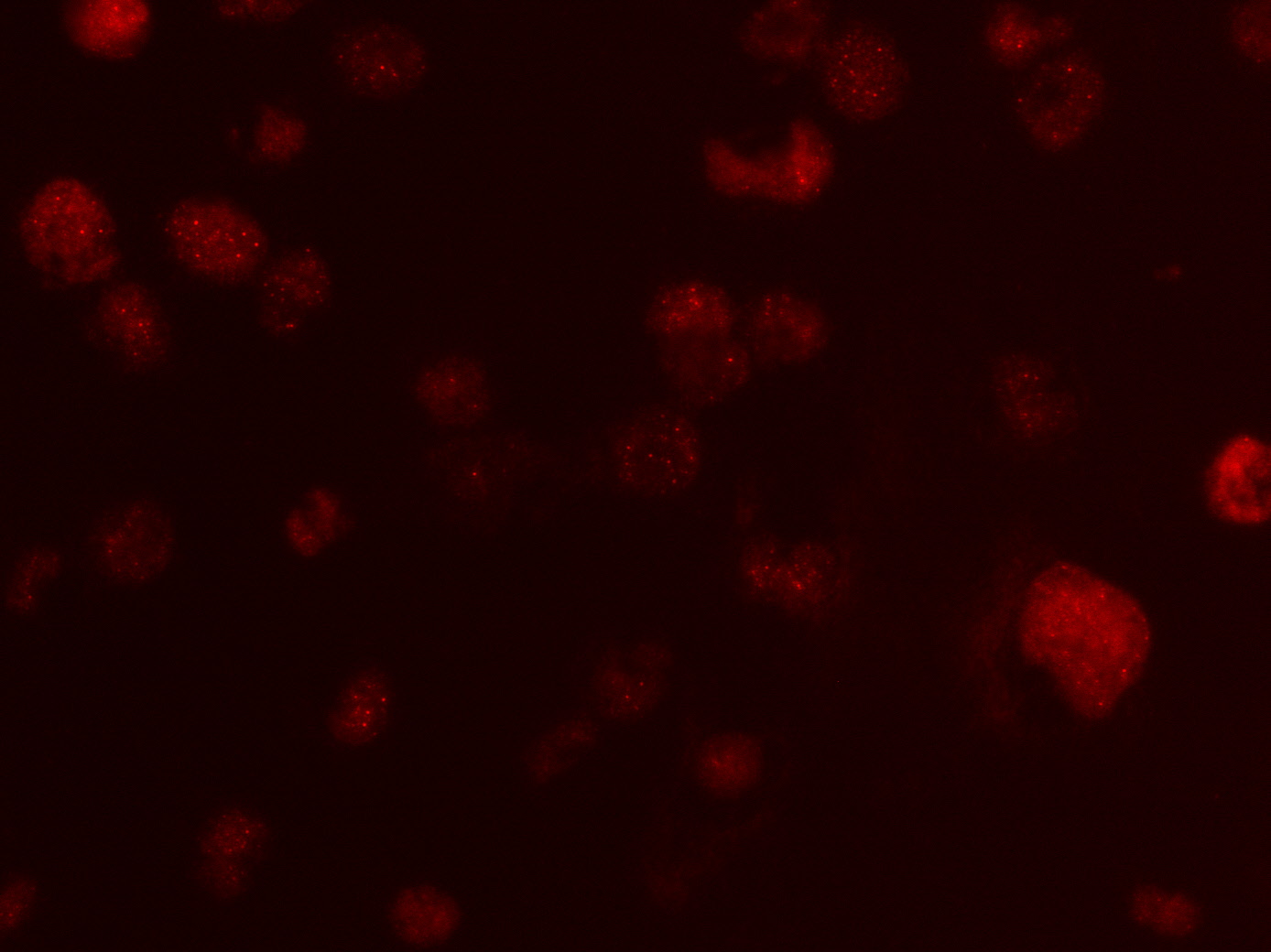

Supplement: S3 File — (ZIP) [file pone.0233739.s003.zip › S3_File/HI 299 Res-4h-2012-0017_c2.JPG]

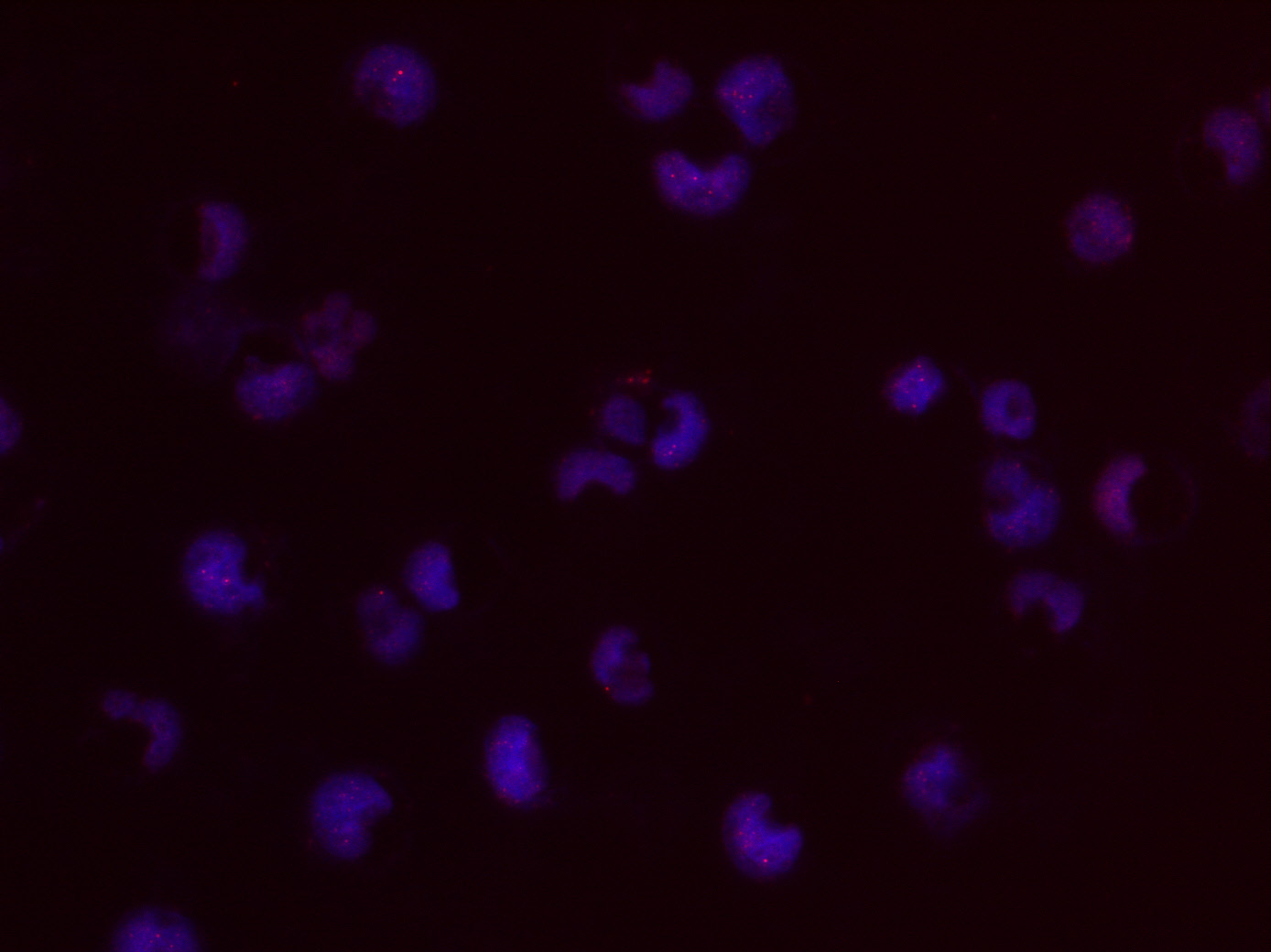

Supplement: S4 File — (ZIP) [file pone.0233739.s004.zip › S4_File/A549 Res-12h-2012-0012_(c2+c3).JPG]

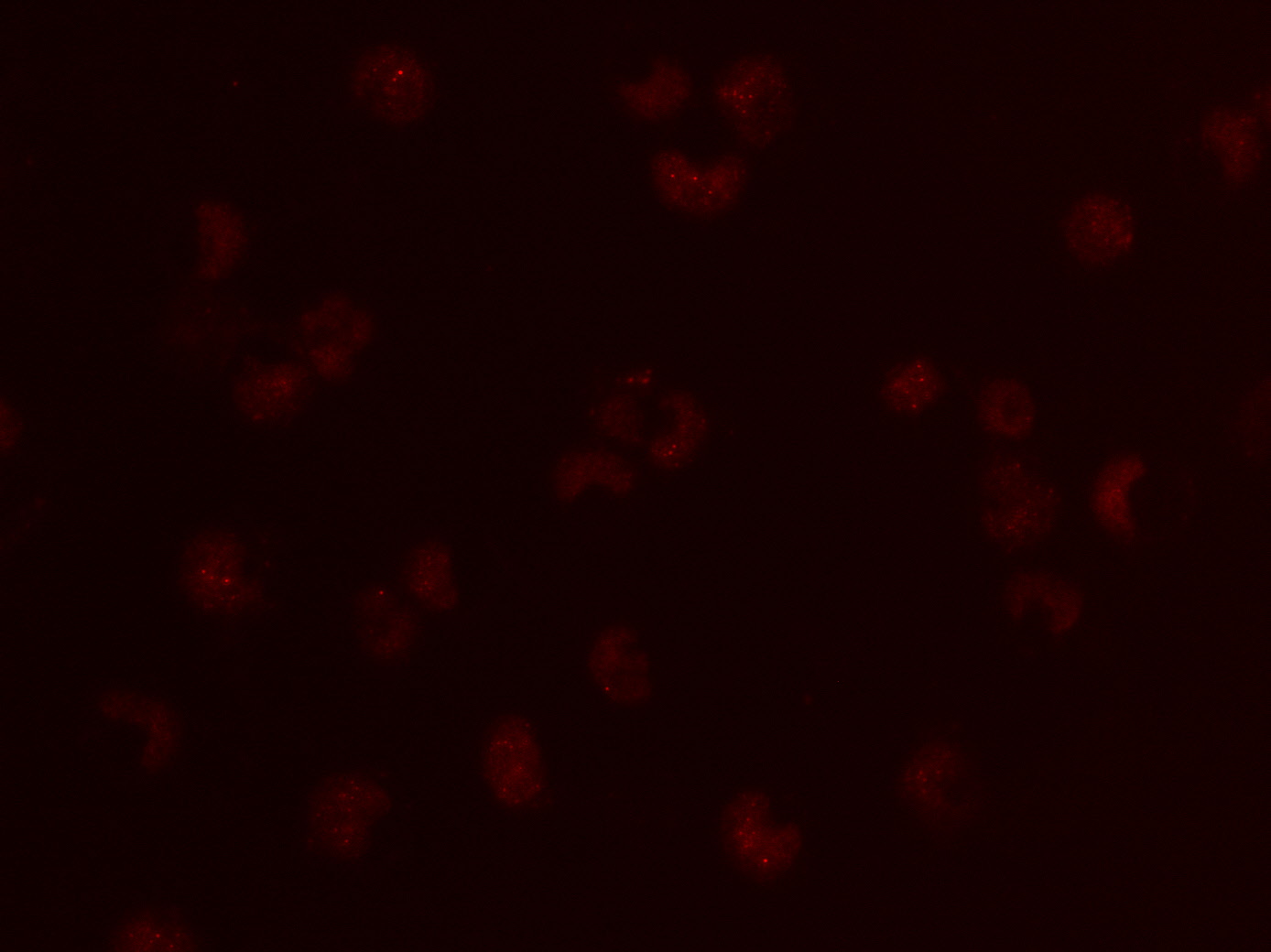

Supplement: S4 File — (ZIP) [file pone.0233739.s004.zip › S4_File/A549 Res-12h-2012-0012_c2.JPG]

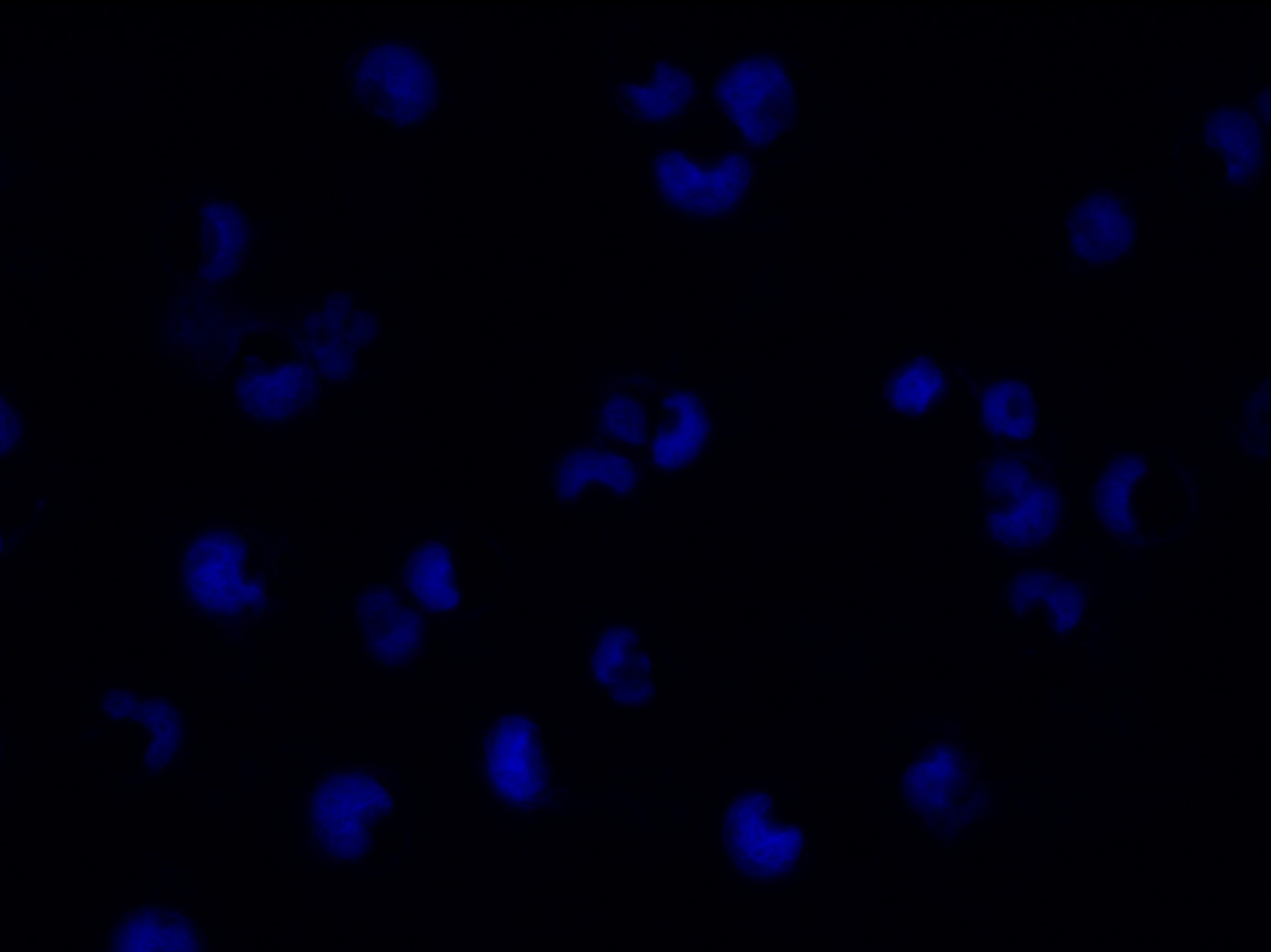

Supplement: S4 File — (ZIP) [file pone.0233739.s004.zip › S4_File/A549 Res-12h-2012-0012_c3.JPG]

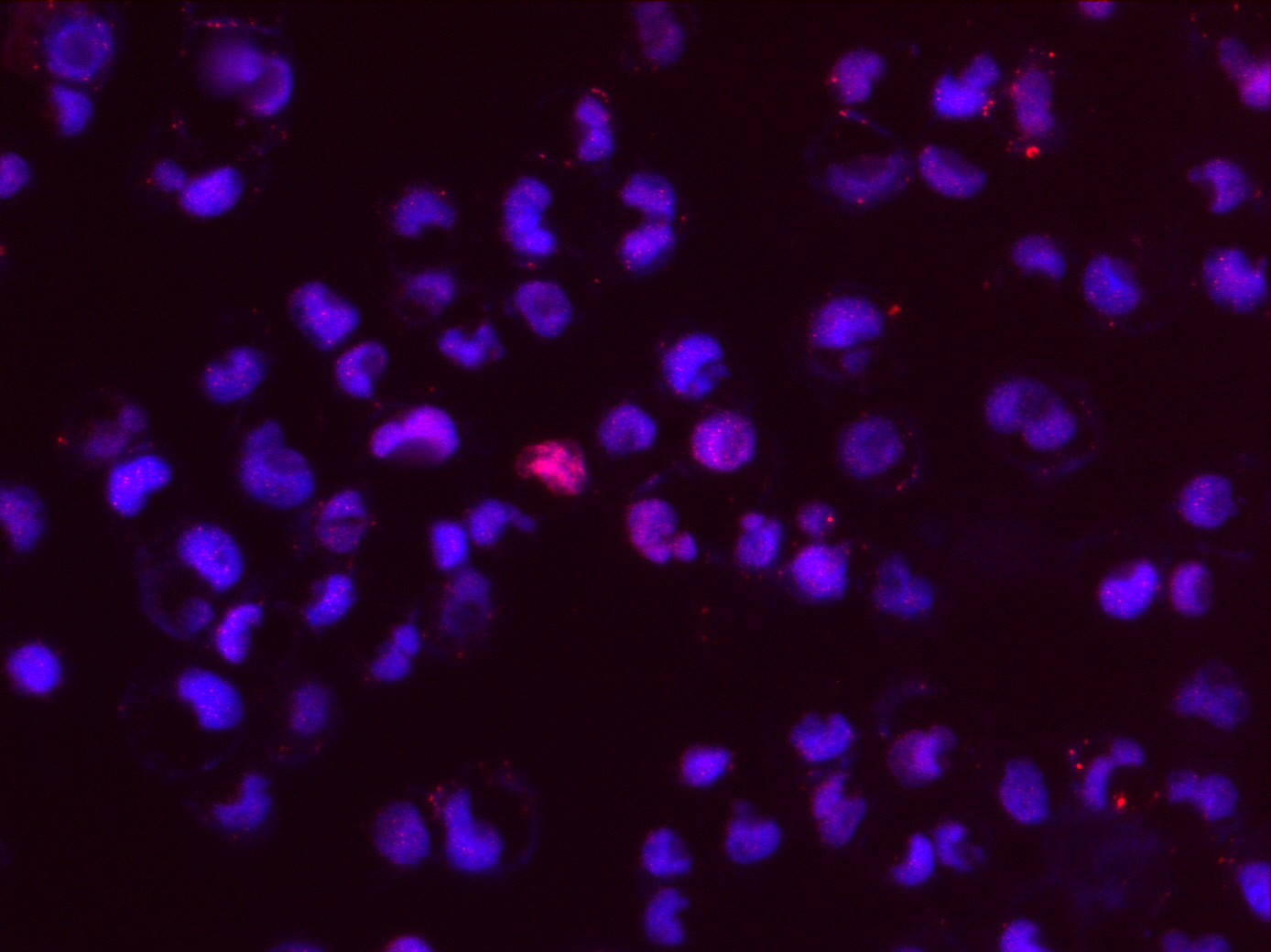

Supplement: S4 File — (ZIP) [file pone.0233739.s004.zip › S4_File/A549 Res-24h-2012-0013_(c2+c3).JPG]

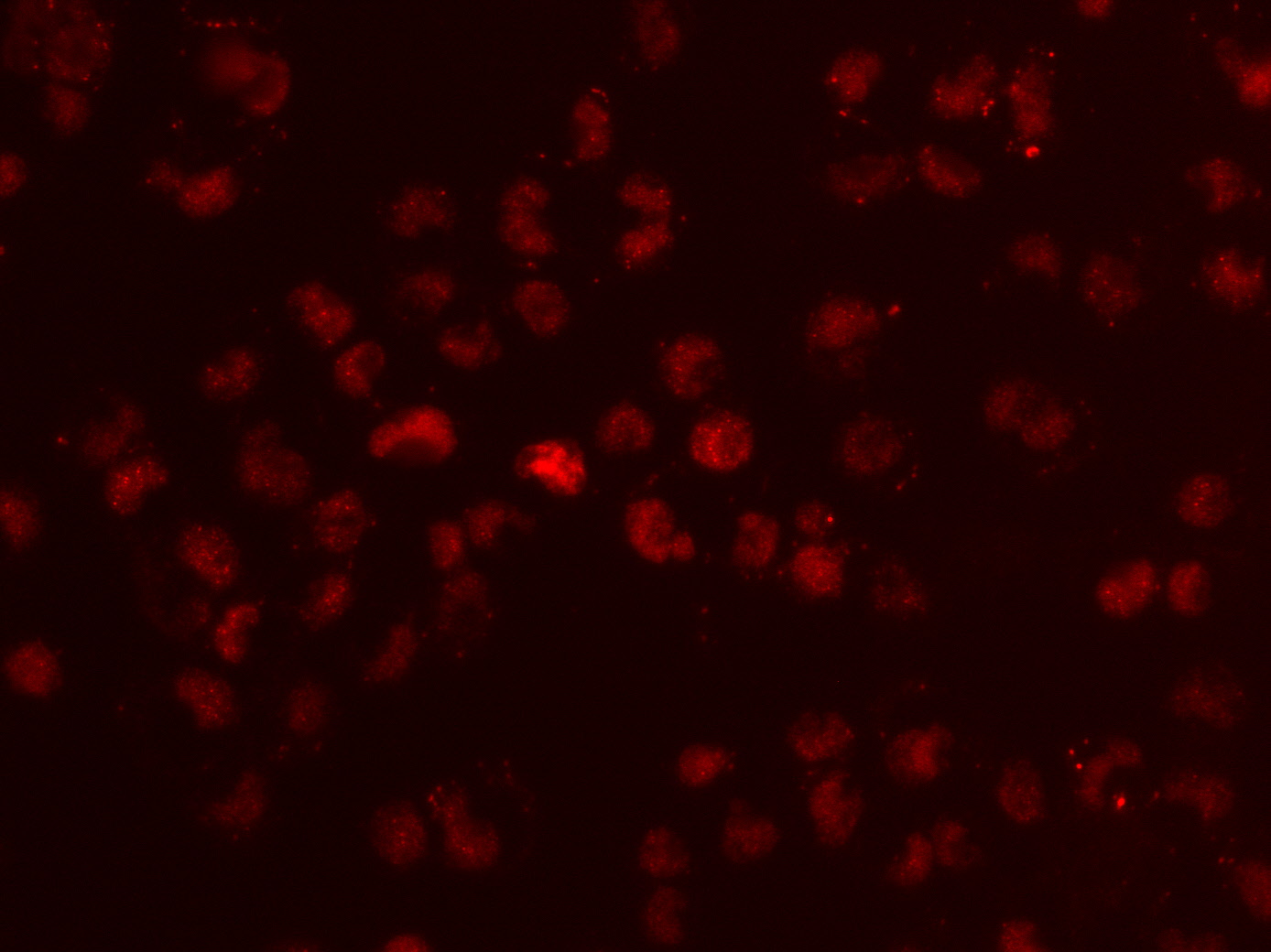

Supplement: S4 File — (ZIP) [file pone.0233739.s004.zip › S4_File/A549 Res-24h-2012-0013_c2.JPG]

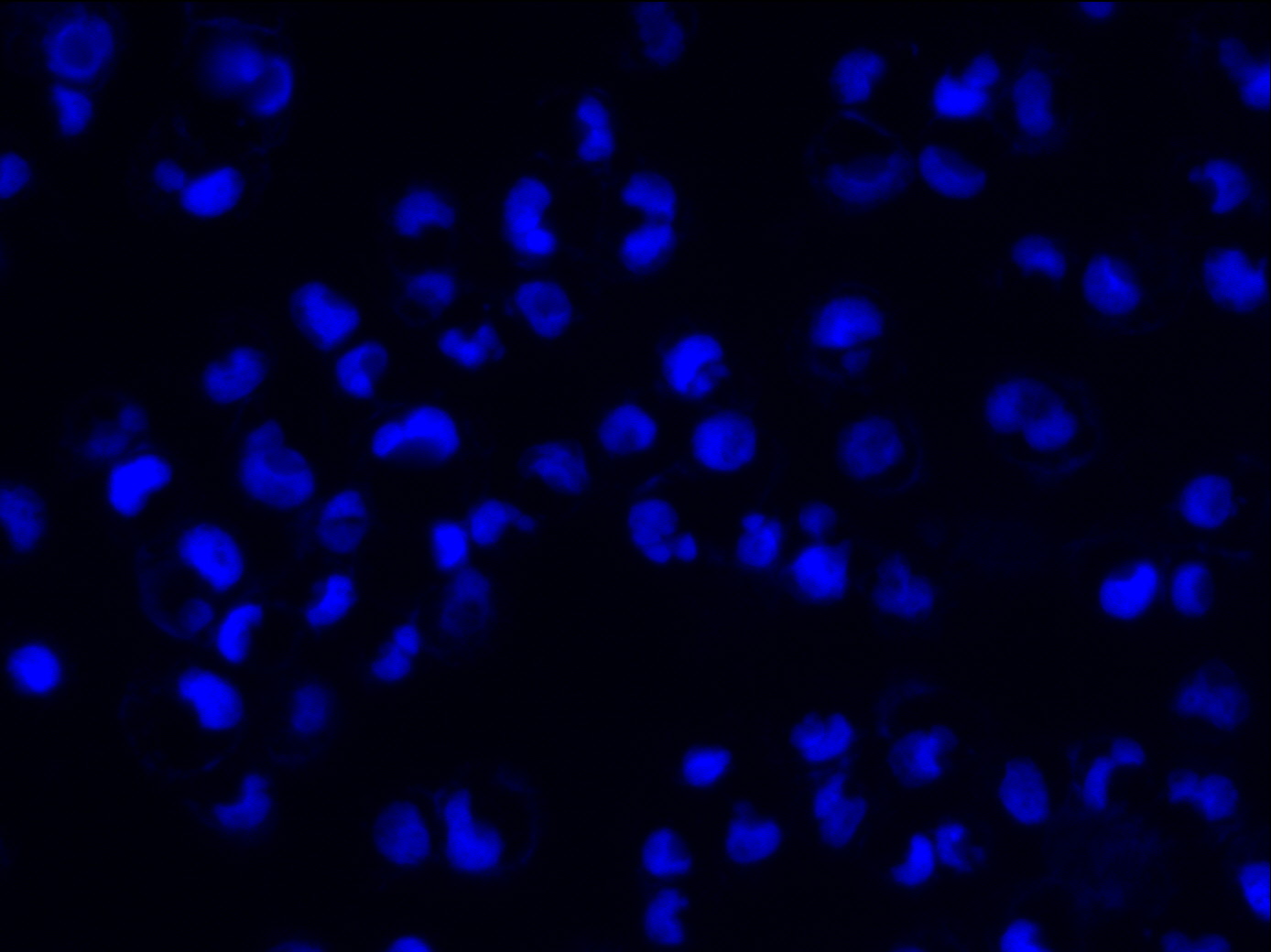

Supplement: S4 File — (ZIP) [file pone.0233739.s004.zip › S4_File/A549 Res-24h-2012-0013_c3.JPG]

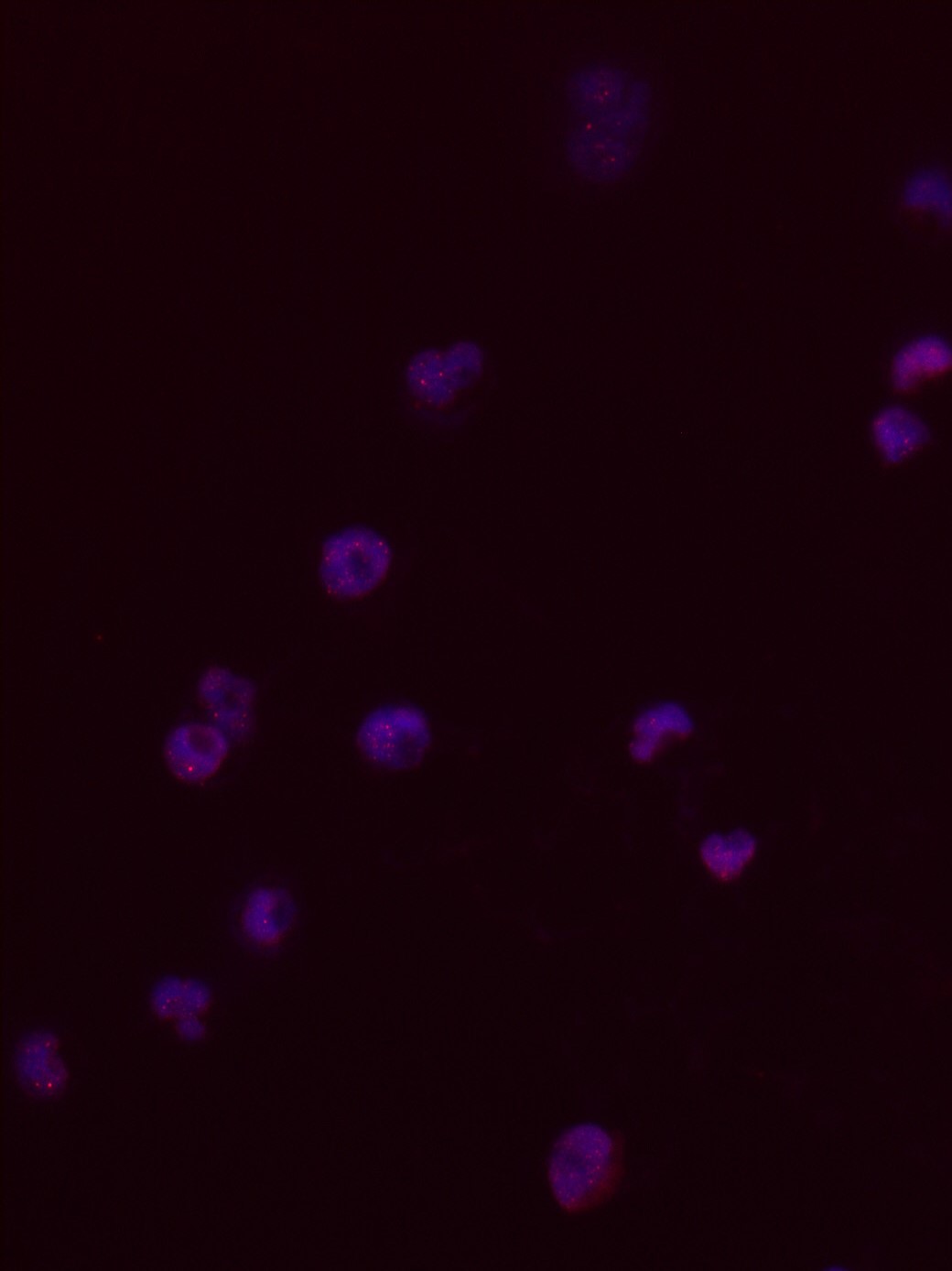

Supplement: S4 File — (ZIP) [file pone.0233739.s004.zip › S4_File/A549 Res-4h-2012-0011_(c2+c3).JPG]

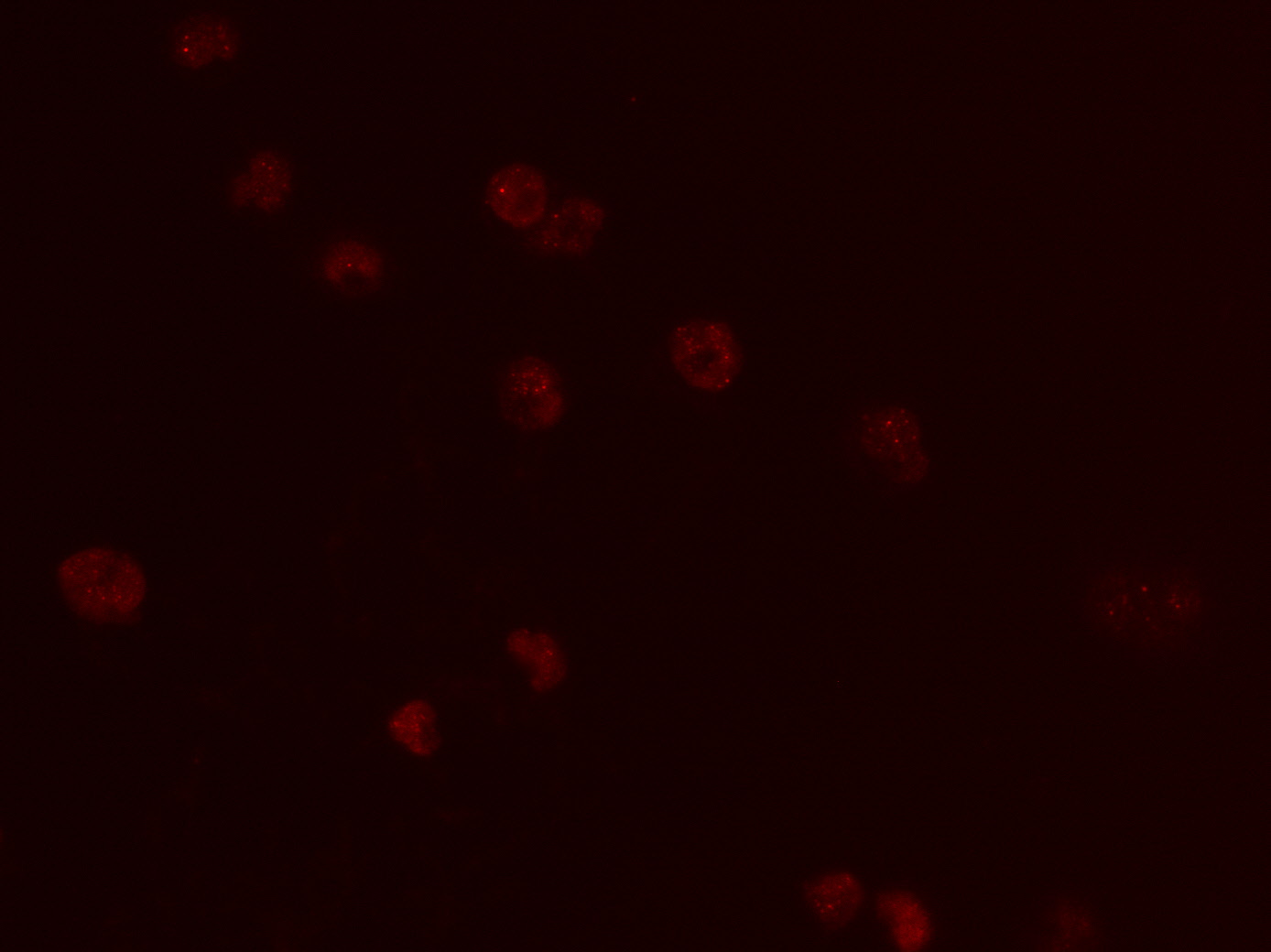

Supplement: S4 File — (ZIP) [file pone.0233739.s004.zip › S4_File/A549 Res-4h-2012-0011_c2.JPG]

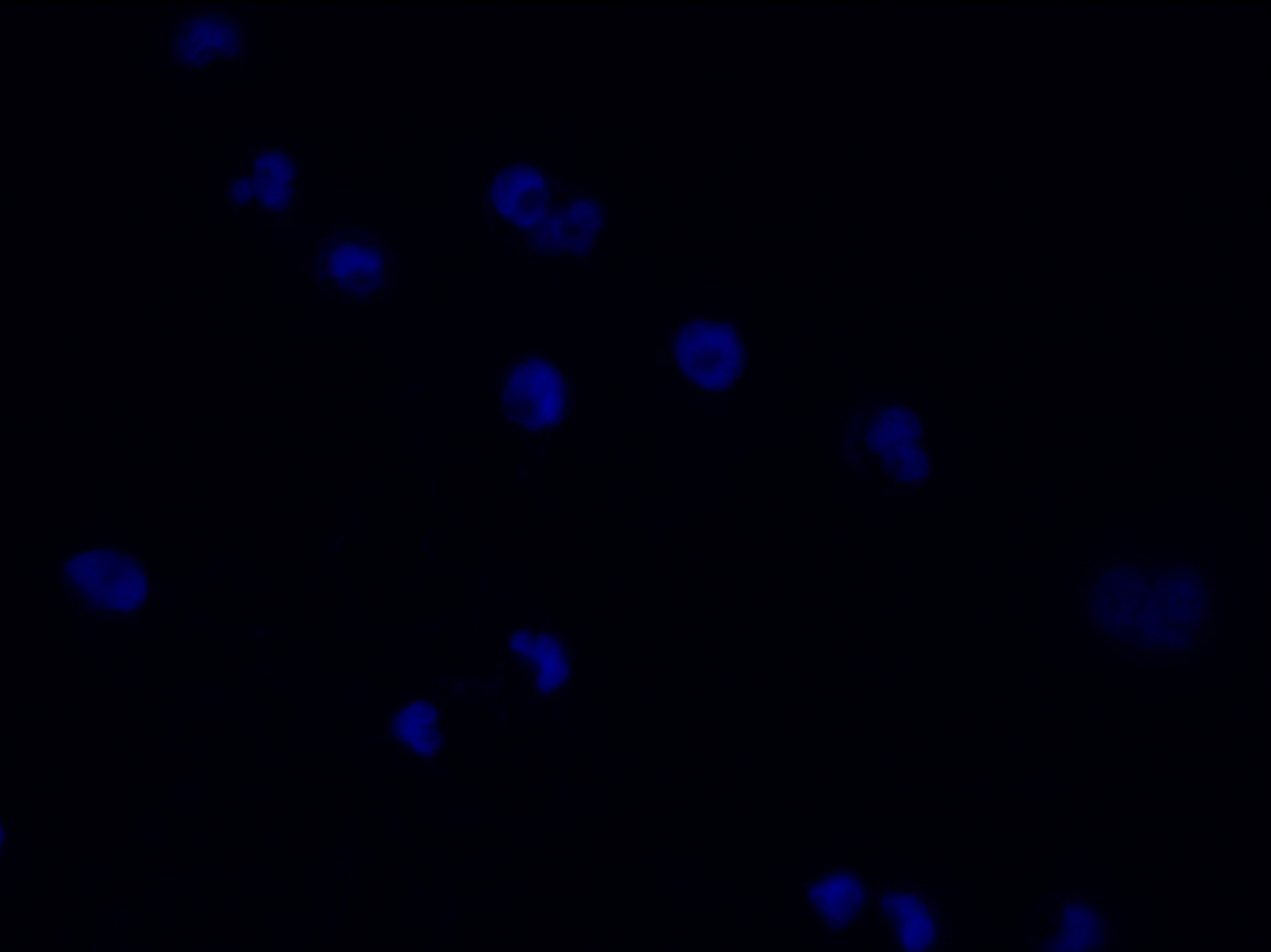

Supplement: S4 File — (ZIP) [file pone.0233739.s004.zip › S4_File/A549 Res-4h-2012-0011_c3.JPG]

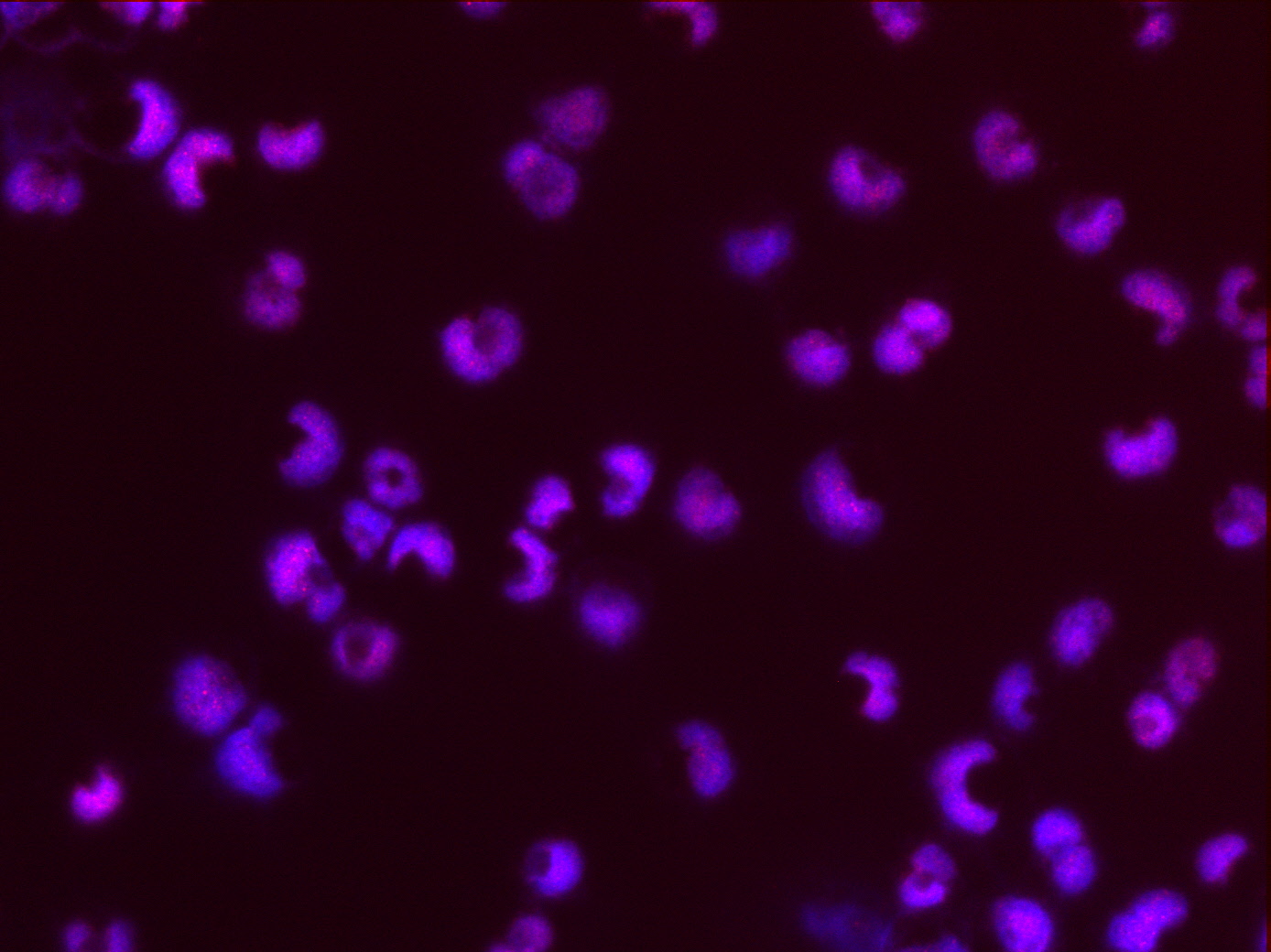

Supplement: S4 File — (ZIP) [file pone.0233739.s004.zip › S4_File/A549 Sens-12h-2012-0008_(c2+c3).JPG]

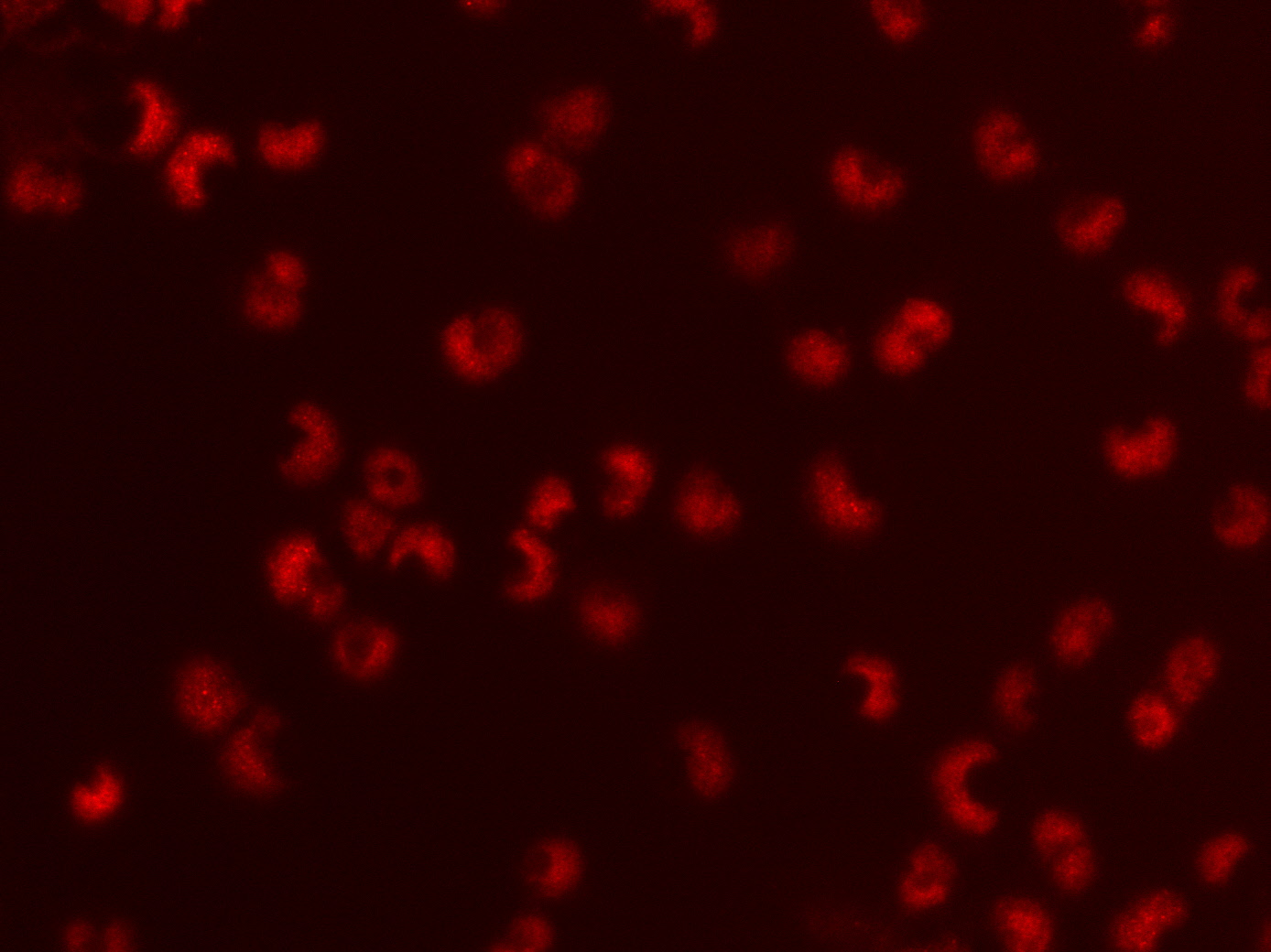

Supplement: S4 File — (ZIP) [file pone.0233739.s004.zip › S4_File/A549 Sens-12h-2012-0008_c2.JPG]

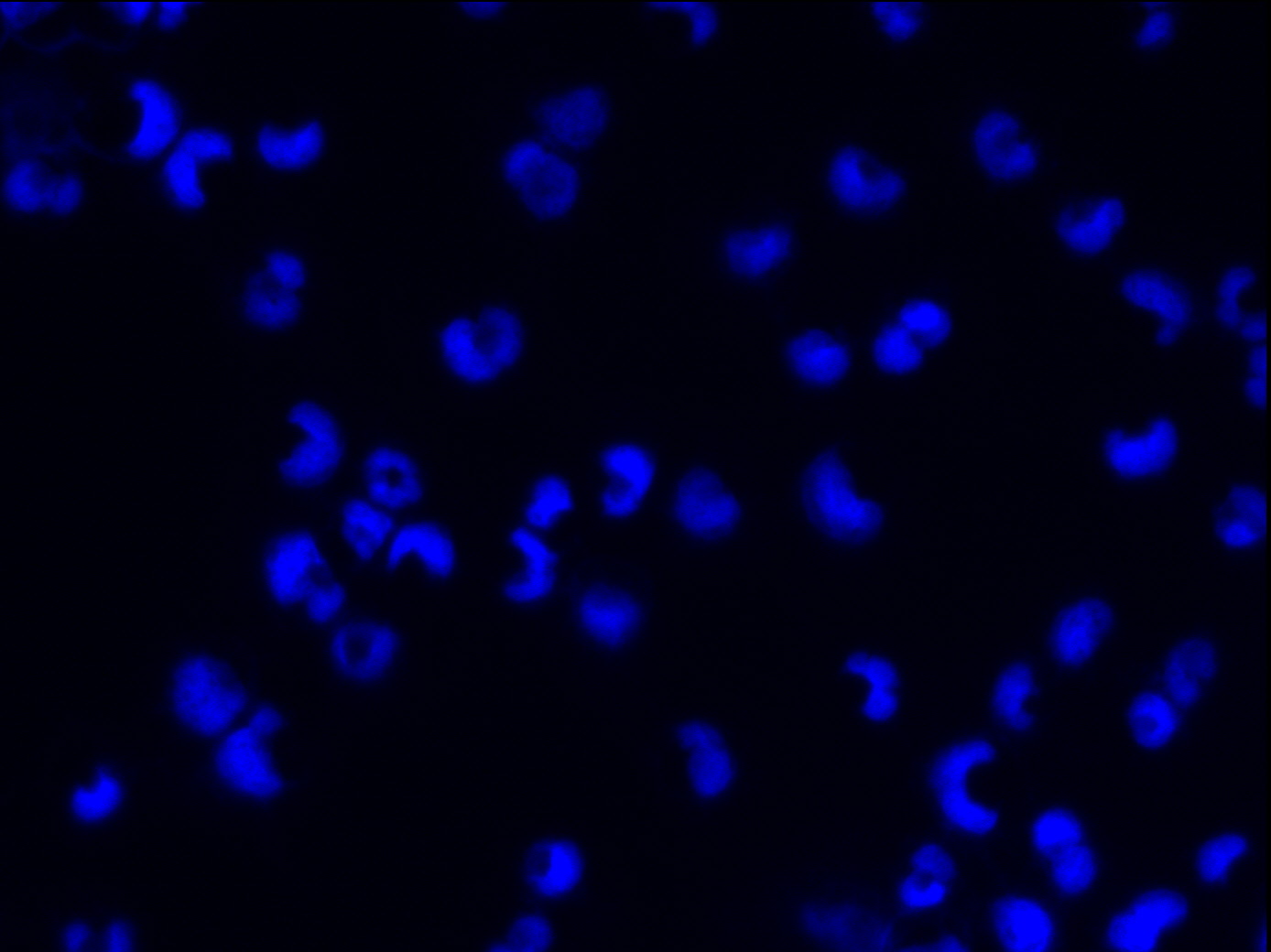

Supplement: S4 File — (ZIP) [file pone.0233739.s004.zip › S4_File/A549 Sens-12h-2012-0008_c3.JPG]

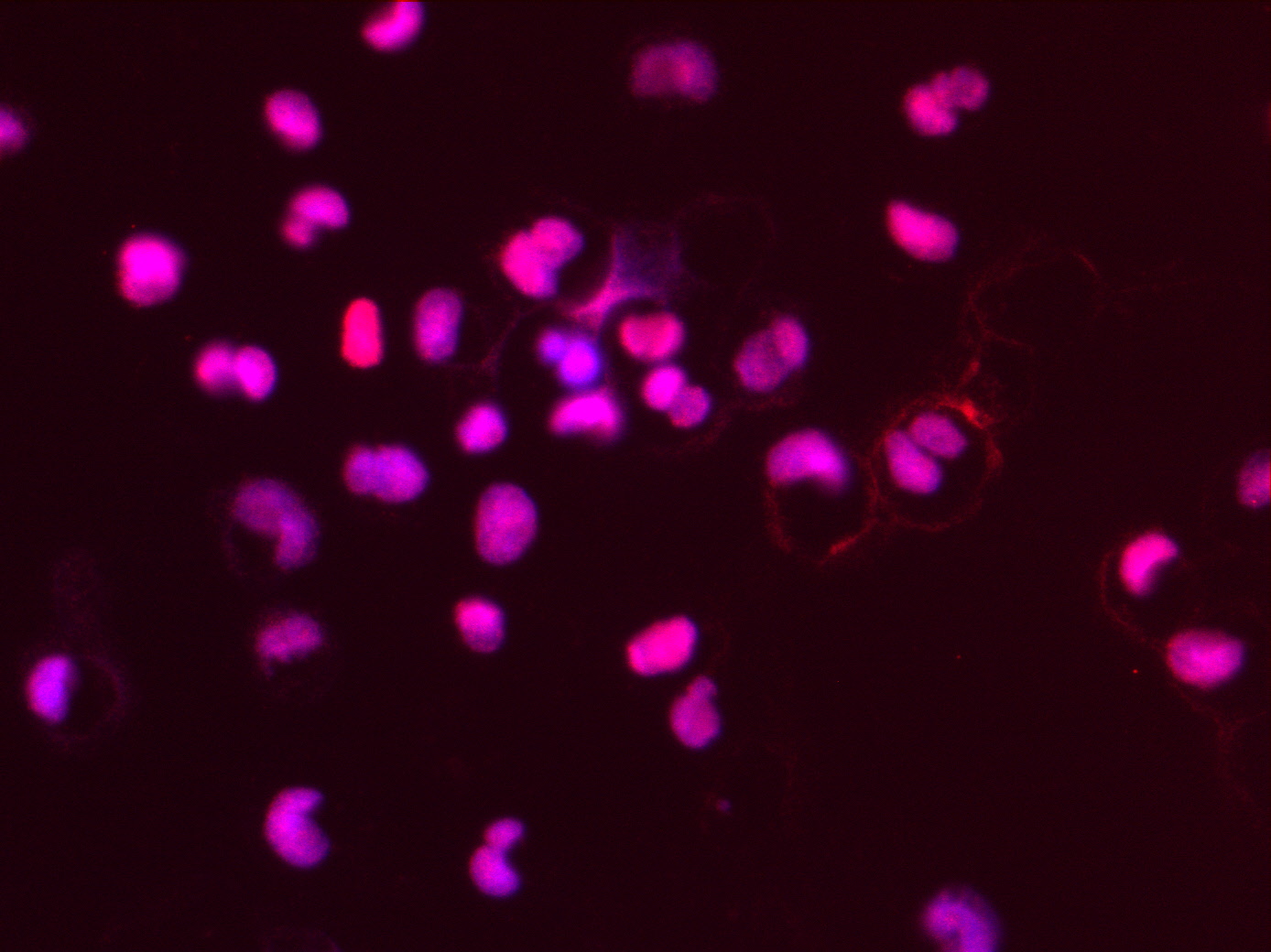

Supplement: S4 File — (ZIP) [file pone.0233739.s004.zip › S4_File/A549 Sens-24h-2012-0009_(c2+c3).JPG]

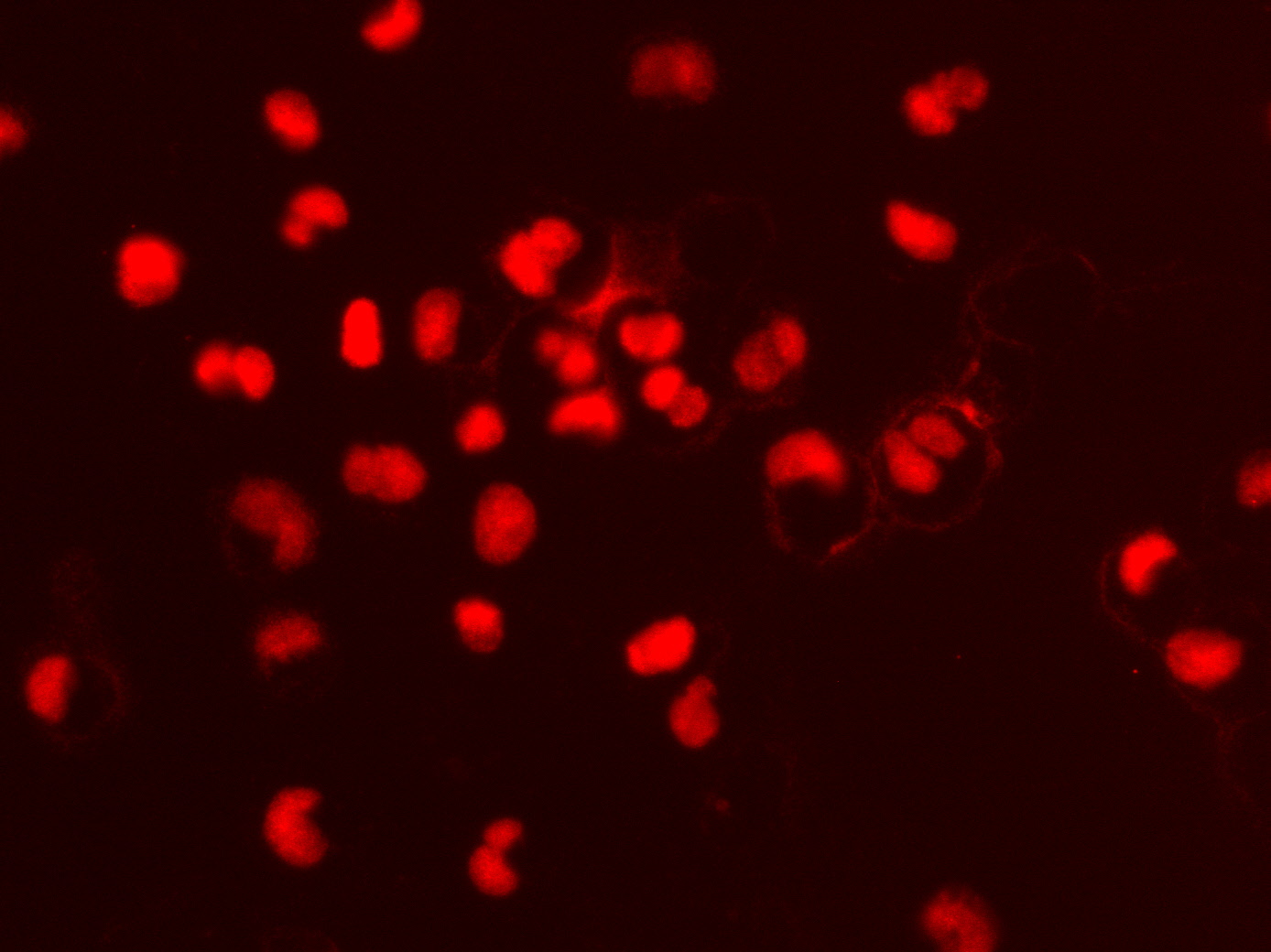

Supplement: S4 File — (ZIP) [file pone.0233739.s004.zip › S4_File/A549 Sens-24h-2012-0009_c2.JPG]

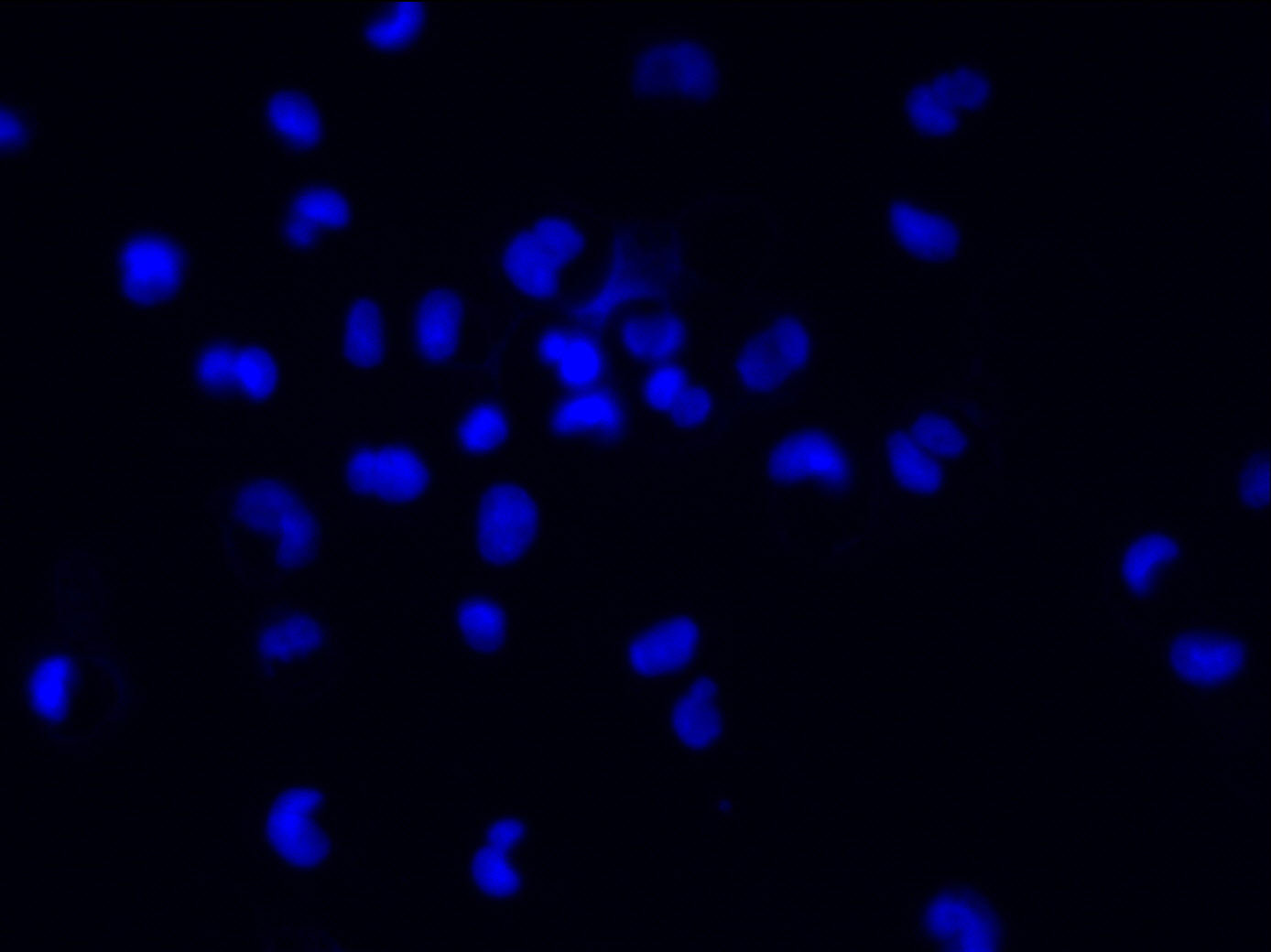

Supplement: S4 File — (ZIP) [file pone.0233739.s004.zip › S4_File/A549 Sens-24h-2012-0009_c3.JPG]

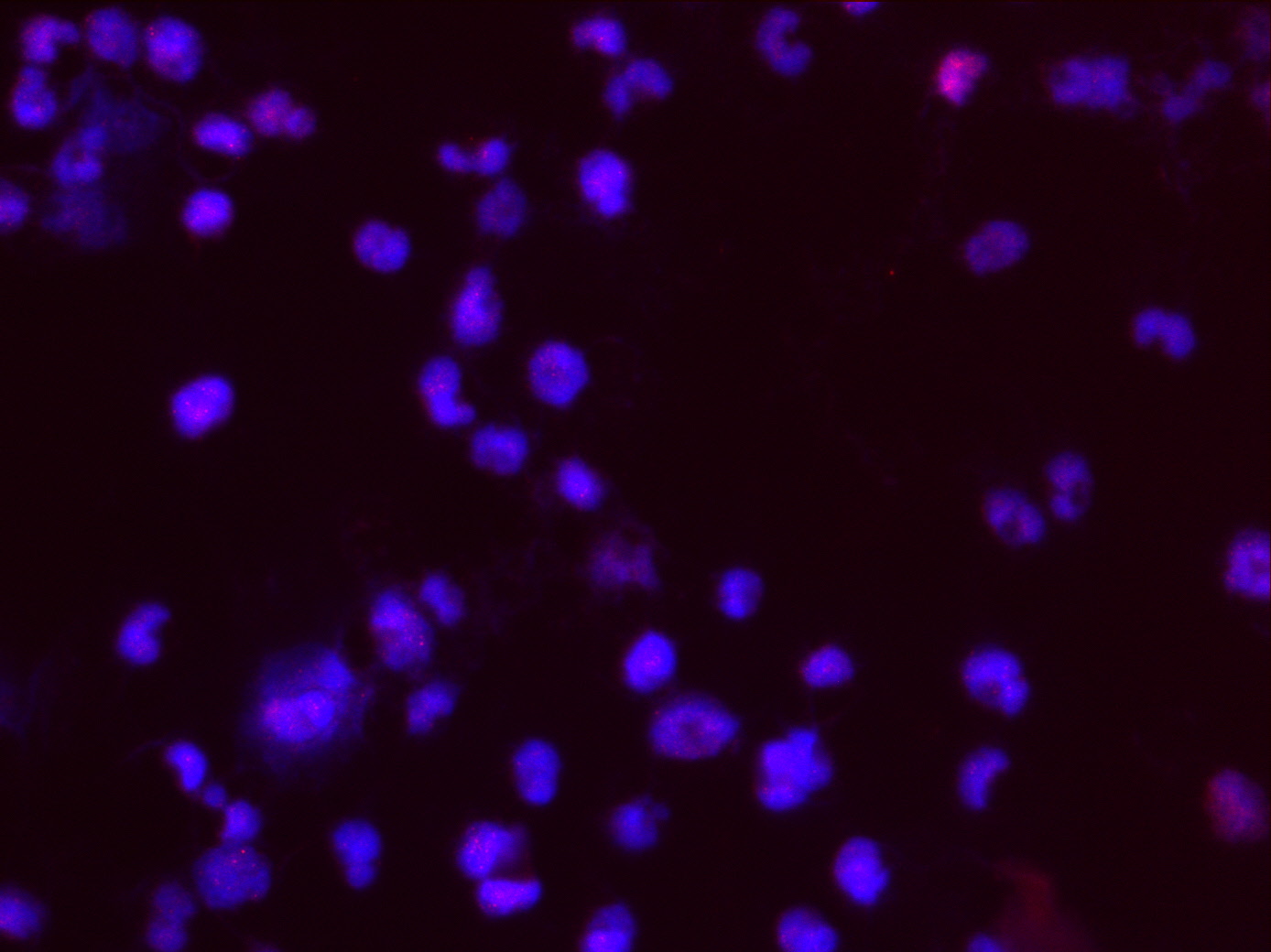

Supplement: S4 File — (ZIP) [file pone.0233739.s004.zip › S4_File/A549 Sens-4h-2012-0007_(c2+c3).JPG]

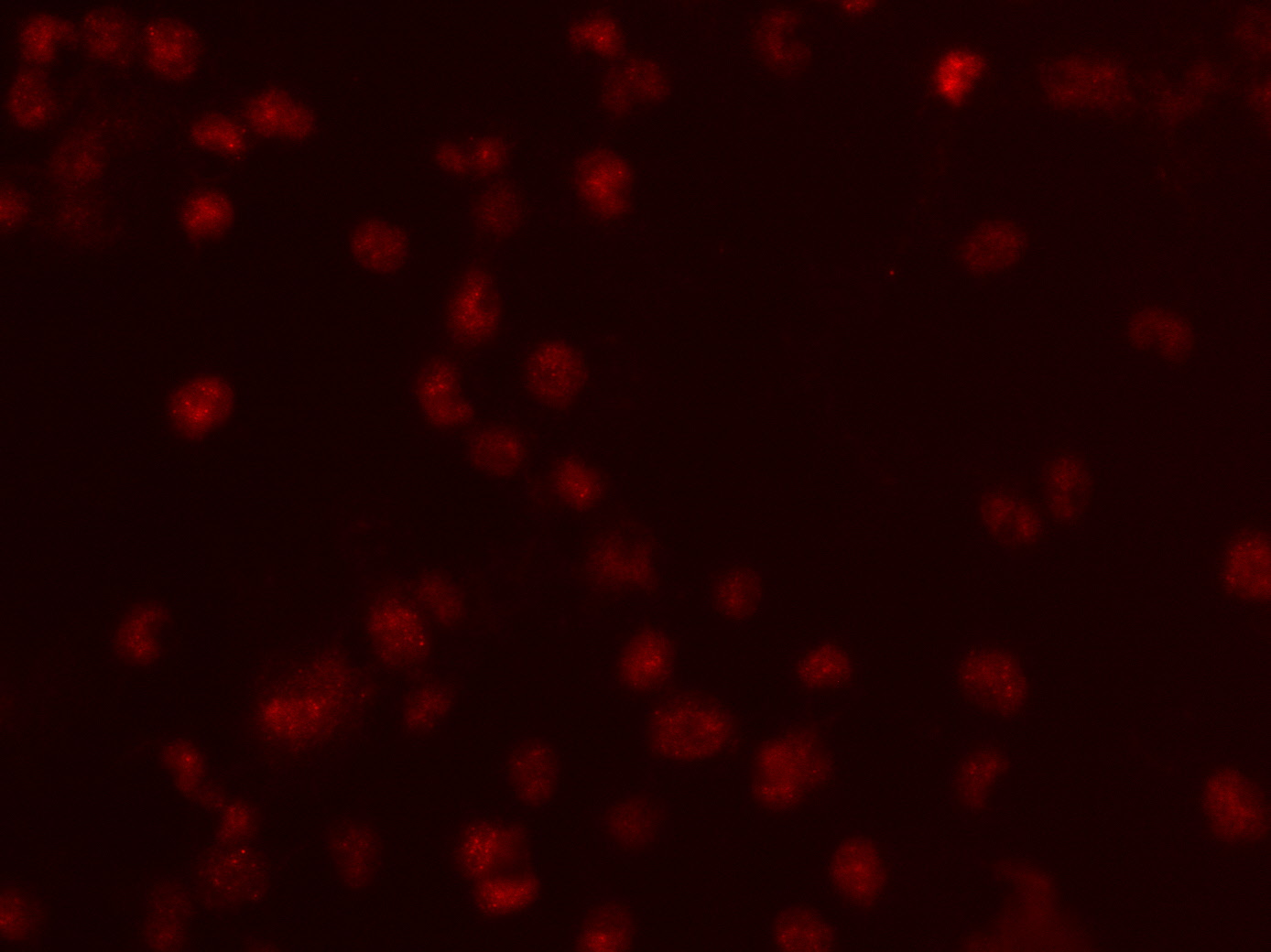

Supplement: S4 File — (ZIP) [file pone.0233739.s004.zip › S4_File/A549 Sens-4h-2012-0007_c2.JPG]

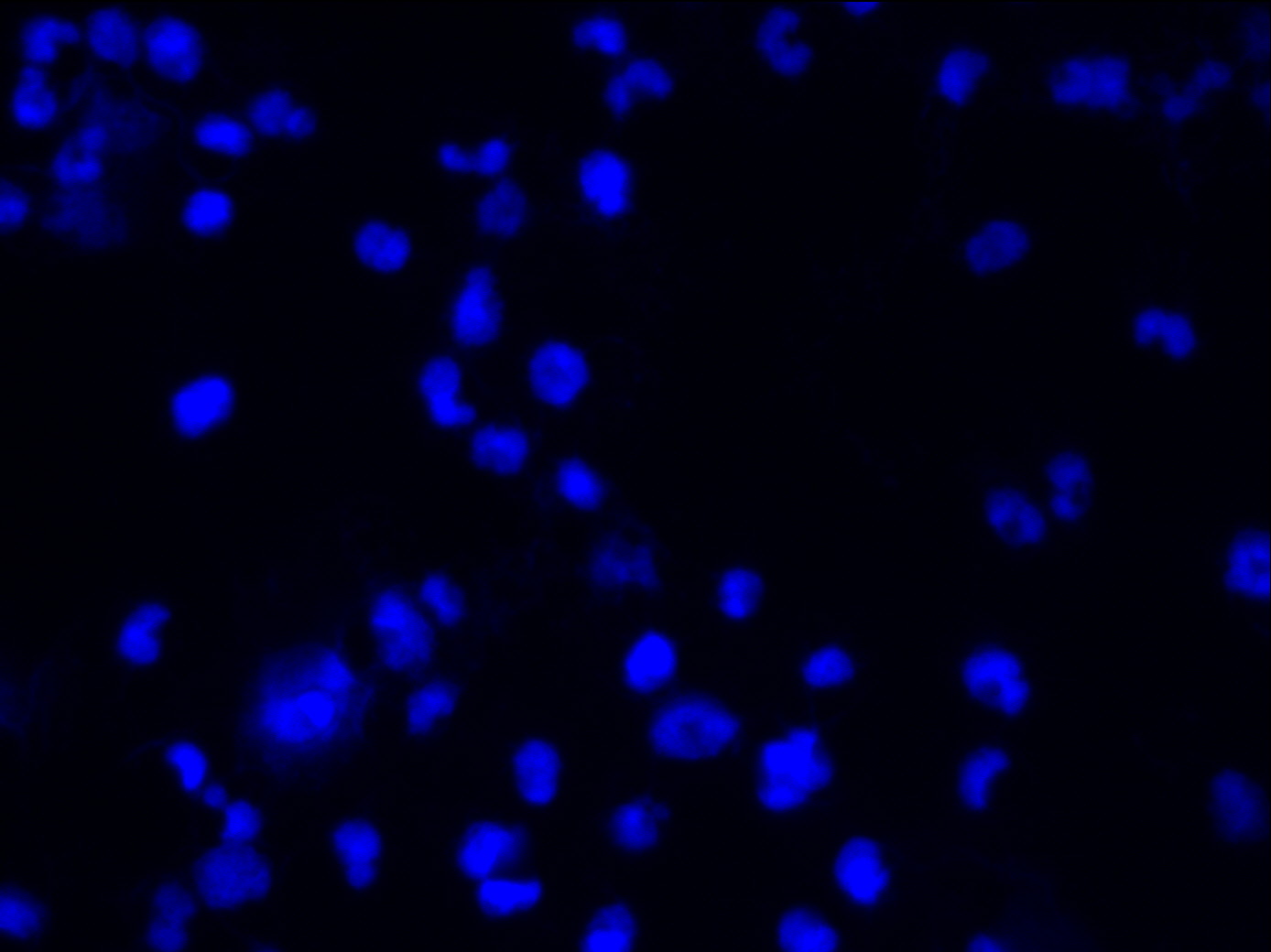

Supplement: S4 File — (ZIP) [file pone.0233739.s004.zip › S4_File/A549 Sens-4h-2012-0007_c3.JPG]

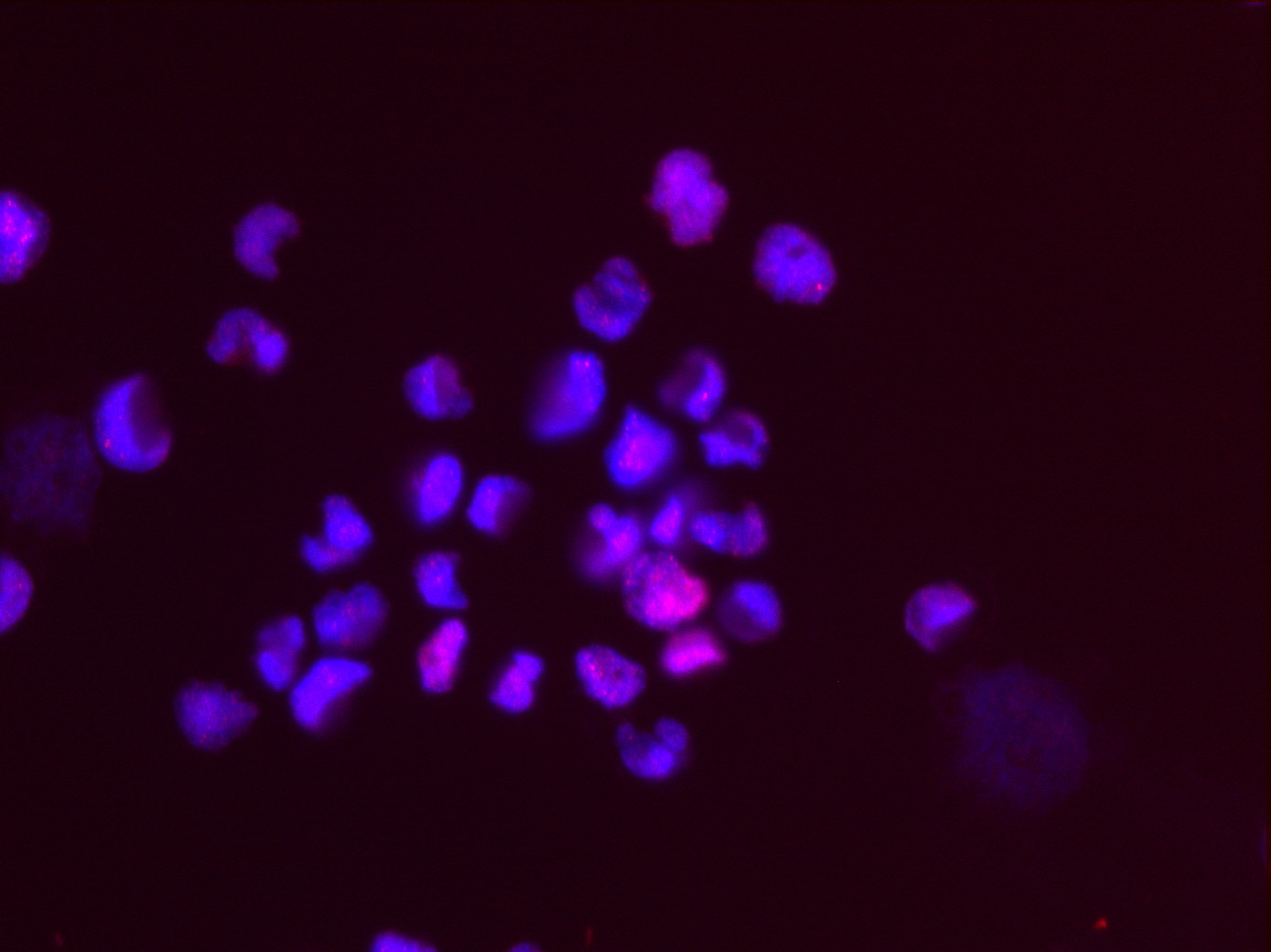

Supplement: S5 File — (ZIP) [file pone.0233739.s005.zip › S5_File/SKMES Res-12h-2012-0031_(c2+c3).JPG]

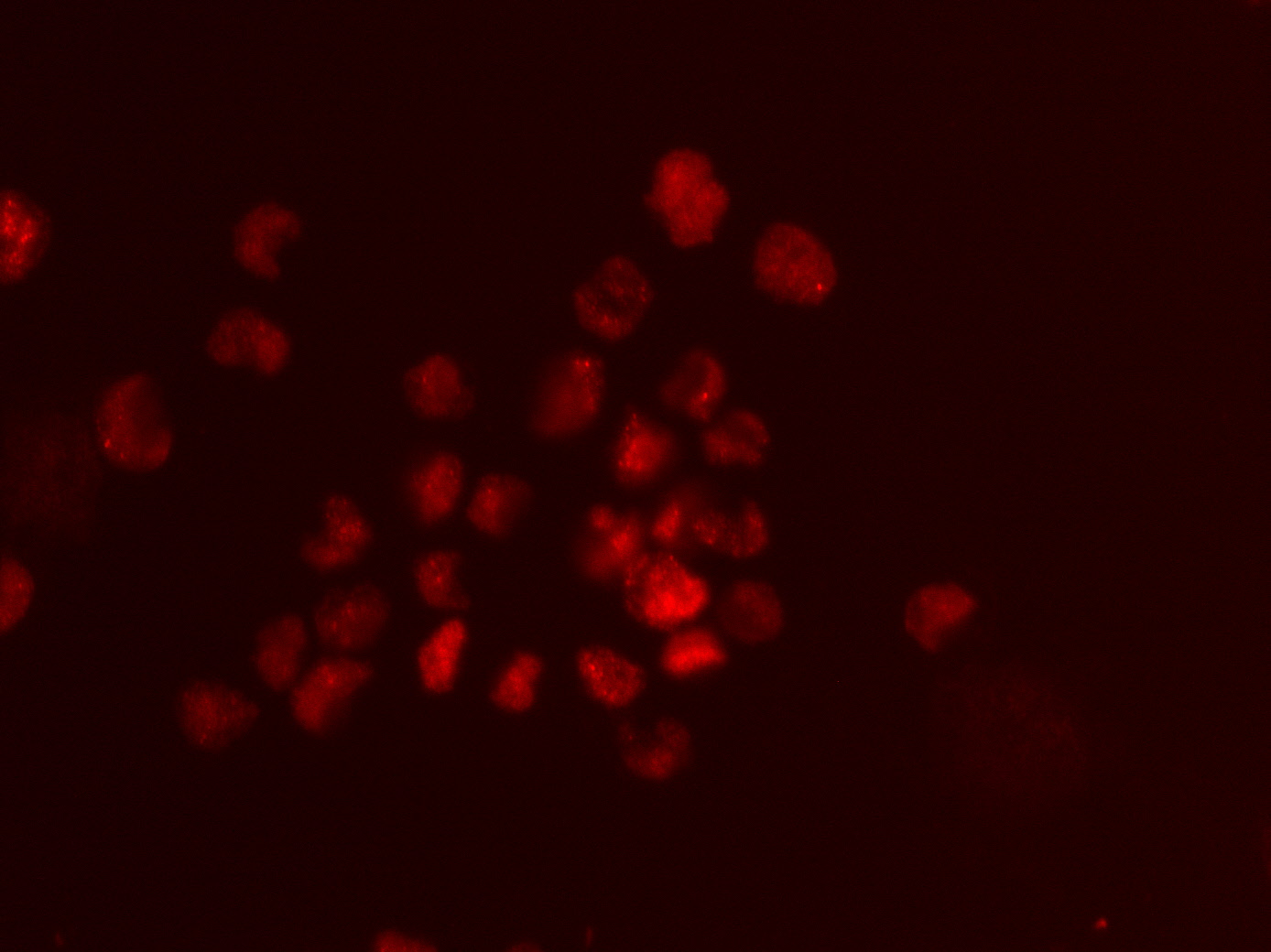

Supplement: S5 File — (ZIP) [file pone.0233739.s005.zip › S5_File/SKMES Res-12h-2012-0031_c2.JPG]

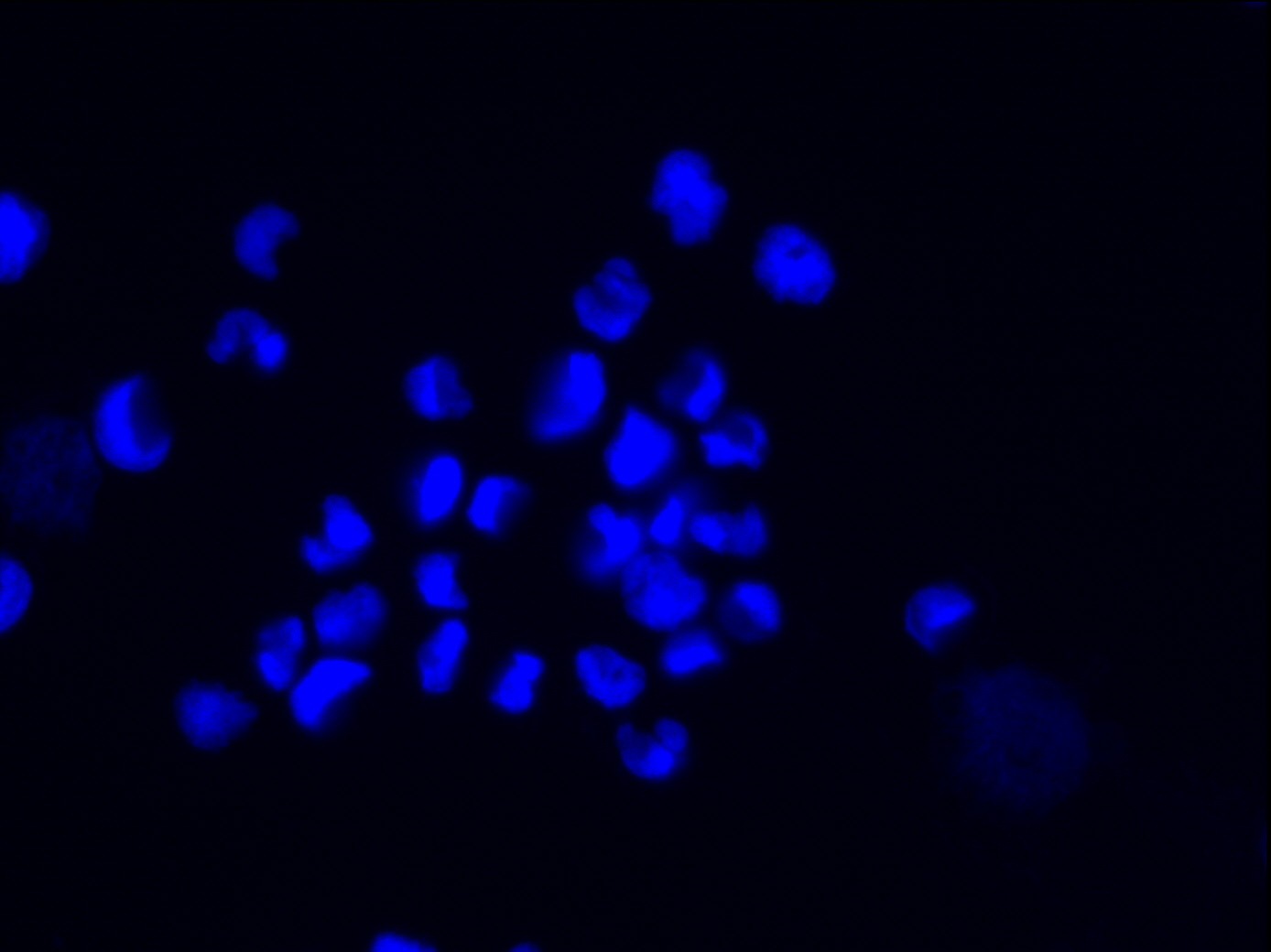

Supplement: S5 File — (ZIP) [file pone.0233739.s005.zip › S5_File/SKMES Res-12h-2012-0031_c3.JPG]

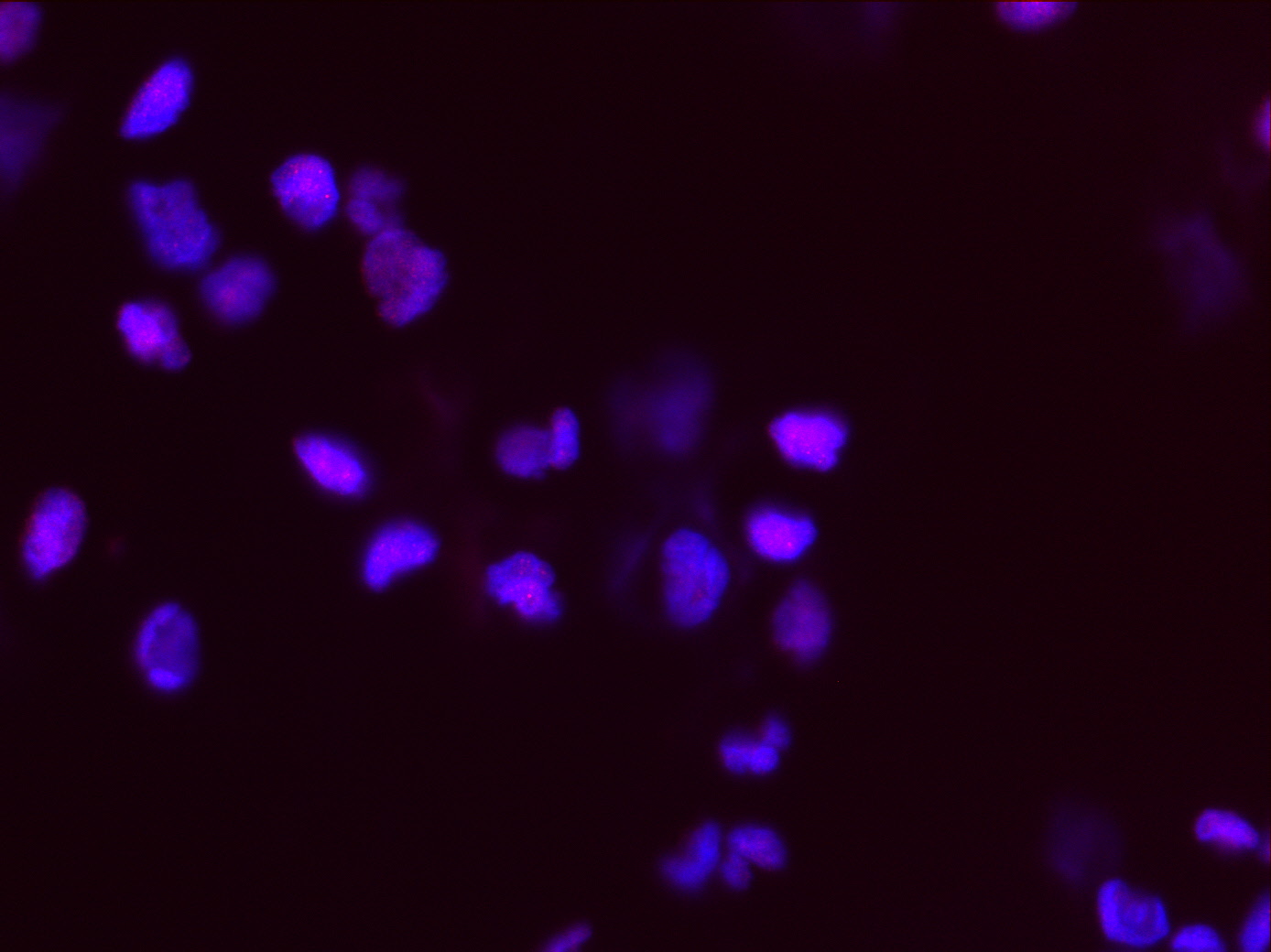

Supplement: S5 File — (ZIP) [file pone.0233739.s005.zip › S5_File/SKMES Res-24h2012-0032_(c2+c3).JPG]

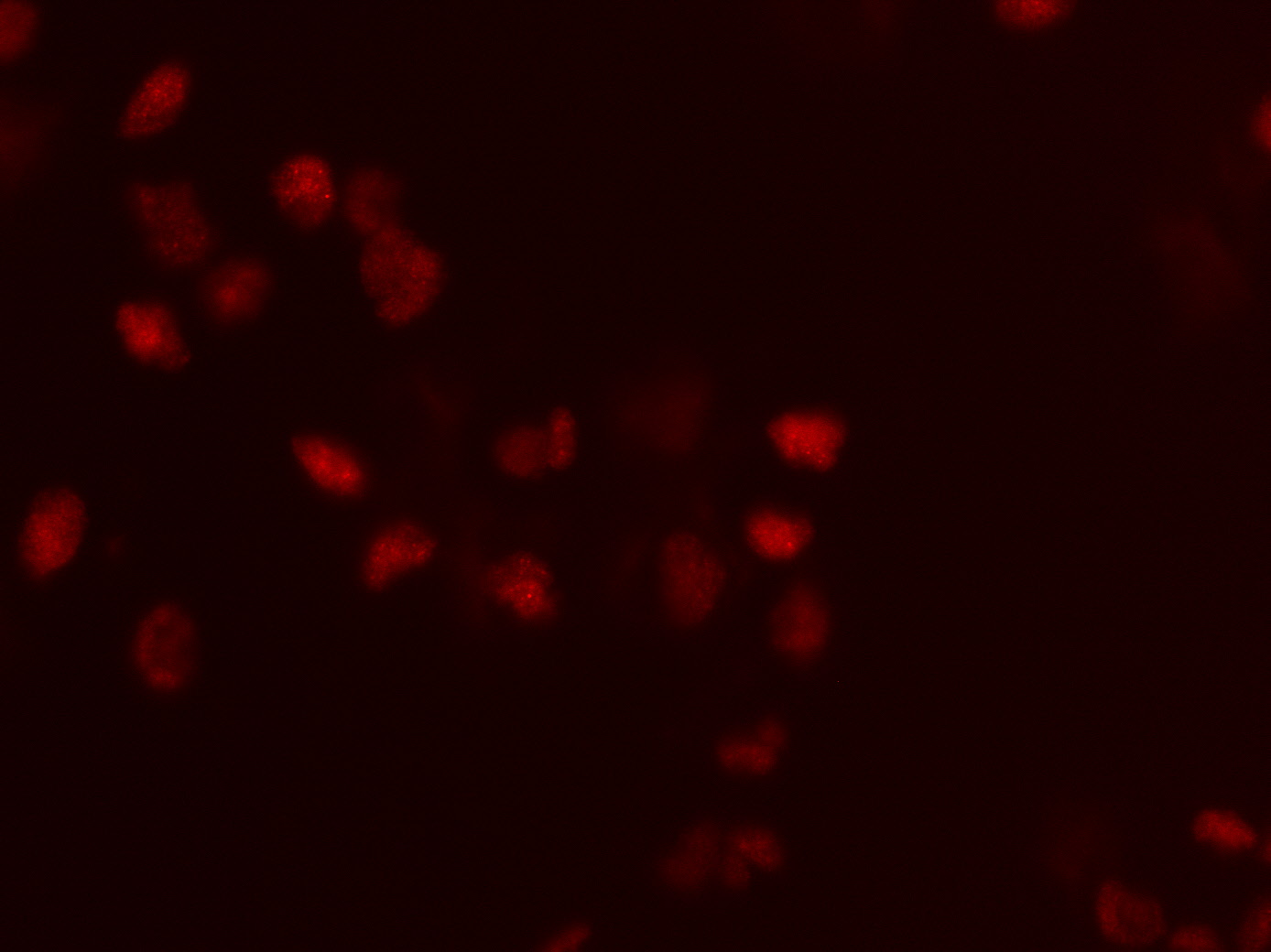

Supplement: S5 File — (ZIP) [file pone.0233739.s005.zip › S5_File/SKMES Res-24h-2012-0032_c2.JPG]

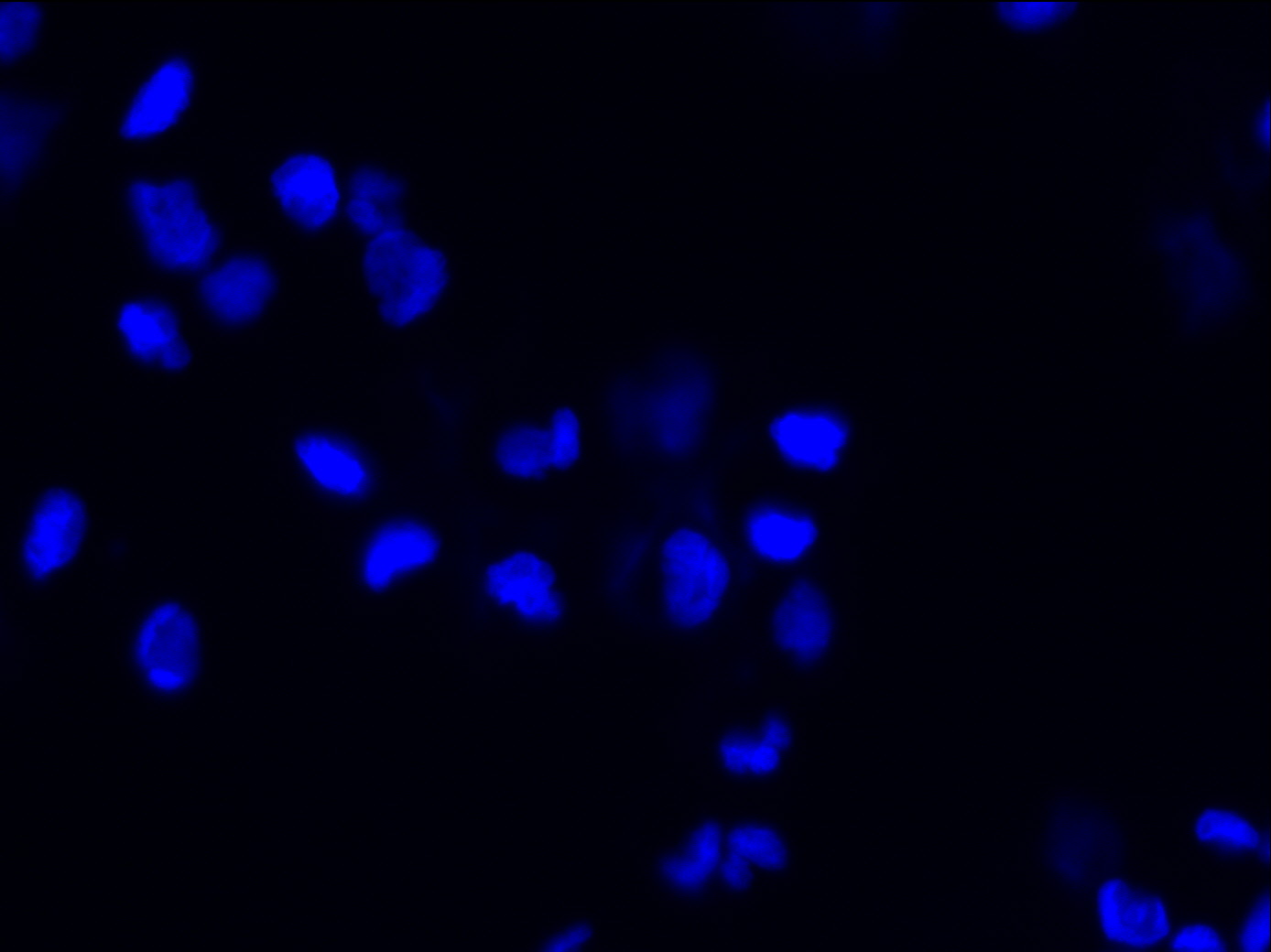

Supplement: S5 File — (ZIP) [file pone.0233739.s005.zip › S5_File/SKMES Res-24h-2012-0032_c3.JPG]

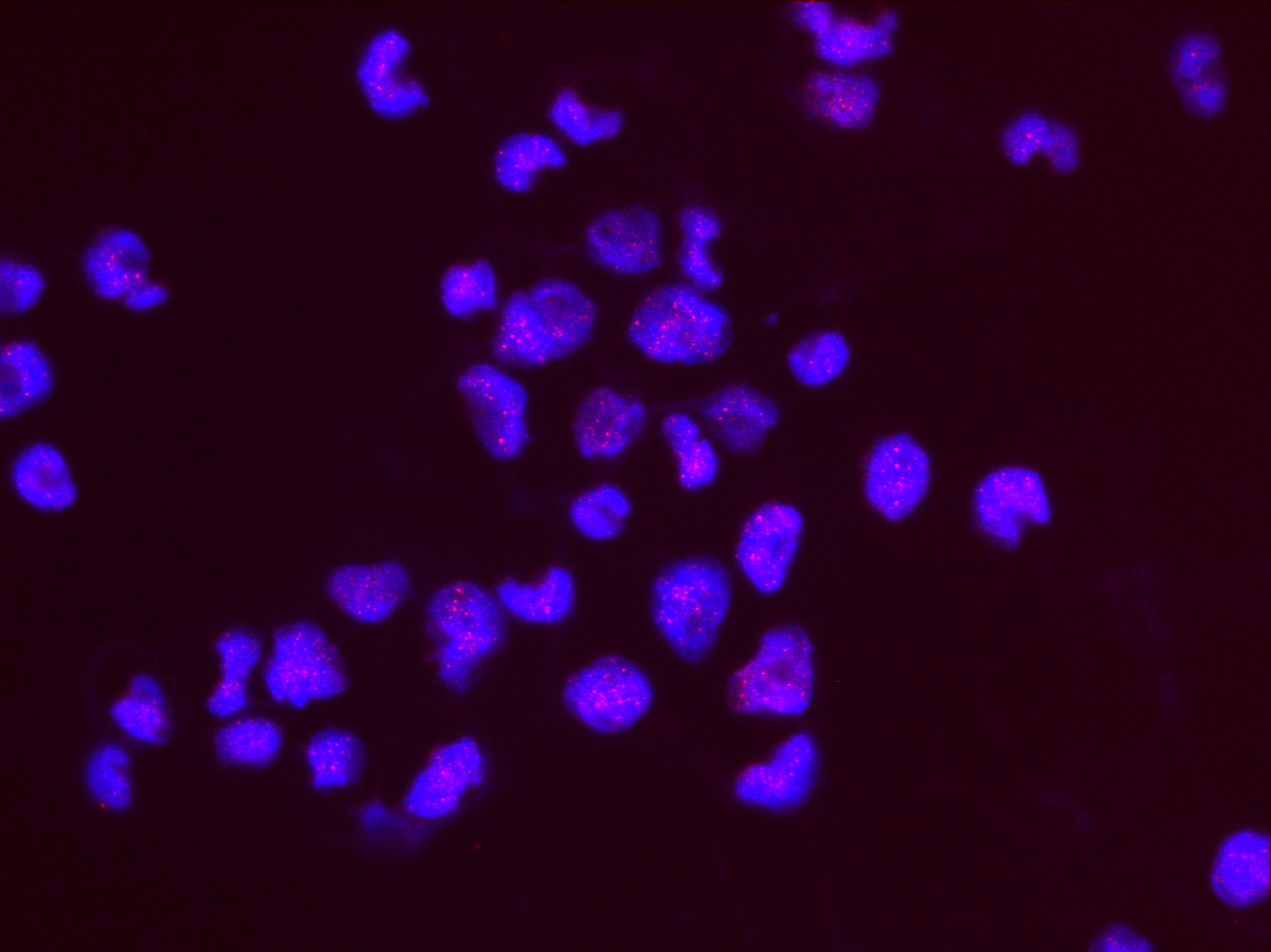

Supplement: S5 File — (ZIP) [file pone.0233739.s005.zip › S5_File/SKMES Res-4h-2012-0030_(c2+c3).JPG]

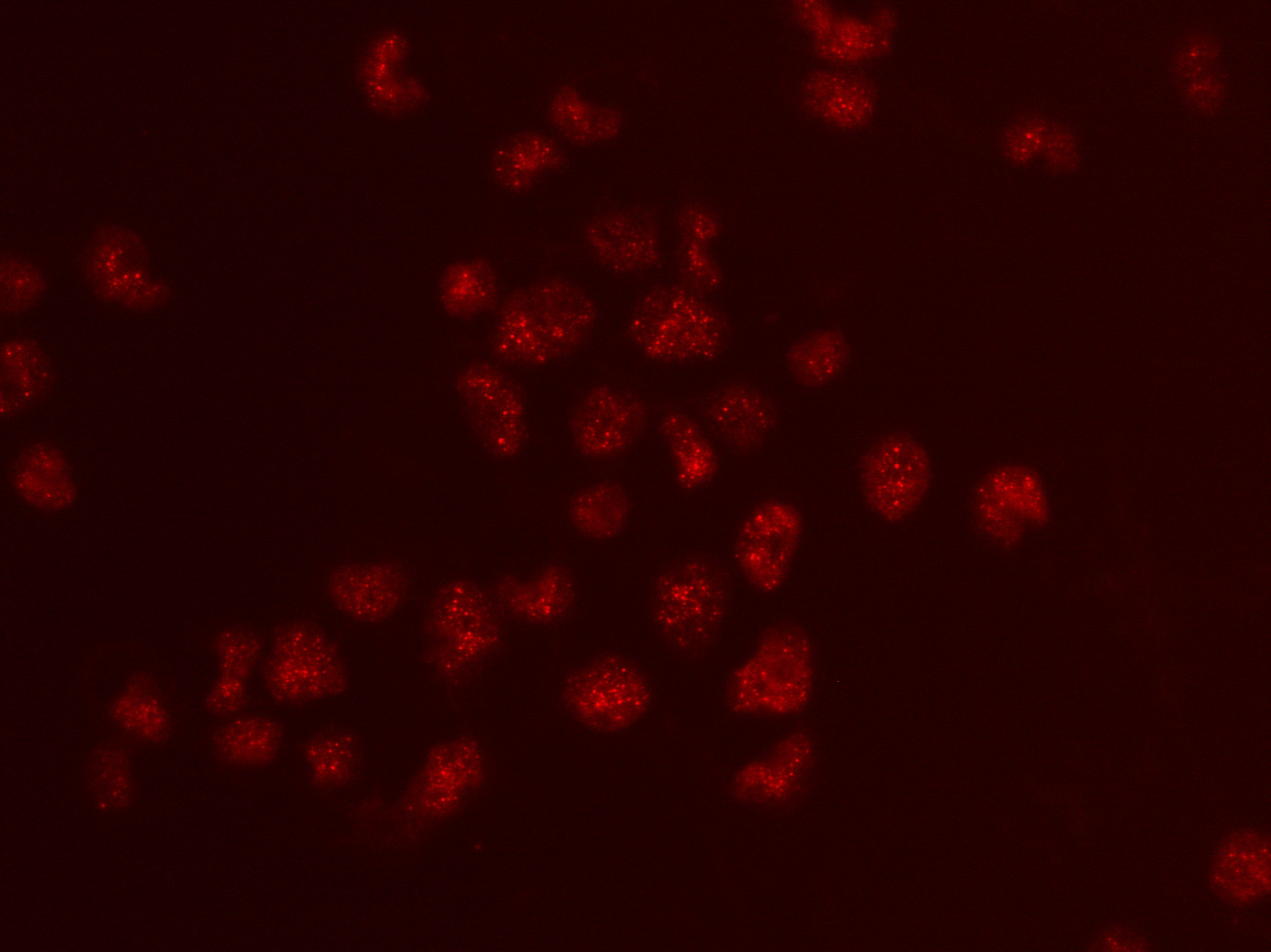

Supplement: S5 File — (ZIP) [file pone.0233739.s005.zip › S5_File/SKMES Res-4h-2012-0030_c2.JPG]

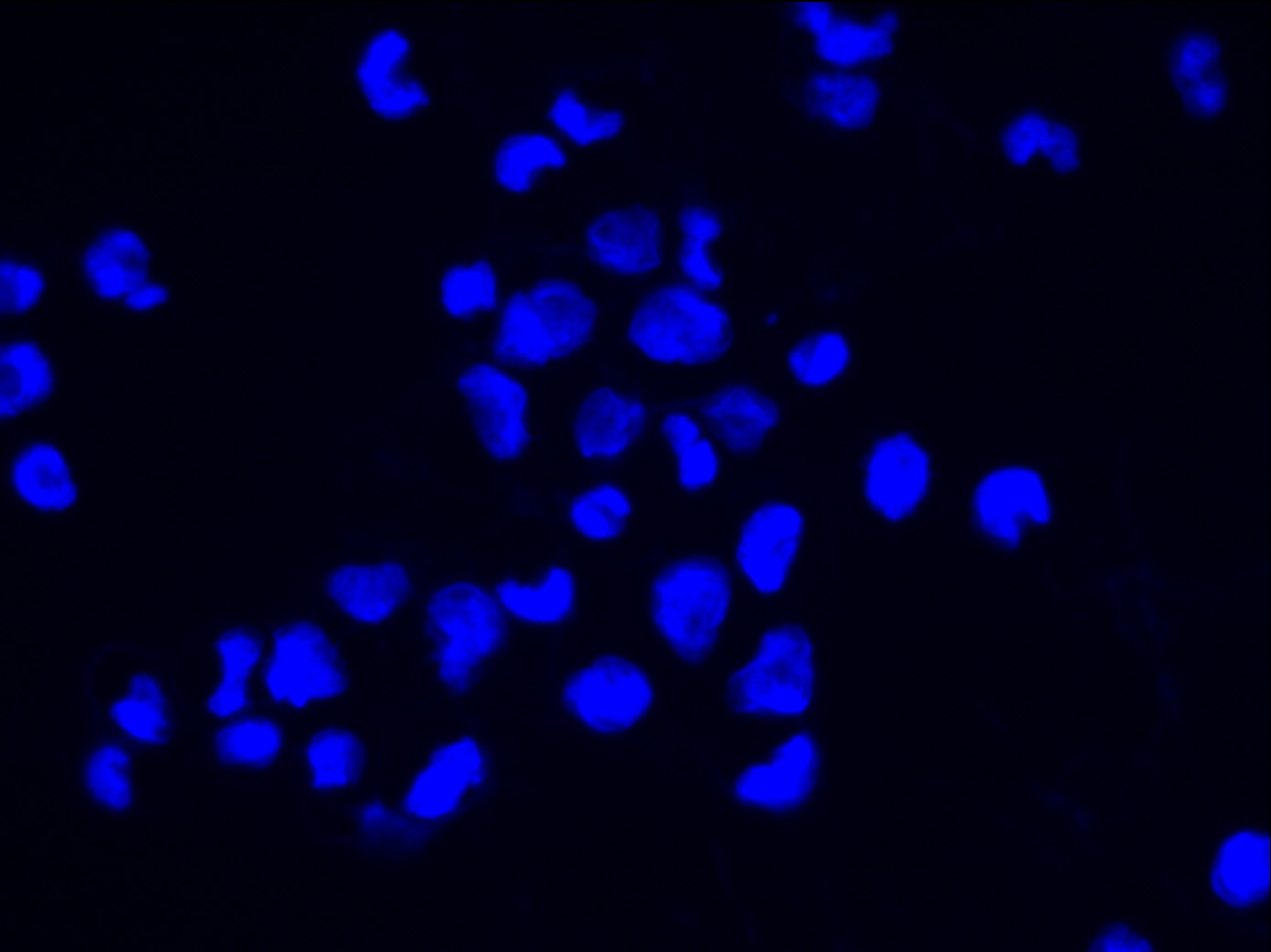

Supplement: S5 File — (ZIP) [file pone.0233739.s005.zip › S5_File/SKMES Res-4h-2012-0030_c3.JPG]

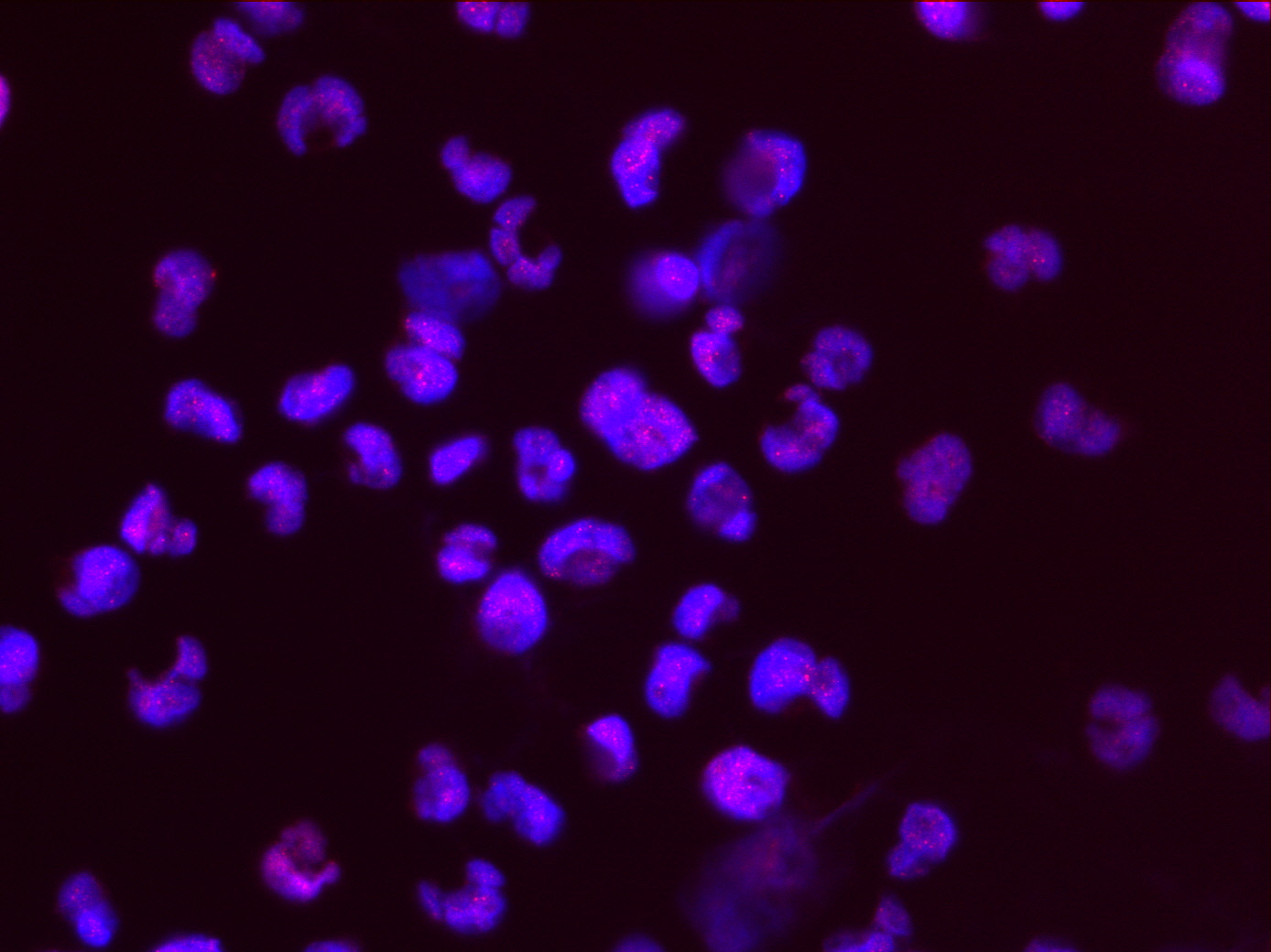

Supplement: S5 File — (ZIP) [file pone.0233739.s005.zip › S5_File/SKMES Sens-12h-2012-0028_(c2+c3).JPG]

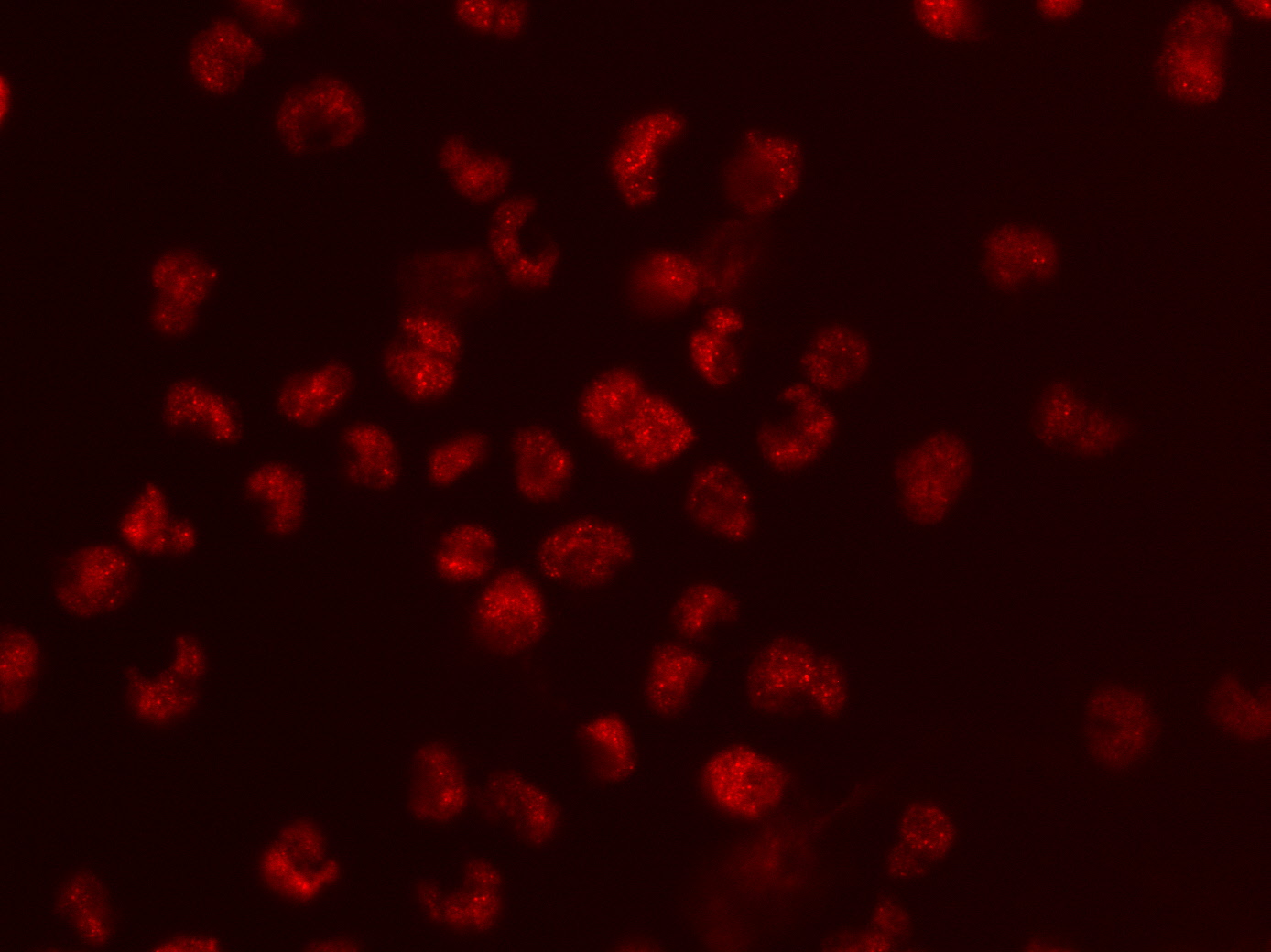

Supplement: S5 File — (ZIP) [file pone.0233739.s005.zip › S5_File/SKMES Sens-12h-2012-0028_c2.JPG]

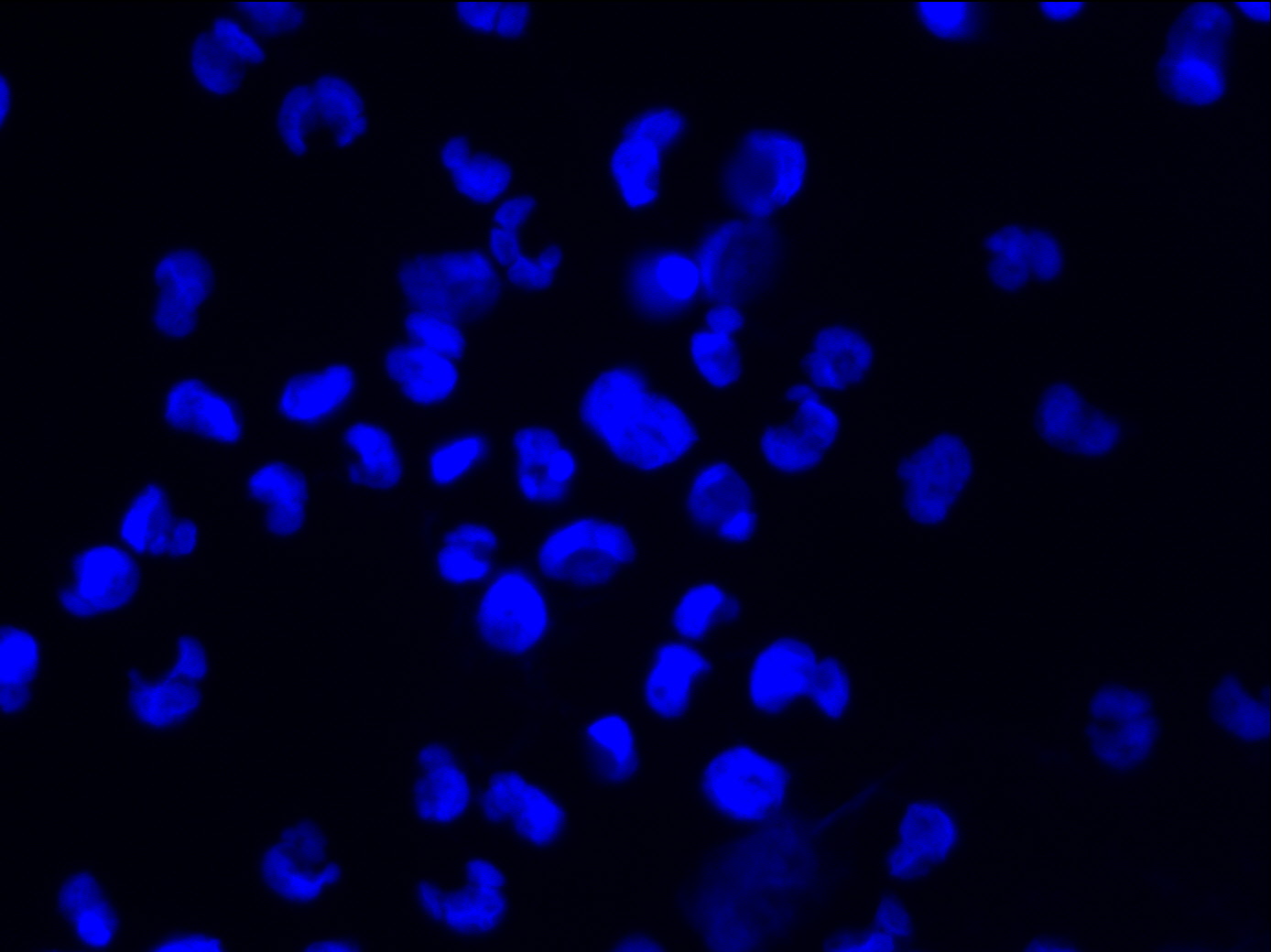

Supplement: S5 File — (ZIP) [file pone.0233739.s005.zip › S5_File/SKMES Sens-12h-2012-0028_c3.JPG]

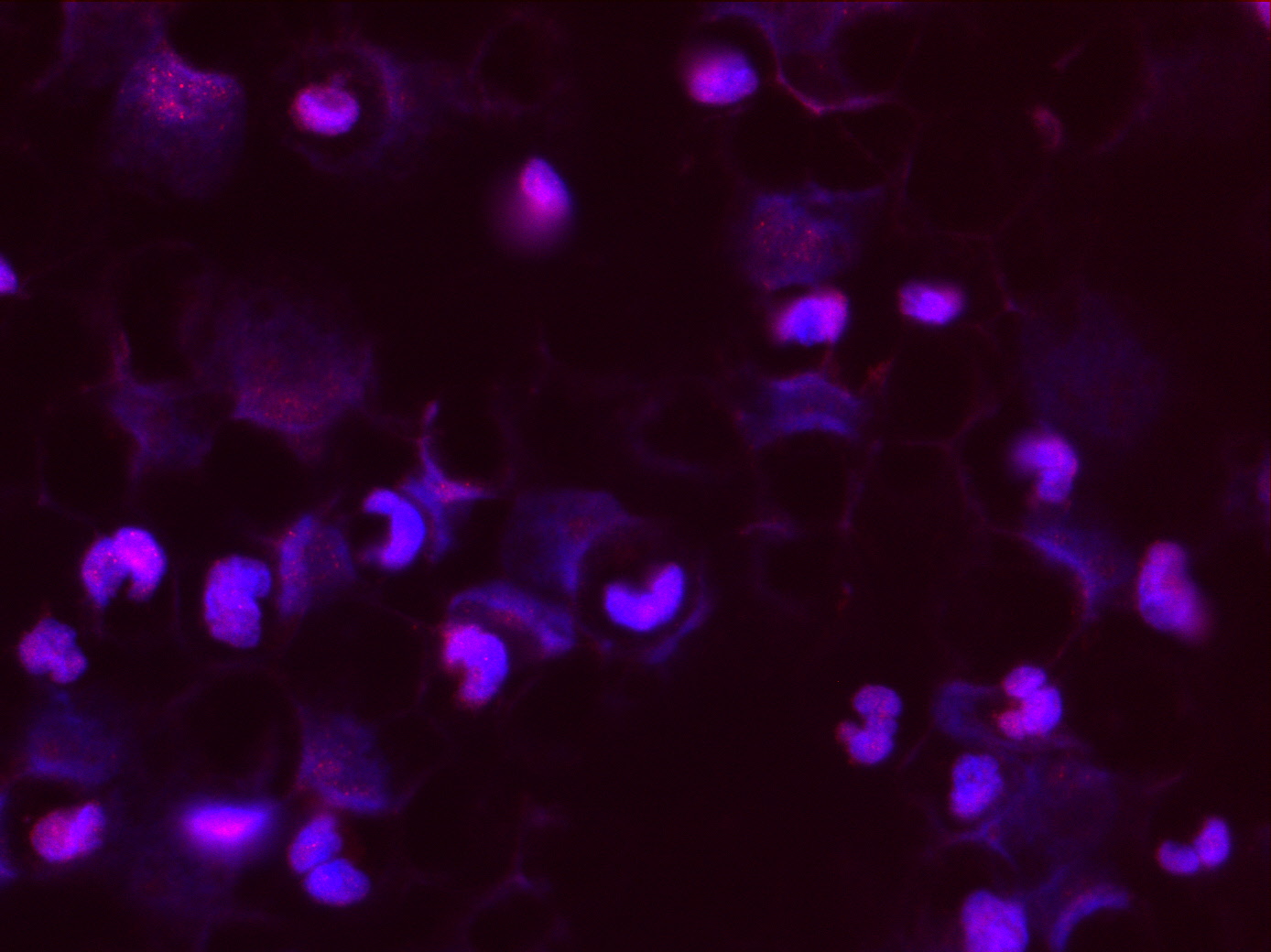

Supplement: S5 File — (ZIP) [file pone.0233739.s005.zip › S5_File/SKMES Sens-24h-2012-0029_(c2+c3).JPG]

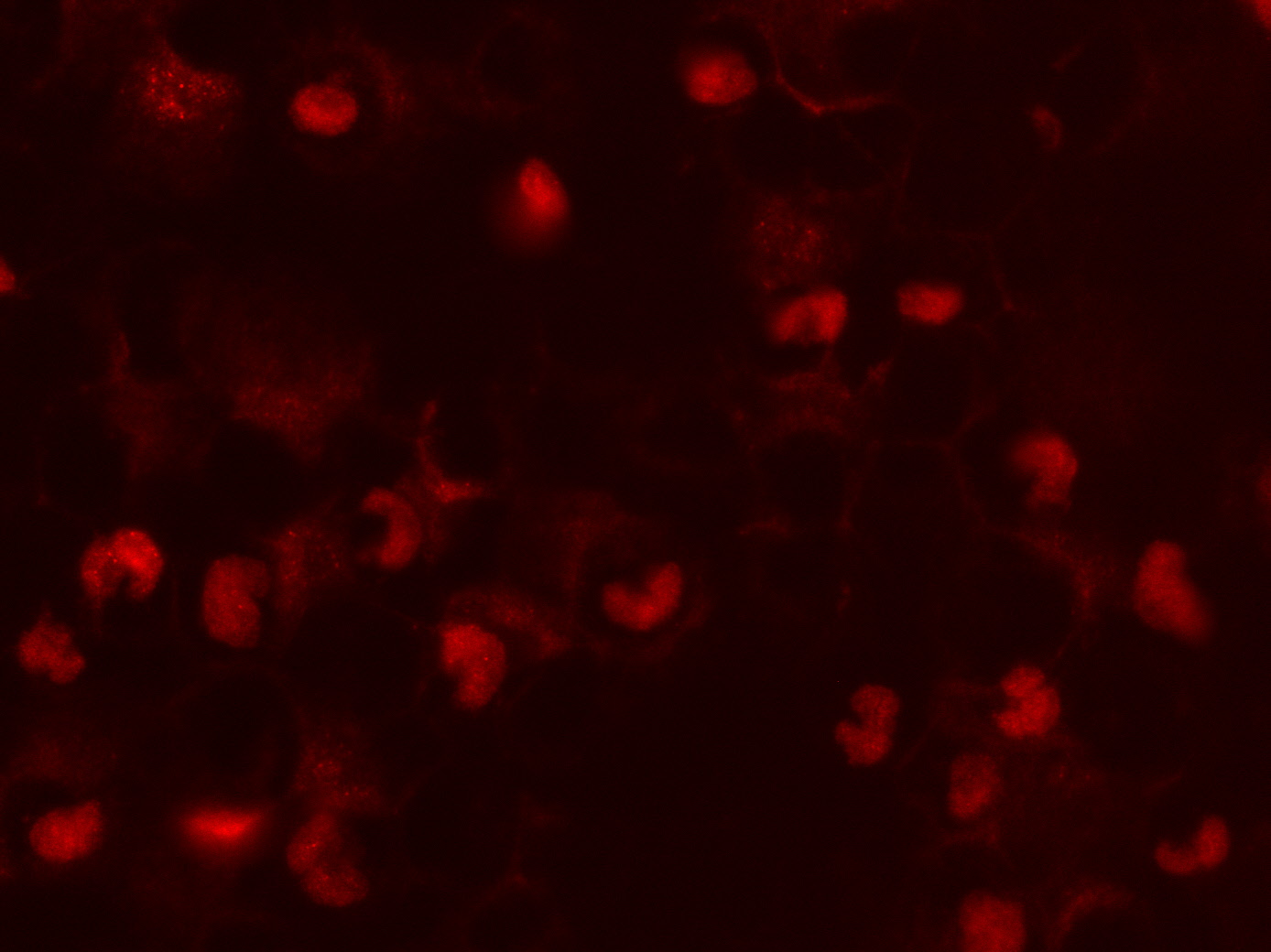

Supplement: S5 File — (ZIP) [file pone.0233739.s005.zip › S5_File/SKMES Sens-24h-2012-0029_c2.JPG]

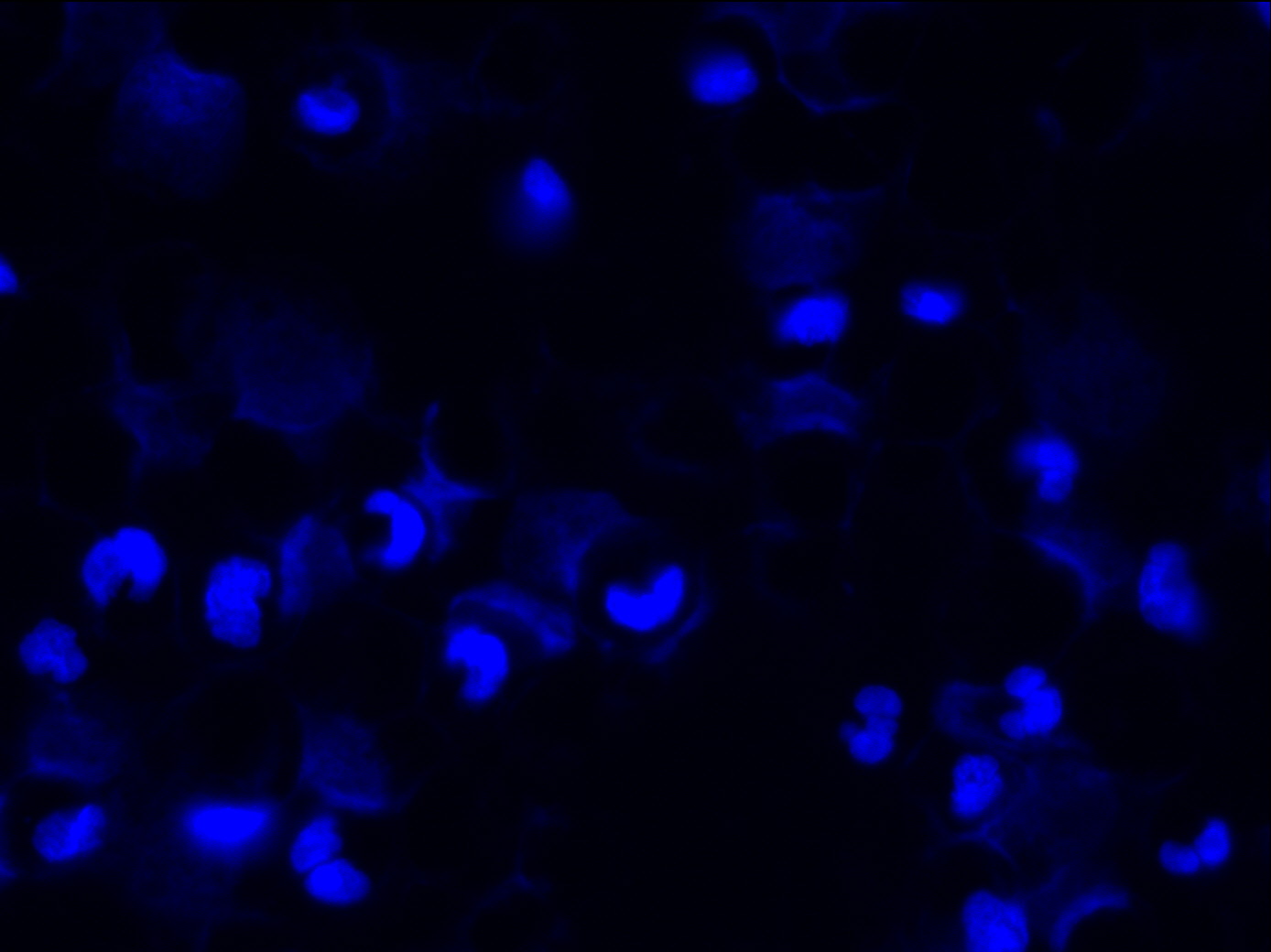

Supplement: S5 File — (ZIP) [file pone.0233739.s005.zip › S5_File/SKMES Sens-24h-2012-0029_c3.JPG]

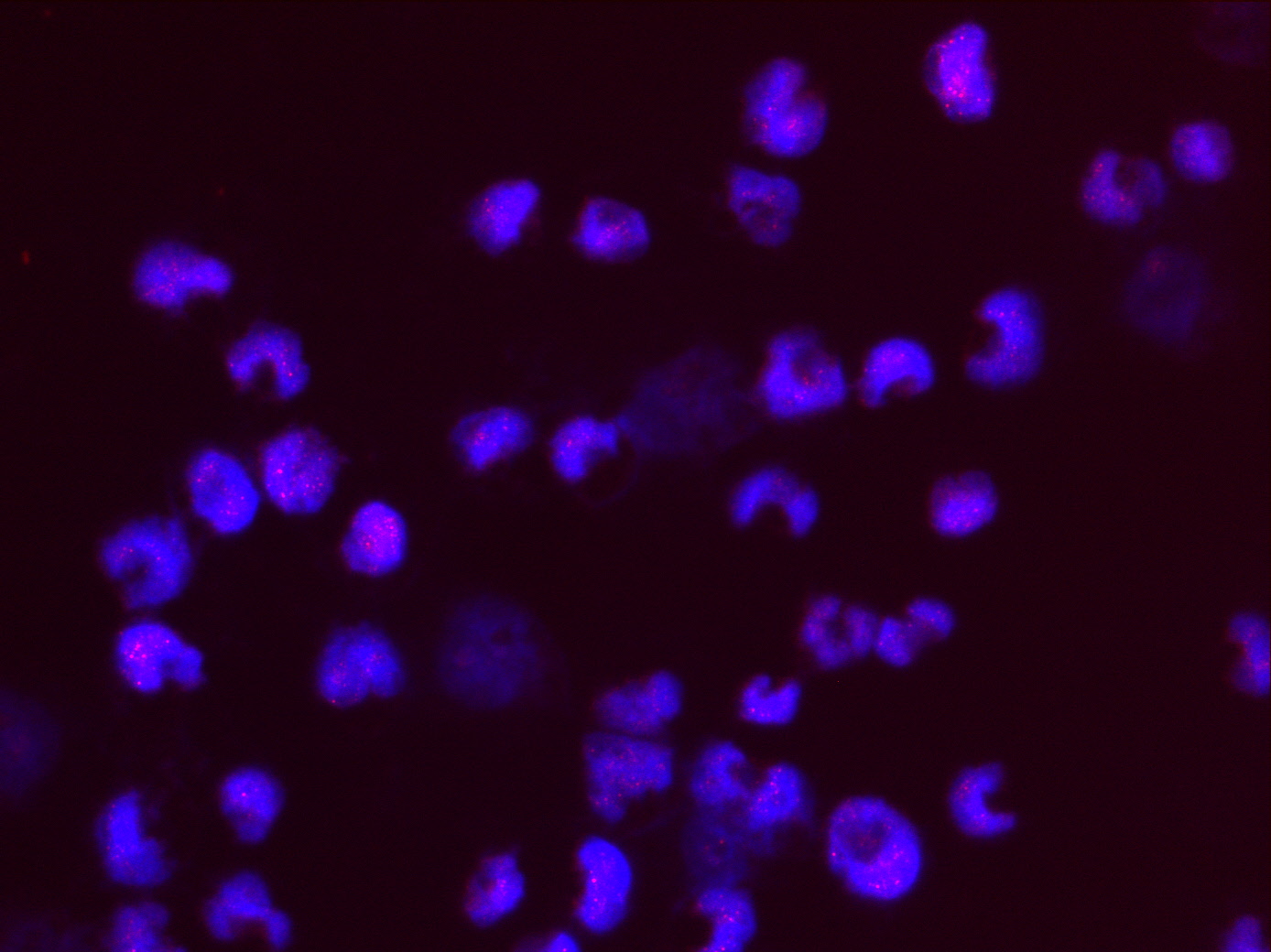

Supplement: S5 File — (ZIP) [file pone.0233739.s005.zip › S5_File/SKMES Sens-4h-2012-0027_(c2+c3).JPG]

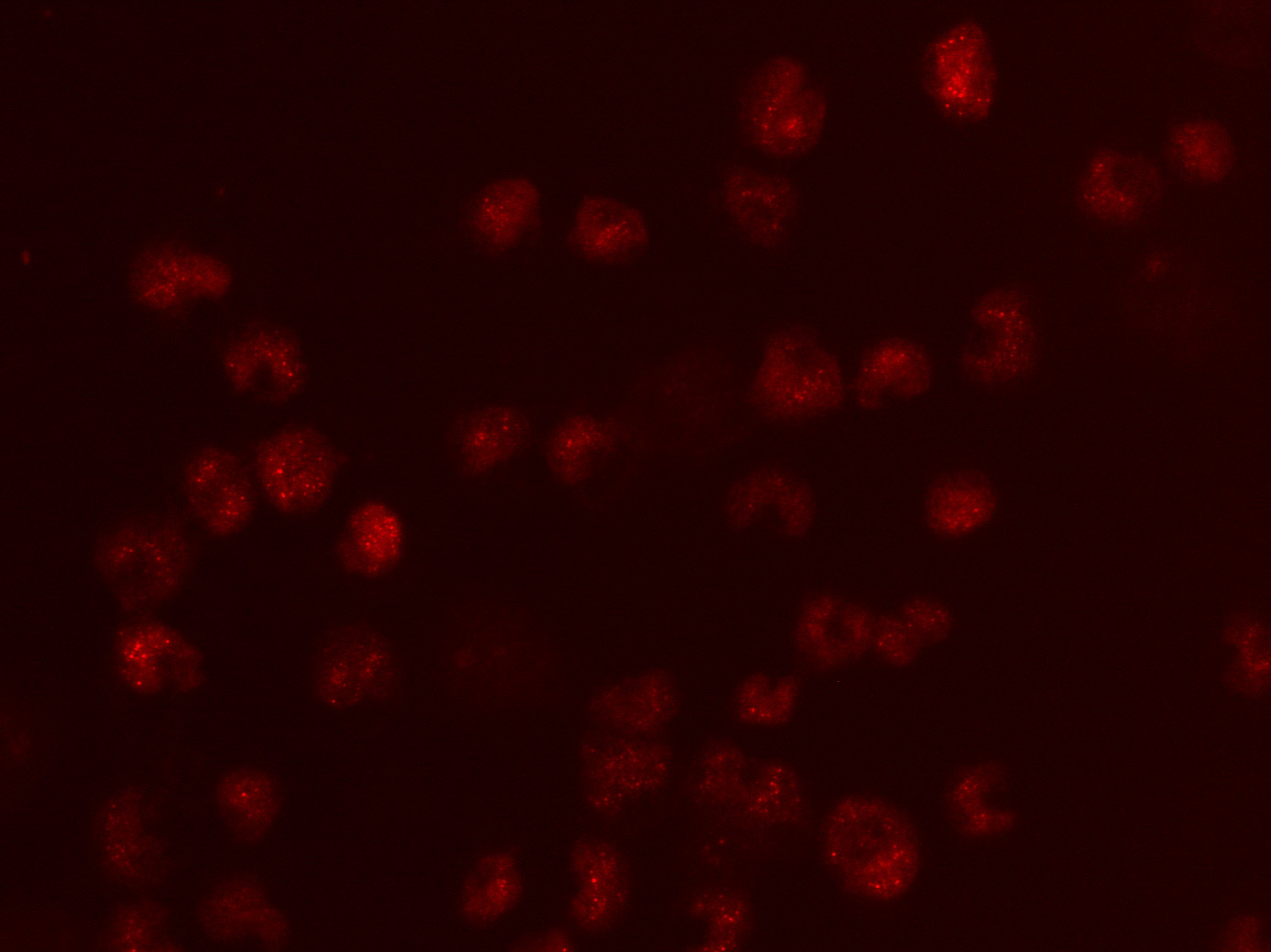

Supplement: S5 File — (ZIP) [file pone.0233739.s005.zip › S5_File/SKMES Sens-4h-2012-0027_c2.JPG]

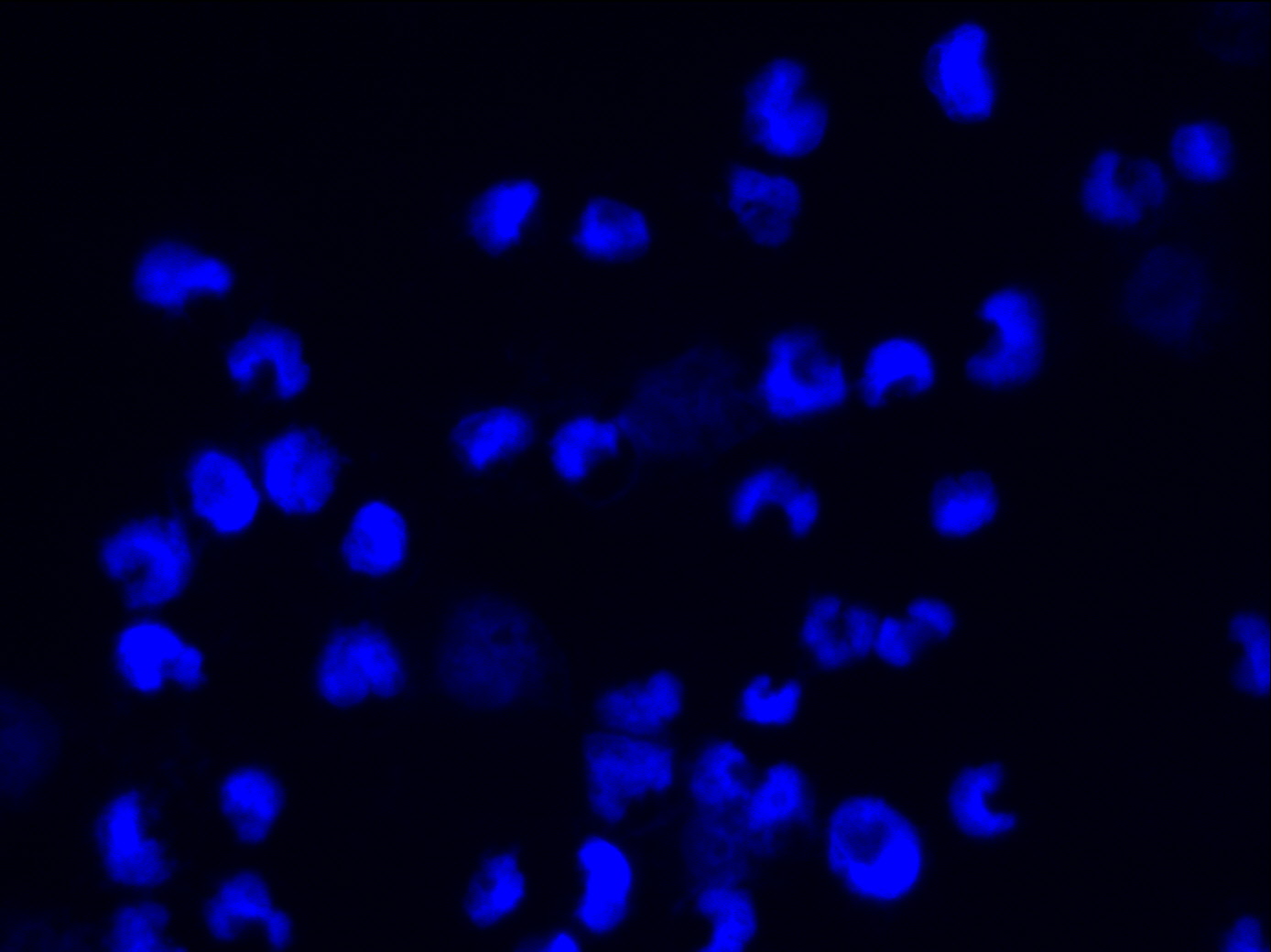

Supplement: S5 File — (ZIP) [file pone.0233739.s005.zip › S5_File/SKMES Sens-4h-2012-0027_c3.JPG]
